# The Significance of Mono‐ and Dual‐Effective Agents in the Development of New Antifungal Strategies

**DOI:** 10.1111/cbdd.70045

**Published:** 2025-01-22

**Authors:** Cengiz Zobi, Oztekin Algul

**Affiliations:** ^1^ Department of Pharmaceutical Chemistry, Faculty of Pharmacy Erzincan Binali Yildirim University Erzincan Turkiye; ^2^ Department of İliç Dursun Yildirim MYO Erzincan Binali Yildirim University Erzincan Turkiye; ^3^ Department of Pharmaceutical Chemistry, Faculty of Pharmacy Mersin University Mersin Turkiye

**Keywords:** antifungal agents, dual inhibitors, ergosterol, invasive fungal infection, lanosterol 14α‐demethylase, resistance mechanisms

## Abstract

Invasive fungal infections (IFIs) pose significant challenges in clinical settings, particularly due to their high morbidity and mortality rates. The rising incidence of these infections, coupled with increasing antifungal resistance, underscores the urgent need for novel therapeutic strategies. Current antifungal drugs target the fungal cell membrane, cell wall, or intracellular components, but resistance mechanisms such as altered drug‐target interactions, enhanced efflux, and adaptive cellular responses have diminished their efficacy. Recent research has highlighted the potential of dual inhibitors that simultaneously target multiple pathways or enzymes involved in fungal growth and survival. Combining pharmacophores, such as lanosterol 14α‐demethylase (CYP51), heat shock protein 90 (HSP90), histone deacetylase (HDAC), and squalene epoxidase (SE) inhibitors, has led to the development of compounds with enhanced antifungal activity and reduced resistance. This dual‐target approach, along with novel chemical scaffolds, not only represents a promising strategy for combating antifungal resistance but is also being utilized in the development of anticancer agents. This review explores the development of new antifungal agents that employ mono‐, dual‐, or multi‐target strategies to combat IFIs. We discuss emerging antifungal targets, resistance mechanisms, and innovative therapeutic approaches that offer hope in managing these challenging infections.

## Graphical abstract

The future of treating challenging fungal infections lies in novel therapies targeting new antifungal targets, overcoming resistance mechanisms, and exploring innovative dual inhibitors.

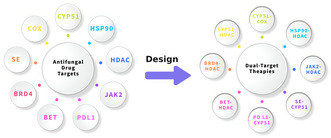

**Abbreviations**
5‐FC: 5‐flucytosine
5‐FU: 5‐fluorouracil
5‐FUDP: 5‐fluorouridine diphosphate
5‐FUMP: 5‐fluorouridine monophosphate
5‐FUTP: 5‐fluorouridine triphosphate
ABC: ATP‐binding cassette
Acetyl‐CoA: acetyl‐coenzyme A
AmB: amphotericin B
AML: myeloid leukemia
ASADH: aspartate semialdehyde dehydrogenase
AST: aspartate transaminase
BET: bromodomain and extra‐terminal
CAmB: amphotericin B cochleates
CATs: catalase enzymes
Class III: sirtuins
COF: covalent organic framework
COX: cyclooxygenase
CWI: cell wall integrity
CYP450: cytochrome P450
CYP51: lanosterol 14α‐demethylase; ERG11
DHAP: dihydroxyacetone phosphate
dTMP: deoxythymidine monophosphate
dUMP: deoxyuridine monophosphate
EMA: European Medicines Agency
ERG: ergosterol
ERG1: squalene epoxidase
ERG1: squalene monooxygenase, squalene epoxidase
ERG11: lanosterole 14‐demethylase; CYP51
ERG1p: squalene synthase
ERG24: C‐14 reductase
ERG7: lanosterol synthase
ERG7: lanosterole synthase
FAD: flavin adenine dinucleotide
Farnesyl‐PP: farnesyl pyrophosphate
FBA: fructose bisphosphate aldolase
FBA: fructose‐1,6‐bisphosphate aldolase
FBDD: fragment‐based drug discovery
FBP: fructose‐1,6‐bisphosphate
FDA: U.S. Food and Drug Administration
FLC: fluconazole
GAP: glyceraldehyde 3‐phosphate
GPI: glycosylphosphatidylinositol
Gwt1: GPI‐anchored wall transfer protein 1
H2A: histone 2A
H2B: histone 2B
H_2_O_2_
: hydrojen peroxide
H3: histone 3
H4: histone 4
HATs: histone acetyltransferases
HDAC: histone deacetylase
HDAC6: histone deacetylase 6
HDACs: histone deacetylases
HOM3: aspartate kinase
HOM6: homoserine dehydrogenase
HSP90: heat shock protein 90
IDSA: The Infectious Diseases Society of America
IFIs: invasive fungal infections
ILV2: acetolactate synthase
ITC: itraconazole
KDACIs: KDAC inhibitors
KDACs: lysine deacetylases
MAPK: mitogen‐activated protein kinase
MET1: S‐adenosylmethionine synthase 1
MET13: methylenetetrahydrofolate reductase
MET15: homocysteine synthase
MET2: homoserine transacetylase
MET3: ATP sulfurylase
MET4: transcription factor protein
MET6: methionine synthase
MIC: minimum inhibitory concentration
PTCL: peripheral T‐cell lymphoma
SAHA: vorinostat
SE: squalene epoxidase
Sit1: siderophore iron transporter 1
STR3: cystathionine β‐lyase
THR1: homoserine kinase
THR4: threonine synthase
TS: thymidylate synthase
UPRTase: uracil phosphoribosyltransferase
VOR: voriconazole
VVC: vulvovaginal candidiasis

## 1 Introduction

Invasive fungal infections (IFIs) are infections caused by the invasion of fungi into deep tissues, leading to prolonged illnesses (Ramana et al. 2013). IFIs are recognized as emerging and developing diseases in medical practice, with their incidence steadily increasing globally. Each year, approximately 6.5 million cases of IFIs occur, resulting in about 3.8 million deaths (Denning 2024). Over the past two decades, there has been a significant rise in morbidity and mortality associated with IFIs, making them a global health concern (Ahmad and Asadzadeh 2023; Yan et al. 2023).

The most common fungal species responsible for IFIs include *Candida*, *Aspergillus*, *Cryptococcus*, and *Pneumocystis*. Other species, such as *Blastomyces*, *Histoplasma*, *Paracoccidioides*, and *Coccidioides*, can also cause severe systemic infections (Fang et al. 2023). IFIs are more prevalent among patients who have undergone organ transplantation, those receiving treatment in intensive care units, individuals undergoing immunosuppressive or chemotherapeutic treatments, and patients infected with HIV (Han et al. 2020). Additionally, individuals with immunodeficiency, the elderly, and patients with diabetes are at higher risk for these infections, which are often difficult to treat (Enoch et al. 2017; Ashley 2019). These infections are also associated with AIDS and the development of resistance to antifungal agents has been linked to the increased rates of organ and hematopoietic stem cell transplants (Wirth and Ishida 2020). Notably, a severe and sometimes fatal type of IFI, mucormycosis, emerged in India following the second wave of COVID‐19 (Wen et al. 2022). IFIs also pose significant health challenges for immuno‐compromised children. Pediatric patients, particularly those with severe illnesses, require different treatment and care approaches compared to adults. Invasive *Candida* and *Aspergillus* infections are the most common in pediatric patients. Invasive candidiasis is more prevalent in pediatric intensive care units, whereas invasive aspergillosis is typically seen in children with hematologic cancers and solid tumors, indicating the need for specialized management and treatment strategies for fungal infections in critically ill children (Hon et al. 2024). In patients with liver diseases, including those with decompensated cirrhosis, hepatitis, and those who have undergone liver transplantation, the risk of developing IFIs is high. Numerous factors, such as host immune dysfunction, barrier failures, malnutrition, and microbiome alterations, contribute to the increased risk of IFI development (Barros et al. 2023). Chronic obstructive pulmonary disease patients, those in intensive care units, and individuals with lung cancer or hematologic malignancies are also susceptible to IFIs, which can lead to death. Furthermore, fungal asthma is estimated to affect 11.5 million people annually and contributes to approximately 46,000 asthma‐related deaths. Recent studies have also linked IFIs to other bacterial and viral infections, including SARS‐CoV‐2, which increases susceptibility to IFIs among immunosuppressed patients (Song, Liang, and Liu 2020).

Since fungi and humans both possess eukaryotic cell structures, they share similarities in cellular architecture and metabolic pathways (Roemer and Krysan 2014). This similarity is a primary reason for the challenges encountered in treating fungal infections. However, specific differences between human and fungal cells, such as ergosterol (ERG) in the fungal cell membrane and glucan in the cell wall, serve as primary targets for antifungal treatments.

Currently, antifungal drugs used to treat IFIs are generally classified into polyenes, azoles, echinocandins, thiomidilent inhibitors, RNA synthetase inhibitors, and mitotic inhibitors. However, fungi quickly develop innate or acquired resistance to these drugs. As a result, research continues to identify new antifungal targets and develop drugs specific to these targets (Zhang, Bills, and An 2023). Studies on antifungal drug resistance have identified several effective strategies against resistance, including increasing membrane β‐glucan, enhancing tolerance through cellular stress, inhibiting biofilm formation, and suppressing ERG biosynthesis. Furthermore, current or emerging antifungal drug targets, such as acetyltransferases and deacetylases, fungal aspartate pathways, HSP90, CYP51, HDAC, SE, fructose bisphosphate aldolase (FBA), arachidonic acid pathways, and sulfite transporters, have gained importance in preventing resistance development.

Several single‐target drugs, such as suba‐itraconazole, VT‐1129, VT‐1161, and VT‐1598, and cell wall‐targeting drugs, such as amphotericin B cochleates (CAmB), ibrexafungerp, rezafungin, and fosmanogepix, as well as intracellular targeting drugs, including VL‐2397, T‐2307, MGCD290, and olorofim, are under intensive study. Among these, suba‐itraconazole, otesoconazole, isavuconazole, luliconazole, efinaconazole, ibrexafungerp, and rezafungin have received U.S. Food and Drug Administration (FDA) approval, whereas others are in various clinical trial phases. Ibrexafungerp, the most recently approved antifungal drug by the FDA, targets the β‐D‐glucan component of the fungal cell wall, like established echinocandins (Ghannoum et al. 2020). Moreover, numerous new antifungal drugs are undergoing clinical evaluation and approval processes at the FDA, such as fosmanogepix, a broad‐spectrum antifungal drug acquired by Pfizer from Amplyx Pharmaceuticals, which inhibits the fungal enzyme GPI‐anchored wall transfer protein 1 (Gwt1). Fosmanogepix disrupts fungal cell wall integrity, preventing the proliferation of major fungal pathogens like *Candida albicans* and *Aspergillus niger* (Wu et al. 2023). Similarly, olorofim, a novel oral antifungal drug designed to treat invasive aspergillosis and other rare fungal infections, selectively targets dihydroorotate dehydrogenase in the mitochondrial membrane of fungi, inhibiting pyrimidine biosynthesis and consequently DNA synthesis, cell growth, and division (Neoh et al. 2023). Rezafungin, a new‐generation echinocandin with broad‐spectrum activity against many fungal species, including some drug‐resistant strains, is a semi‐synthetic, long‐acting β‐1,3‐glucan synthase inhibitor developed for the treatment of candidemia, invasive candidiasis, and prevention of IFIs caused by *Candida*, *Aspergillus*, and *Pneumocystis* species in blood and bone marrow transplantation patients (Thompson et al. 2023) and has received FDA approval (Adeel et al. 2021).

Despite the development of these single‐target, effective drug molecules, rapid resistance development to these drugs necessitates alternative approaches. Among these, combination therapy has emerged as a particularly noteworthy strategy. Initially, combination therapies combining two or more drug molecules showed significant success; however, they faced challenges such as drug solubility, continued rapid resistance development, and diverse interactions. In particular, the critical need for dose adjustments to prevent drug toxicity, associated with increased risks of drug–drug interactions and side effects, has become apparent. To overcome these limitations, many research groups have focused on molecular hybridization to create multi‐target drugs instead of conventional drug combinations. ThFis shift has led to the design of multi‐target ligands capable of simultaneously targeting multiple sites essential to the fungal life cycle with a single molecule, leading to synergistic effects. This novel approach could potentially overcome challenges such as resistance development, limited pharmacokinetics, and poor patient compliance associated with single‐target drugs. These new multi‐target drug molecules have shown the potential to offer potent and specific therapies by minimizing the side effects of the “one drug, one target” paradigm, with the ability to reduce drug interactions, combat resistance, and improve pharmacokinetics. While this novel approach is considered promising against drug resistance, drug discovery focusing on “polypharmacology” or “multi‐target” agents, targeting multiple biological systems rather than a single target, is gaining prominence.

This review focuses on the development of new antifungal drugs effective against IFIs. It examines new antifungal targets and strategies to prevent drug resistance against current antifungal drugs. The review also addresses research on drug resistance using single‐ or multi‐target approaches. Additionally, it provides a comprehensive overview of the strategies developed to combat rapidly increasing fungal infections and innovations in antifungal therapy.

## 2 Antifungal Drugs

**FIGURE 1:**
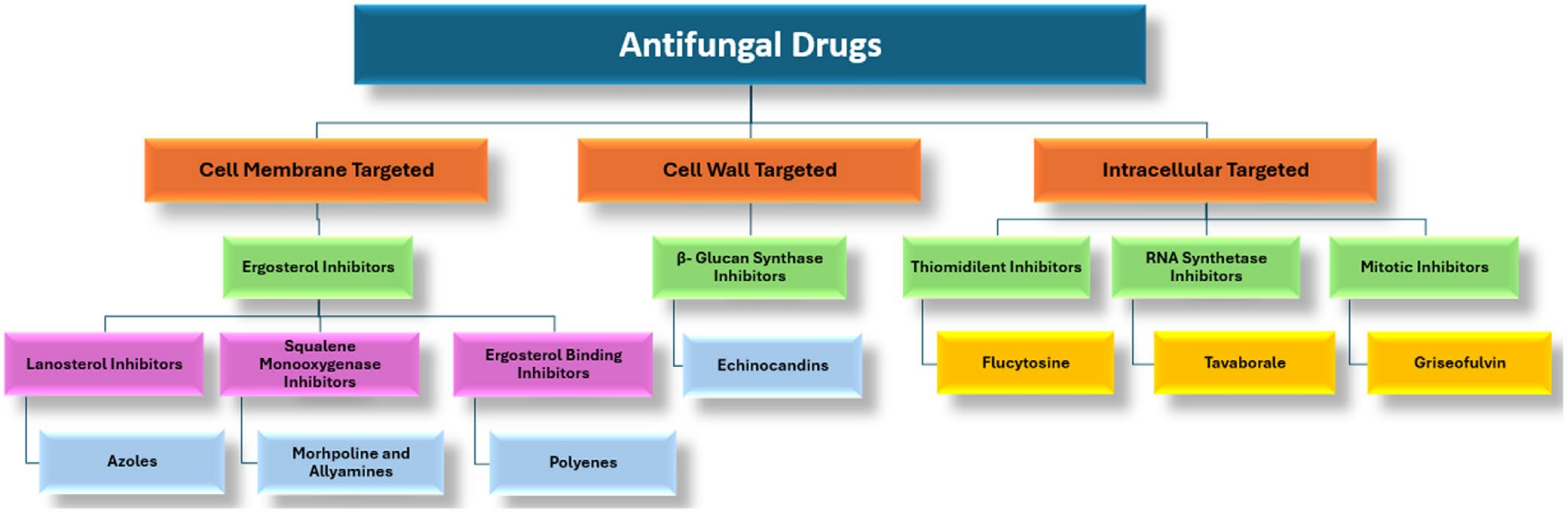
Classification of antifungal drugs.

Antifungal drugs possess diverse characteristics, particularly in terms of their spectrum of activity and pharmacological effects. These drugs can be classified based on their targets: the cell membrane, cell wall, or intracellular components (Figure 1). Cell membrane‐targeting antifungals include morpholines, allylamines (squalene monooxygenase inhibitors), azoles (lanosterol biosynthesis inhibitors), and polyenes (ERG‐binding inhibitors). These drugs disrupt the function of the fungal cell membrane by inhibiting the synthesis or function of ERG. On the other hand, cell wall‐targeting antifungals are primarily echinocandins (β‐glucan synthase inhibitors), which exert their effect by inhibiting β‐D‐glucan synthesis, thereby weakening the cell wall. Intracellular‐targeting antifungals include thymidylate inhibitors, which inhibit DNA and RNA synthesis; RNA synthase inhibitors, which inhibit protein synthesis; and mitotic inhibitors, which block cell division (Carmo et al. 2023).

### 2.1 Cell Membrane‐Targeting Antifungal Drugs

#### 2.1.1 Squalene Monooxygenase (Squalene Epoxidase; SE) Inhibitors

**FIGURE 2:**
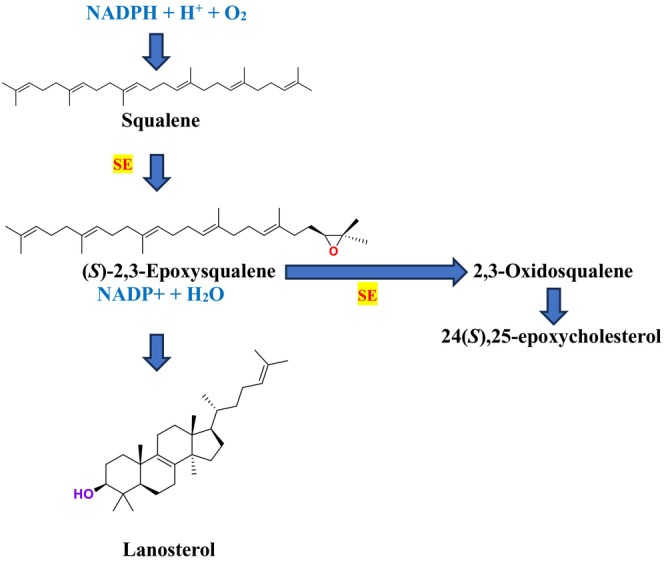
Conversion of squalene to 2,3‐oxidosqualene by SE in the presence of nicotinamide adenine dinucleotide phosphate (NADPH) and oxygen.

SE is a key enzyme in the mevalonate‐cholesterol pathway, playing a critical role in cellular physiological processes (Zhang et al. 2024). It is also vital for ERG synthesis in the fungal cell life cycle (Chua, Coates, and Brown 2020). Furthermore, SE provides the second highest rate‐limiting contribution to cholesterol synthesis, helping maintain cholesterol‐associated lipid structures (Li et al. 2024). In the ERG biosynthesis pathway, SE catalyzes the oxidation of squalene to (*S*)‐2,3‐epoxysqualene, facilitating the formation of lanosterol, an epoxysqualene derivative (Figure 2). Additionally, SE can convert 2,3‐epoxysqualene into diepoxysqualene, with the Shunt pathway's end product, 24(*S*),25‐epoxycholesterol, potentially regulating cholesterol metabolism.

Lanosterol is one of the most important components of the fungal cell membrane, making it a prominent target for antifungal activity. SE inhibitors target this enzyme, leading to the accumulation of squalene and the reduction or cessation of sterol biosynthesis, which is essential for the fungus's vital functions. This inhibition disrupts cell membrane integrity and function, making it particularly significant in the treatment of fungal infections. These inhibitors are also utilized in inhibiting the growth of cancer cells in humans (Zou et al. 2022).

**FIGURE 3:**
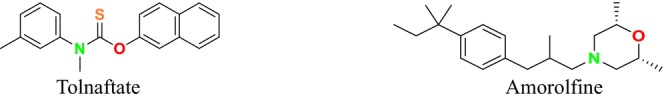
Tolnaftate and amorolfine chemical structure.

This group of inhibitors includes morpholines and allylamines. Morpholines are antifungals commonly used in agriculture and exhibit high toxicity in humans (Sanglard, Coste, and Ferrari 2009). Examples of this group include tolnaftate and amorolfine (Figure 3), which target C‐14 reductase (ERG24) in the ERG biosynthesis pathway (Figure 16). The mechanism of action of these drugs occurs on this enzyme (Bhattacharya, Esquivel, and White 2018).

Allylamines are another group of drugs targeting SE (ERG1) in the ERG biosynthesis pathways (Vanzolini and Magnani 2024). Allylamines inhibit ERG biosynthesis at an earlier stage than azoles (Hammoudi Halat et al. 2022). They block the synthesis of squalene epoxide, a precursor molecule of lanosterol involved in cell membrane formation, leading to the disruption of fungal cell membrane integrity (Figure 16; Andes et al. 2012).

**FIGURE 4:**
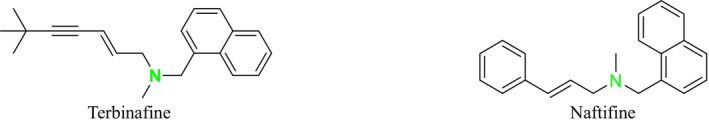
Terbinafine and Naftifine chemical structure.

Among antifungal drugs, allylamines include terbinafine and naftifine (Figure 4). Terbinafine is used in treating dermatophyte infections (Sanglard, Coste, and Ferrari 2009; Bhattacharya, Esquivel, and White 2018), whereas naftifine is highly selective against fungal enzymes and has minimal effects on mammalian cholesterol biosynthesis. Therefore, naftifine is effective in treating fungal infections with a low risk of harming human cells (Hammoudi Halat et al. 2022; Andes et al. 2012).

#### 2.1.2 Lanosterol Biosynthesis Inhibitors

**FIGURE 5:**
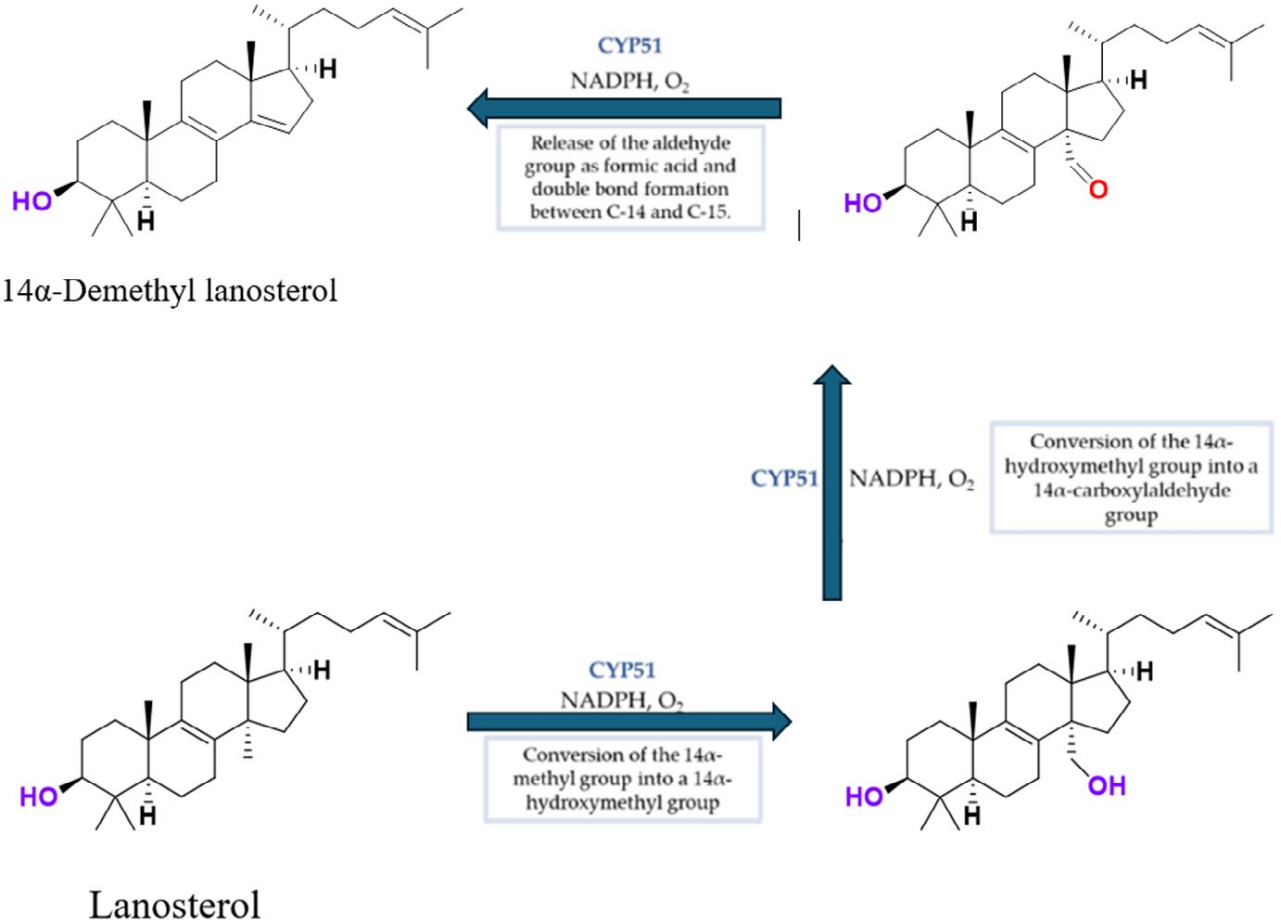
Mechanism by which the CYP51 enzyme catalyzes the conversion of the 14α‐methyl group on lanosterol to 14‐hydroxymethyl and 14α‐carboxaldehyde (Teixeira et al. 2022).

CYP51 (ERG11) is a member of the cytochrome P450 (CYP450) superfamily, a sterol commonly found in fungi, plants, and mammals (Kaluzhskiy et al. 2024). This enzyme plays a crucial role in ERG synthesis, catalyzing the removal of the 14α‐methyl group from sterol precursors. Lanosterol biosynthesis inhibitors specifically bind to this enzyme, inhibiting its catalytic activity (Yan et al. 2024). In clinical applications, azole drugs are used as lanosterol biosynthesis inhibitors and competitively inhibit the CYP51 enzyme (Figure 16), thereby inhibiting fungal membrane lipid synthesis (Mood et al. 2017). The CYP51 enzyme catalyzes the conversion of the 14α‐methyl group on lanosterol to 14‐hydroxymethyl and 14α‐carboxaldehyde. This group is released as formic acid, leading to the formation of a double bond between C‐14 and C‐15 (Figure 5; Teixeira et al. 2022). Azoles inhibit the synthesis of ERG, found in fungal cell membranes, increasing cell permeability, and causing various changes that halt fungal growth (Davood et al. 2023). The structural diversity, broad spectrum, administration route, and bioavailability of azole antifungals make them the preferred first‐line drugs in clinical applications (Pintye, Bacsó, and Kovács 2024). Based on the number of nitrogen atoms in the aromatic ring of azoles, they are classified into three groups: imidazoles, triazoles, and tetrazoles (García‐García and Borobia 2021; Howard et al. 2020; Zou et al. 2020). The drugs in these groups are listed in Table 1.

**TABLE 1:** Classification of azole group drugs based on the number of nitrogen atoms in their aromatic ring structure.

Imidazole derivatives	Triazole derivatives	Tetrazole derivatives
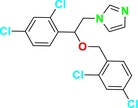 Miconazole	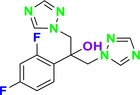 Fluconazole	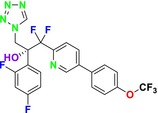 Quilseconazole
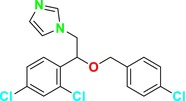 Econazole	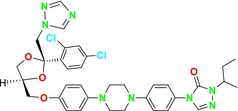 Itraconazole	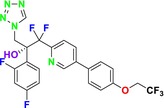 Oteseconazole
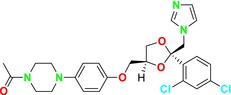 Ketoconazole	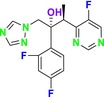 Voriconazole	
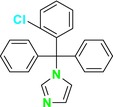 Clotrimazole	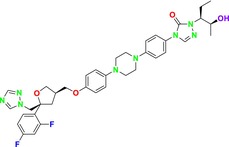 Posaconazole	
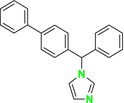 Bifonazole	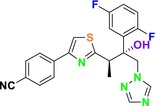 Isavuconazole	
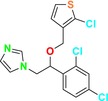 Tioconazole	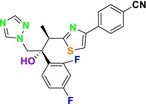 Ravuconazole	

#### 2.1.3 ERG Binding Inhibitors

**FIGURE 6:**
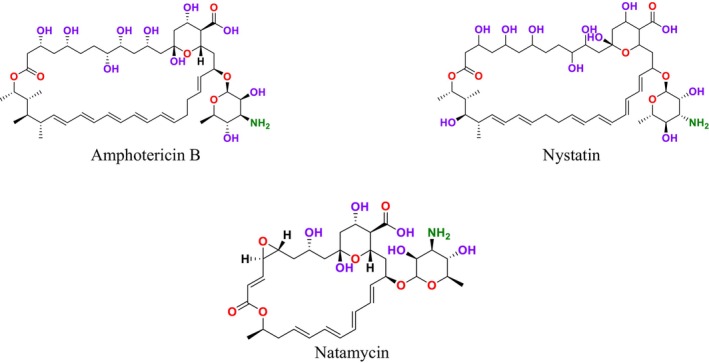
AmB, Nystatin and Natamycin chemical structure.

**FIGURE 7:**
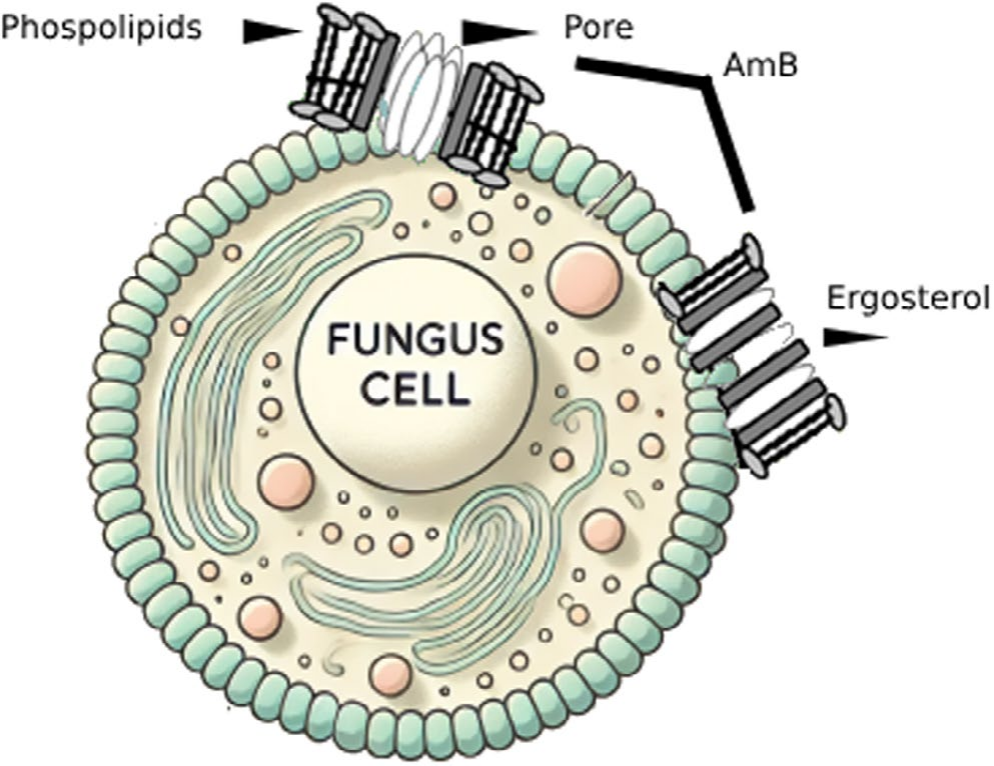
Mechanisms of AmB action on fungus cells (Mesa‐Arango, Scorzoni, and Zaragoza 2012).

Polyenes, which are ERG‐binding inhibitors, are natural products of a soil actinomycete called *Streptomyces nodosus* . These compounds irreversibly bind to ERG in the fungal cell membrane (Amangeldi et al. 2024). This binding leads to the formation of ion channels in the fungal cell membrane and the loss of protons and monovalent cations, causing membrane depolarization and concentration‐dependent cell death. Moreover, polyenes induce oxidative damage by causing the formation of free radicals, which also increases membrane permeability (Carmo et al. 2023). Polyenes are used as the main antifungal drugs against fungal infections caused by fungi such as *Aspergillus*, *Candida*, and *Cryptococcus*. Recent structural and biophysical studies have shown that polyenes bind to ERG in the cell membrane and extrude this sterol out of the membrane (Robbins, Caplan, and Cowen 2017). The extrusion of ERG from the membrane leads to membrane destabilization and protein dysfunction (Kristanc et al. 2019). This drug class targets ERG in the plasma membrane, binds to it, and forms pores (Efimova, Schagina, and Ostroumova 2014). Pore formation causes the rapid leakage of monovalent ions (K^+^, Na^+^, H^+^, and Cl^−^) and subsequently leads to fungal cell death. Examples of polyene drugs include AmB, nystatin, and natamycin (Figure 6). ERG is more sensitive to AmB than the common mammalian sterol cholesterol (Hamill 2013). The primary target of AmB is ERG in the fungal cell membrane; it forms aggregates that integrate into the lipid bilayer, which then form channels. This process makes the plasma membrane permeable, killing the fungal cell. This mechanism is how AmB exerts fungicidal activity in fungus cells (Figure 7; Posch et al. 2018).

Another drug in this group, nystatin, is a membrane‐active polyene macrolide produced by *Streptomyces noursei* strains and is only used topically (Lyu et al. 2016). Natamycin binds to ERG without altering cell membrane permeability and inhibits various ERG‐dependent membrane proteins that disrupt essential cellular processes such as glucose and amino acid transport and vacuolar fusion, which is the process by which vacuoles—large, fluid‐filled organelles within the cell—fuse with each other or with other cellular membranes (Carolus et al. 2020; Hanaoka et al. 2023). AmB, one of the molecules in this group, is an antifungal drug available on the market in nano formulation for treating systemic fungal infections (Alex et al. 2020).

### 2.2 Antifungal Agents Targeting the Cell Wall

#### 2.2.1 β‐Glucan Synthase Inhibitors

Echinocandins, a novel family of antifungal agents, are cyclic amphiphilic peptides with long lipophilic side chains. They act by inhibiting cell wall synthesis, which allows them to exhibit synergistic effects with other antifungal agents such as azoles or AmB, which target cell membrane synthesis (Mersinli 2020). These semi‐synthetic lipopeptides consist of a lipid acyl side chain attached to an N‐cyclic hexapeptide, produced by nonribosomal peptide synthesis, and include antifungal classes containing lipophilic side chains. Echinocandins are produced by filamentous fungi and are utilized as first‐line agents in the treatment of invasive mycoses (Hüttel 2021). Due to the absence of echinocandin targets in mammalian cells, these drugs are associated with fewer side effects, highlighting their importance in the treatment of fungal infections (Jiang et al. 2024).

**FIGURE 8:**
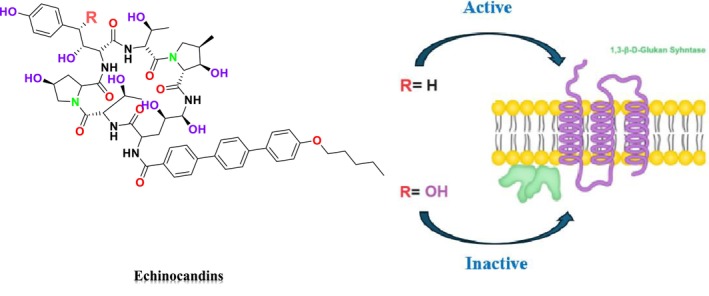
Inhibition of cell wall 1,3‐β‐D‐glucan synthesis by echinocandins (Logviniuk et al. 2022).

**FIGURE 9:**
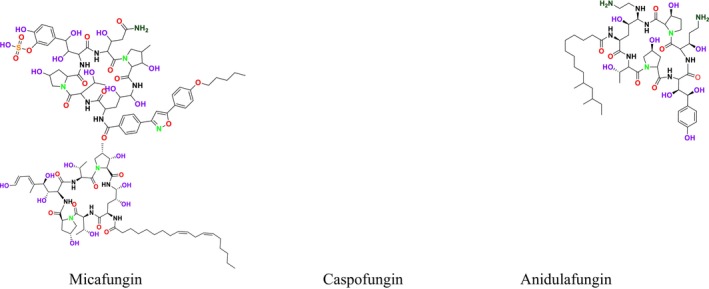
Micafungin, caspofungin, and anidulafungin chemical structures.

As β‐glucan synthase inhibitors, echinocandins disrupt the integrity of the fungal cell wall by inhibiting the enzyme β‐1,3‐glucan synthase, which is critical to produce β‐1,3‐glucan, a vital component of the fungal cell wall (Figure 8). This inhibition leads to osmotic stress and subsequent cell lysis (Apgar et al. 2021). The echinocandin class of antifungal agents includes micafungin, caspofungin (Mehravar et al. 2024), and anidulafungin (Figure 9; Prayag et al. 2024).

The β‐1,3‐D‐glucan synthase enzyme targeted by echinocandins comprises two subunits: Fksp and Rho1p. Fksp, the active site of the enzyme, is encoded by three genes (*FKS1*, *FKS2*, and *FKS3*). The transcription of *FKS1* is regulated by the cell cycle and is associated with cell wall remodeling, whereas *FKS2* transcription is calcineurin‐dependent (Denning 2003). Due to their high protein‐binding capacity (> 99%), echinocandins exhibit limited distribution in the central nervous system, ocular fluids, and urine, which is considered a beneficial pharmacokinetic profile (Lu et al. 2023).

Echinocandins have been approved by the FDA and the European Medicines Agency (EMA) for the treatment of candidiasis (Bassetti et al. 2022) and are effective in the prophylaxis and empirical treatment of IFIs. While they exhibit fungicidal activity against *Candida* species, they exert a fungistatic effect against *Aspergillus* species by inhibiting cell wall growth at the hyphal tip region (Nett and Andes 2016).

### 2.3 Intracellular Targeted Antifungal Agents

#### 2.3.1 Thymidylate Synthase Inhibitors (Pyrimidine Analogs)

Thymidylate synthase (TS) inhibitors function by binding to the active site of the TS enzyme, blocking RNA synthesis within the cell. TS is a crucial enzyme in DNA synthesis that facilitates the conversion of deoxyuridine monophosphate (dUMP) to deoxythymidine monophosphate (dTMP) (Sen and Karati 2024). These inhibitors compete with dUMP and the folate cofactor, thereby halting the methylation process, leading to the cessation of DNA synthesis and subsequent cell death. For instance, commonly used inhibitors such as 5‐fluorouracil (5‐FU) bind to TS drugs for the treatment of various fungal infections, including cryptococcal meningitis and candidiasis (Arendrup et al. 2013; Costantino et al. 2022).

**FIGURE 10:**
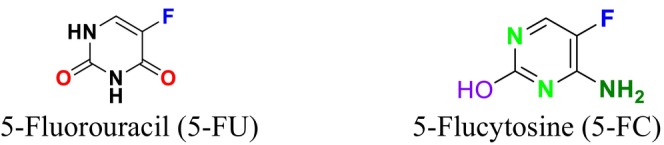
5‐Fluorouracil and 5‐flucytosine chemical structures.

**FIGURE 11:**
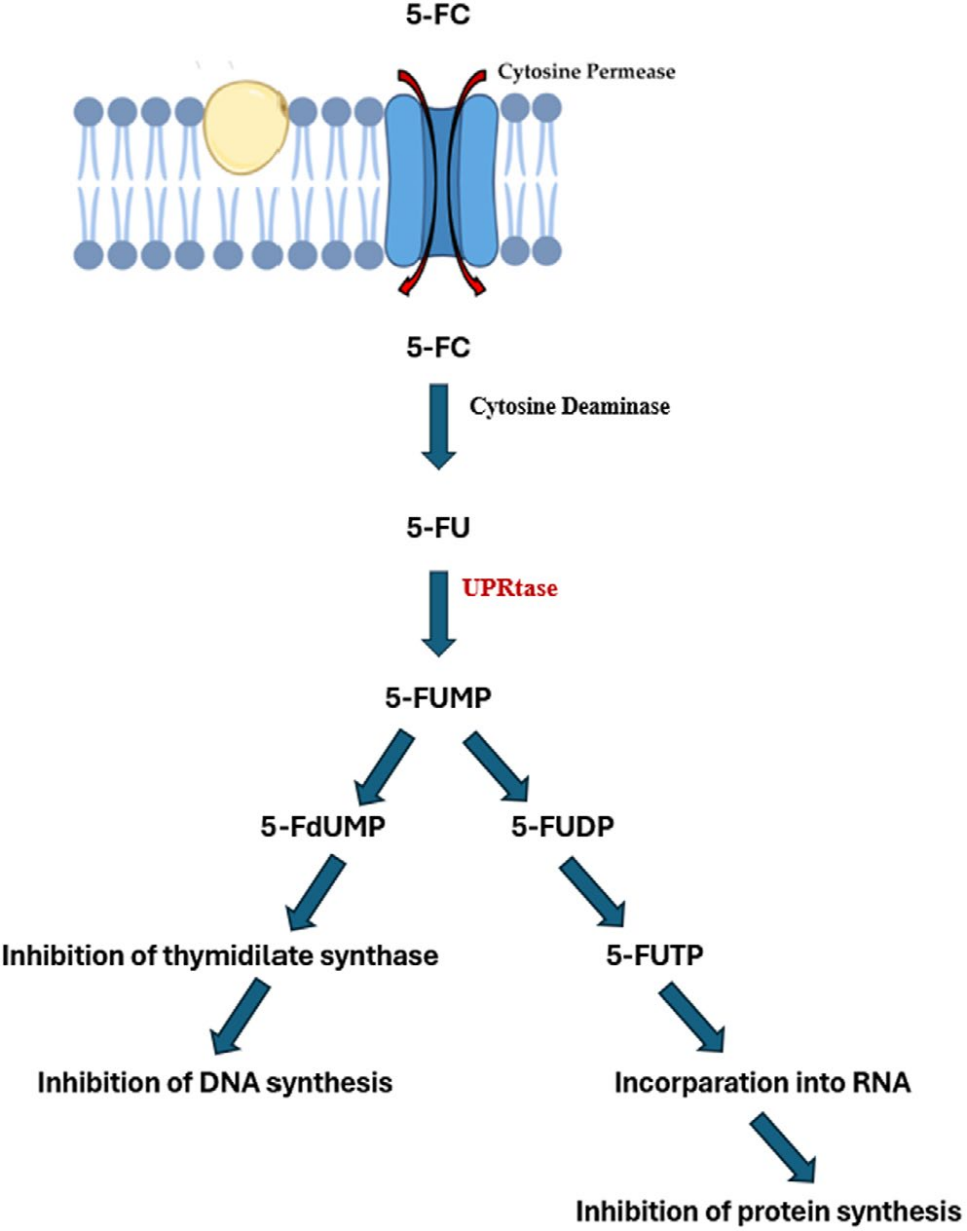
Mechanism of action of the antifungal agent 5‐FC (Vermes, Guchelaar, and Dankert 2000).

5‐Flucytosine (5‐FC) is another antifungal agent that interferes with nucleic acid biosynthesis (Figure 10). It is transported into the fungal cell via the cytosine permease enzyme, where it is converted into 5‐FU by the enzyme cytosine deaminase (Figure 10; Sigera and Denning 2023). 5‐FU is further transformed into 5‐fluorouridine monophosphate (5‐FUMP) by the enzyme uracil phosphoribosyltransferase (UPRTase) and subsequently into 5‐fluorouridine diphosphate (5‐FUDP) and 5‐fluorouridine triphosphate (5‐FUTP). Integration of 5‐FUTP into RNA synthesis results in faulty RNA production and inhibition of protein synthesis. Additionally, 5‐FUMP is converted into 5‐fluorodeoxyuridine monophosphate (5‐FdUMP), which inhibits the thymidylate synthase enzyme, thereby halting DNA synthesis. This mechanism effectively targets fungal cells, demonstrating antifungal activity (Figure 11; Osset‐Trénor, Pascual‐Ahuir, and Proft 2023). Moreover, 5‐FU is also employed as a chemotherapeutic agent in anticancer therapies (Lin and Huang 2024).

However, the frequent use of these drugs in treating IFIs has led to significant antifungal drug resistance, necessitating the development of new alternatives for clinical therapy (Yan et al. 2023).

#### 2.3.2 RNA Synthetase Inhibitors

**FIGURE 12:**
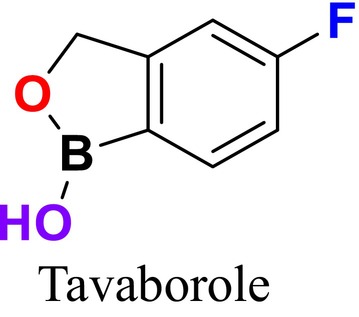
Tavaborole chemical structure.

RNA synthetase inhibitors are a critical class of drugs that inhibit protein synthesis by targeting aminoacyl‐tRNA synthetase enzymes. These enzymes play an essential role in protein synthesis by attaching amino acids to their corresponding tRNAs, facilitating the translation of the genetic code into proteins. By inhibiting these enzymes, RNA synthetase inhibitors cause fungal cell death (Carvalho 2023). Tavaborole, the first oxaborole antifungal agent approved by the FDA in July 2014, is used topically to treat onychomycosis, a fungal infection of the nails and nail beds caused by *Trichophyton rubrum* or *Trichophyton mentagrophytes* (Figure 12; Sharma and Sharma 2015; Arpitha et al. 2024). Tavaborole works by inhibiting leucyl‐tRNA synthetase, an enzyme involved in fungal protein synthesis, thus exerting its effect by blocking protein synthesis. In other words, it inhibits the cytosolic leucyl‐tRNA synthetase, also known as LeuRS, which is vital for the synthesis of essential proteins in fungi. The cessation of protein synthesis results in fungal cell inhibition and death (Pfizer 2018). Tavaborole's antifungal efficacy is attributed to the presence of a 5‐fluoro group, and its hydrophilicity is enhanced by replacing a 1‐phenyl group with a 1‐hydroxy group (Prajapati, Jain, and Bajpai 2024).

#### 2.3.3 Mitotic Inhibitors

**FIGURE 13:**
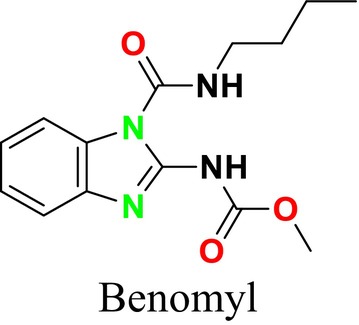
Benomyl chemical structure.

Mitotic inhibitors function by obstructing microtubule formation during mitosis in fungal cells, preventing the proper segregation of chromosomes and thereby halting cell division, leading to cell death (Flyway Pharmacy 2024). Benomyl, a broad‐spectrum antifungal agent used in the treatment of fungal infections, is also recognized for its potential anticancer properties (Figure 13; Wang et al. 2024). This benzimidazole derivative acts as a fungicide by targeting tubulin in fungal cells, inhibiting microtubule polymerization, preventing chromosome segregation, and thereby stopping cell division, leading to cell death. This mechanism effectively controls the spread of fungal pathogens, making it useful in treating infections (Bai et al. 2024).

**FIGURE 14:**
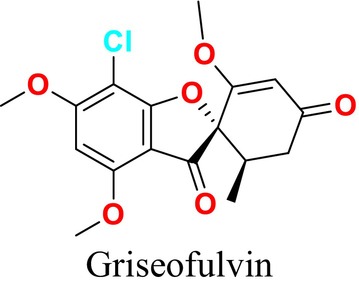
Griseofulvin chemical structure.

Griseofulvin, another mitotic inhibitor, was first isolated from *Penicillium griseofulvum* in 1939 and has been used as an antifungal agent (Figure 14; Aris et al. 2024). Since then, hundreds of griseofulvin analogs have been synthesized, some of which have been used in pesticide screening. Studies by Pan and colleagues have shown that 4′‐thiosemicarbazone‐griseofulvin strongly inhibits the mycelial growth of four fungi (*Fusarium oxysporum*, *Fusarium moniliforme*, *Fusarium solani*, and *Colletotrichum truncatum*; Bin Bai et al. 2023). Additionally, griseofulvin can be administered orally to treat tinea fungal infections (Hsiung et al. 2023). Clinically used in animals and humans for nearly 60 years, griseofulvin is poorly soluble in water, and its oral bioavailability is significantly affected by particle size (Ou et al. 2022). It is FDA‐approved for tinea capitis, although itraconazole and terbinafine have become more effective treatments for tinea capitis in adults (Elghblawi 2017). Nevertheless, due to its cost‐effectiveness and accessibility, griseofulvin remains the most commonly prescribed medication for treating tinea capitis in children. Researchers have found that griseofulvin and terbinafine have the highest clinical and complete cure rates among antifungal treatments for tinea capitis (Gupta et al. 2018). Griseofulvin is also indicated for onychomycosis (Kreijkamp‐Kaspers et al. 2017). Onychomycosis is primarily caused by *Tinea rubrum* and *Tinea interdigitale*, and there is high‐quality evidence supporting the efficacy of griseofulvin in achieving clinical and mycological improvement in onychomycosis treatment compared to placebo (Gupta, Foley, and Versteeg 2017).

## 3 Emerging Fungal Resistance to Antifungal Drugs and Mechanisms

Fungal resistance to antifungal drugs can arise from natural processes or from the overuse and misuse of antifungal agents. Immunocompromised individuals are at particularly high risk of developing fungal infections, which can lead to the emergence of drug‐resistant fungi. Some fungi, often referred to as “super fungi” exhibit resistance to standard antifungal treatments, further complicating treatment strategies (Cleveland Clinic 2021). The development of resistance to antifungal drugs can result in reduced or lost drug efficacy, leading to the need for higher dosages and increased frequency of administration. This escalation can be associated with adverse effects and toxicity (Sawant and Khan 2017). Effective treatment necessitates that each drug reaches sufficient concentrations at the site of infection. While the pharmacokinetics of many drugs are well understood, their penetration into all infection sites is not fully characterized. This lack of understanding can result in microorganisms that are not adequately exposed to the drugs, facilitating ongoing or new infections during treatment. Microbial resistance includes both primary resistances, where strains are naturally less susceptible to a specific antifungal agent, and secondary resistance, which develops in previously susceptible strains after treatment. These resistance mechanisms significantly contribute to therapeutic failures (Cowen et al. 2015).

Antifungal agents used in clinical settings are limited and are primarily classified into azoles, polyenes, echinocandins, and antimetabolites. Resistance to azoles and echinocandins, combined with the severe nephrotoxicity associated with polyenes, presents a major challenge (Mudenda 2024; Liu, Yuan, and Wang 2020). Therefore, a comprehensive understanding of antifungal drug resistance mechanisms is essential to elucidate resistance development (Sun, Chai, et al. 2023).

Fungal resistance mechanisms against antifungal drugs include reduced target affinity, alterations in membrane permeability, and decreased intracellular drug concentration due to efflux, all of which prevent the drug from binding to its target (Table 2; Bibi et al. 2021). Reduced drug accumulation is mediated by multidrug efflux transporters, membrane proteins that actively transport a variety of structurally and chemically diverse compounds out of the cell, playing a crucial role in drug resistance (Perlin 2015). Decreased binding affinity between the drug and its target can occur through mutations in genes encoding target enzymes, such as CYP51, which can reduce the binding capacity of azole drugs (Vandeputte, Ferrari, and Coste 2012). Adaptive resistance mechanisms include modifications or overexpression of drug targets, increased activity of multidrug transporters, and induction of stress responses in fungal cells (Lokeswari, Pal, and Naveen 2024). Additionally, biofilm formation hinders the interaction of antifungal drugs with the cell and upregulates azole efflux pumps such as Cdr1, Cdr2, and Mdr1, conferring drug resistance in fungi (Víglaš and Olejníková 2021). These resistance mechanisms significantly contribute to the rising incidence of antifungal‐resistant isolates in clinical settings (Odiba et al. 2022).

**TABLE 2:** Antifungal drugs and resistance mechanisms.

Target	Drugs and drug groups	Class	Mechanism of action	Resistance mechanism
**Cell wall**	Imidazoles Triazoles Tetraazoller	Azoles	**Azoles**: Inhibit CYP51 in the biosynthetic pathway of ERG in the cell membrane, increasing cell permeability (Davood et al. 2023)	**Mutations and Overexpression of ERG11, Cyp51A, and Cyp51B:** These genes encode the CYP51 enzyme, critical for ERG synthesis, leading to resistance (Rabaan et al. 2023)
**Cell wall**	AmB Nystatin Natamycn	Polyenes	**Polyenes:** Target ERG in the plasma membrane, binding to it and forming pores, which leads to cell death due to its fungicidal action (Efimova, Schagina, and Ostroumova 2014)	**ERG Disruption:** Mutations in ERG3 or ERG6 result in changes in sterol content, impacting cell membrane integrity (Yeğenoğlu 2012)
**Cell wall**	Terbinafine Naftifine	Allylamines	**Allylamines:** Inhibit the enzyme SE, blocking the synthesis of squalene, a precursor molecule to lanosterol involved in cell membrane formation, thereby compromising cell membrane integrity (Andes et al. 2012)	**Molecular Mechanism of Terbinafine Resistance:** Predominantly linked to point mutations in the SQLE target gene, resulting in a single amino acid substitution at one of four positions (Leu393, Phe397, Phe415, His440) in clinical strains of *Tinea rubrum* and *Tinea interdigitale* (Bhattacharjee and Dogra 2018; Rudramurthy et al. 2018; Khurana et al. 2018)
	Amorolfine Tolnaftate
Morpholines	**Morpholines:** Target the ERG biosynthetic enzyme C‐14 sterol reductase (ERG24p) (Bhattacharya, Esquivel, and White 2018)	**Molecular Mechanism of Morpholines Resistance:** It involves mutations in target enzymes and overexpression of efflux pumps (Lee, Robbins, and Cowen 2023)
**Cell membrane**	Caspofungin Micafungin Anidulafungin	Echinocandins	**Echinocandins:** Disrupt the integrity of the fungal cell wall, ultimately leading to cell lysis under osmotic stress by inhibiting β‐1,3‐glucan synthase, which produces the critical cell wall component β‐1,3‐glucan (Apgar et al. 2021)	**Echinocandin Resistance Mechanism:** Involves mutations in hotspot regions of β‐(1,3)‐D‐glucan synthase encoded by the *FKS1* gene, which reduces drug efficacy (Doorley 2023)
**Intracellular**	5‐Flucytosine	Pyrimidine Analogues	**5‐Flucytosine (5‐FC):** Inhibits nucleic acid biosynthesis (Houšť, Spížek, and Havlíček 2020)	**Alterations in Genes Responsible for Flucytosine Uptake and Conversion:** Mutations in FCY2, FCY1, and FUR1 genes, which are involved in the uptake and conversion of flucytosine, lead to resistance (Houšť, Spížek, and Havlíček 2020)

Antifungals targeting ERG synthesis, particularly azoles, bind to ERG, a fundamental component of fungal cell membranes, disrupting membrane integrity and halting fungal growth. Polyenes target ERG in the plasma membrane and are fungicidal; they bind to ERG and form pores (Bhattacharya, Sae‐Tia, and Fries 2020). The broad efficacy of these antifungals against a wide range of fungi and their low toxicity to the host make them a critical treatment option (Sant et al. 2016). However, resistance developed by fungi against these drugs necessitates a more detailed examination of the ERG biosynthesis mechanism and the development of new therapeutic strategies. Resistance to polyenes is associated with alterations in ERG3 and ERG6; disruptions in ERG3 and ERG6 decrease ERG levels and increase AmB resistance in *C. albicans* and *C. glabrata* (Vandeputte, Ferrari, and Coste 2012). Acquired resistance to AmB has been extensively studied in yeasts and is associated with the inhibition of both ERG3 and ERG11 genes in *C. albicans* (Posch et al. 2018). Moreover, azoles, which interfere with ERG synthesis, affect the products of the ERG11, ERG1, and ERG2 genes (Rodrigues 2018). Understanding these mechanisms is critical for developing new approaches to treating fungal infections.

**FIGURE 15:**
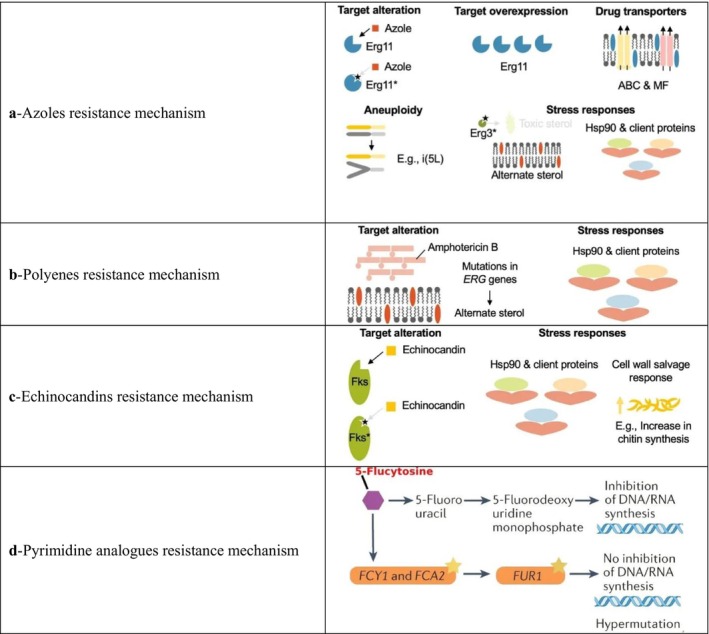
Antifungal drugs, their targets, and the mechanisms of resistance (Fisher et al. 2022).

Additionally, antifungal drugs, their targets, and the mechanisms of resistance to these drugs are presented in Figure 15. Antifungal drug resistance and tolerance acquisition vary depending on the mechanism of action of the drug used. Azole drug resistance is primarily due to the efflux of the drug from the fungal cell, a phenomenon particularly prevalent in *Candida* species. Moreover, changes in the sterol biosynthesis pathway, point mutations in the *CYP51A* gene, and promoter insertions can also contribute to resistance, especially in *Aspergillus fumigatus* . In other fungal species, such as *Cryptococcus neoformans* , chromosomal aneuploidy, and hypermutation often lead to the overexpression of drug targets and efflux pumps (Figure 15a).

Polyenes work by forming complexes with ERG, increasing membrane permeability. Resistance arises primarily from loss‐of‐function mutations in ERG biosynthesis genes, particularly in *Aspergillus* and *Candida* species. In *C. albicans* , the loss of both the ERG3 and ERG6 genes contributes to resistance. However, in *C. albicans* , the upregulation of ERG5, ERG6, and ERG25 genes is commonly associated with drug tolerance (Figure 15b). Cell membrane stress can also induce drug tolerance by affecting regulatory proteins such as HSP90.

Echinocandins inhibit the enzyme 1,3‐β‐D‐glucan synthase (*FKS1*), and mutations in this gene can lead to resistance in *Candida* and *Fusarium* species. Exposure to echinocandins can also induce cell wall stress through the inhibition of β‐glucan synthase, potentially leading to the indirect activation of Ca^2+^/calcineurin or HSP90/mTOR pathways, which are involved in drug tolerance (Figure 15c).

Pyrimidine analogs, such as 5‐FC, inhibit DNA and RNA synthesis. Resistance to these agents can arise through point mutations in the target gene *FCY1* in *Candida* species, whereas hypermutation in *Cryptococcus* species can also contribute to resistance against this class of drugs (Figure 15d; Fisher et al. 2022).

### 3.1 Azole Resistance

The development of resistance to azole antifungal drugs in fungal strains is an increasing concern in the treatment of fungal infections (Pérez‐Cantero et al. 2020). Azoles inhibit CYP51, an enzyme encoded by the ERG11 gene that plays a critical role in the biosynthesis of ERG, a fungus‐specific membrane sterol (Dladla et al. 2024). Azoles exert their antifungal effect by blocking lanosterol, the natural substrate of the enzyme, thereby disrupting the biosynthetic pathway. This process involves the binding of azoles to the ferric iron‐containing region of the enzyme (Odds, Brown, and Gow 2003). Some nonalbicans *Candida* species exhibit intrinsic resistance to azoles, potentially increasing the incidence of infections caused by these species. Additionally, numerous studies have documented the ability of *Candida* species to develop high levels of resistance to azole antifungals (Whaley et al. 2017).

In *A. fumigatus* , the most frequently reported mechanism of resistance involves alterations in the target site, with over 30 CYP51A mutations identified (Howard and Arendrup 2011). Azole resistance can arise from various mechanisms, including target site modifications, upregulation of efflux pumps, and alterations in metabolic pathways that reduce azole efficacy by removing the drugs from the target site. Understanding these resistance mechanisms is crucial for enhancing the effectiveness of azoles and improving the management of fungal infections. Azole resistance may develop through multiple mechanisms, including:

1. Mutations in the CYP51A gene, the target of azole drugs (Pérez‐Cantero et al. 2020).
2. Upregulation of multidrug transporters such as *CDR1*, *CDR2*, and *MDR1* (Joseph‐Horne and Hollomon 2006).
3. Alterations in sterol biosynthesis may decrease susceptibility to azoles (Joseph‐Horne and Hollomon 2006), including mutations in ERG biosynthesis genes such as ERG2, ERG3, ERG6, and ERG24 (Lee and Lee 2018).
4. Overexpression of the azole target gene ERG11 and the ABC transporter gene *AFR1* (Cowen et al. 2015).
5. Loss of heterozygosity (LOH) events in specific genomic regions that harbor homozygous azole resistance mutations (Lee, Robbins, and Cowen 2023).
6. Increased efflux pump activity, reducing intracellular drug levels (Nett and Andes 2016).
7. Biofilm formation, a significant virulence factor in fungal infections, occurs tigmotropically on biotic and abiotic surfaces and within mucosal layers. The biofilm formation process begins with adherence to the substrate, followed by the formation of filamentous hyphae and the accumulation of EPS, marking biofilm maturation (Padmavathi et al. 2024).

Resistance can also be acquired following exposure to azole drugs during medical treatment (Berger et al. 2017). Addressing this challenge requires the development of new classes of antifungal drugs (Lee, Robbins, and Cowen 2023).

### 3.2 Polyene Resistance

The ability of fungal strains to overcome the antifungal activity of polyene drugs such as AmB can arise through various mechanisms involving mutations in the ERG biosynthesis pathway (Fenton and John 2024). These mutations typically occur in the ERG3 or ERG6 genes, leading to alterations in sterol content (Yeğenoğlu 2012). Polyene resistance may also develop through the upregulation of multidrug transporters like ABC transporters. Resistance to polyene drugs can be acquired during medical treatment or through environmental exposure to these agents (Ghannoum and Rice 1999).

Polyene drugs, such as AmB, act like a sterol “sponge” that extracts ERG from the fungal cell membrane, forming extramembranous aggregates. Mutations in the ERG biosynthesis pathway lead to depletion of ERG and accumulation of alternative sterols, resulting in resistance. This can lead to the emergence of polyene‐resistant *Candida* and *Cryptococcus* isolates with relatively low ERG content (Dick, Merz, and Saral 1980). Resistance to AmB has also been associated with increased catalase activity and reduced sensitivity to oxidative damage (Sokol‐Anderson, Brajtburg, and Medoff 1986). Stress responses mediated by HSP90 are also critical factors in the development of resistance to AmB, similar to other antifungal agents (Lee, Robbins, and Cowen 2023). The emergence of polyene resistance highlights the need for the development of new antifungal drug classes (Whaley et al. 2017).

### 3.3 Echinocandin Resistance

Mutations in the *FKS1* and *FKS2* genes are key mechanisms underlying echinocandin resistance (Misas et al. 2024). These genes encode the catalytic subunits of the β‐(1,3)‐D‐glucan synthase enzyme, which is essential to produce a major component of the fungal cell wall (De Francesco 2023). Mutations in the *FKS1* gene can lead to changes in the target enzyme of echinocandins, resulting in resistance to these drugs, which are commonly used in the treatment of various fungal diseases (Doorley 2023).

These mutations typically occur in hotspot regions of the enzyme, leading to amino acid substitutions that affect the drug‐binding site and reduce the drug's efficacy. In some fungal species, such as *C. glabrata* , resistance has been associated with mutations in both the *FKS1* and *FKS2* genes. These mutations result in high minimum inhibitory concentrations (MICs) for cells exposed to echinocandins and significant reductions in glucan synthase sensitivity to the drug, leading to treatment failures and clinical challenges (Lee and Lee 2018).

Combatting echinocandin resistance also involves managing cell wall salvage mechanisms and stress responses, which are critical for maintaining cell wall integrity and responding to echinocandin‐induced stress. Factors such as molecular chaperone HSP90, HSP90 client proteins, and genes regulating cell wall salvage signaling are crucial for these processes (Lee, Robbins, and Cowen 2023). Specific mutations in the *FKS* genes that encode the catalytic subunits of glucan synthase can lead to poor pharmacodynamic responses and reduced clinical outcomes (Pristov and Ghannoum 2019). Thus, understanding the impact of *FKS* gene mutations on echinocandin resistance is a vital area of research in the fight against antifungal resistance and the development of new treatment strategies (Perlin 2015).

### 3.4 Pyrimidine Analog Resistance

Despite being an effective antifungal agent against various *Candida* species, resistance to flucytosine commonly develops when used as monotherapy (Yeğenoğlu 2012). This resistance is primarily associated with the loss of cytosine permease, loss of cytosine deaminase activity, and loss of UPRTase activity. Mutations in the *FCY1* and *FCY2* genes, which encode UPRTase, can render flucytosine ineffective. The resistance rate to flucytosine in *C. albicans* isolates has been reported to be around 10%, largely due to reduced uptake of the drug into the cell via cytosine permease.

Another aspect of flucytosine resistance involves mutations in enzymes responsible for converting the drug into its toxic metabolites, 5‐FU and 5‐FUMP, during treatment. These mutations can affect the conversion of the drug within the fungal cell, contributing to resistance development. As a result, 5‐FC is often used in combination with a potent antifungal agent such as AmB to enhance efficacy and reduce the likelihood of resistance development (Şahiner and Altıntaş 2021).

## 4 Current and Emerging Targets in Antifungal Drug Development

Existing antifungal drugs primarily target the ERG biosynthesis pathway (azoles), disrupt ERG formation (polyenes), or inhibit cell wall synthesis (echinocandins). These targets are among the potential drug targets encoded by the genomes of fungal pathogens (Robbins, Wright, and Cowen 2016). An overview of the current and emerging targets in antifungal drug development is presented, emphasizing the need for innovative strategies to combat antifungal resistance and improve therapeutic outcomes (Table 3). By focusing on these specific targets, antifungal therapies can be developed to effectively combat fungal infections while minimizing the development of resistance.

**TABLE 3:** Current and emerging targets in antifungal drug development.

Target sites	Antifungal drug targets
Targets located in the cell membrane	Sulfite transporters ERG biosynthesis SE CYP51
Targets located within the cell	Fungal aspartate pathway Acetyltransferases and deacetylases HSP90 HDAC FBA

### 4.1 Targets in the Cell Membrane

#### 4.1.1 Sulfite Transporters

Most superficial fungal infections are caused by dermatophytes, a specialized group of filamentous fungi that exclusively infect keratinized host structures such as hair, skin, or nails, utilizing them as their sole source of nitrogen and carbon (Kröber et al. 2017; Zaugg et al. 2009). Dermatophytes and other filamentous fungi release sulfite as a reducing agent during keratin degradation. In the presence of sulfite, cystine in keratin is directly cleaved into cysteine and S‐sulfocysteine. As a result, these reduced proteins become susceptible to hydrolysis by various endoproteases and exoproteases secreted by fungi (Léchenne et al. 2007).

Sulfite is produced during cysteine metabolism and is secreted by dermatophytes and filamentous fungi using a sulfite efflux pump encoded by the *SSU1* gene. The high expression of *SSU1* is a characteristic feature of dermatophytes, which enables efficient degradation of hair and nails by stratum corneum fungi (Baldo et al. 2012). Sulfite transporters are considered a novel target for antifungal drugs in dermatology because inhibiting these transporters could prevent dermatophytes from hydrolyzing keratin. Notably, sulfite transporters are absent in humans, making them a highly selective target for antifungal therapy (Léchenne et al. 2007).

#### 4.1.2 ERG Biosynthesis

**FIGURE 16:**
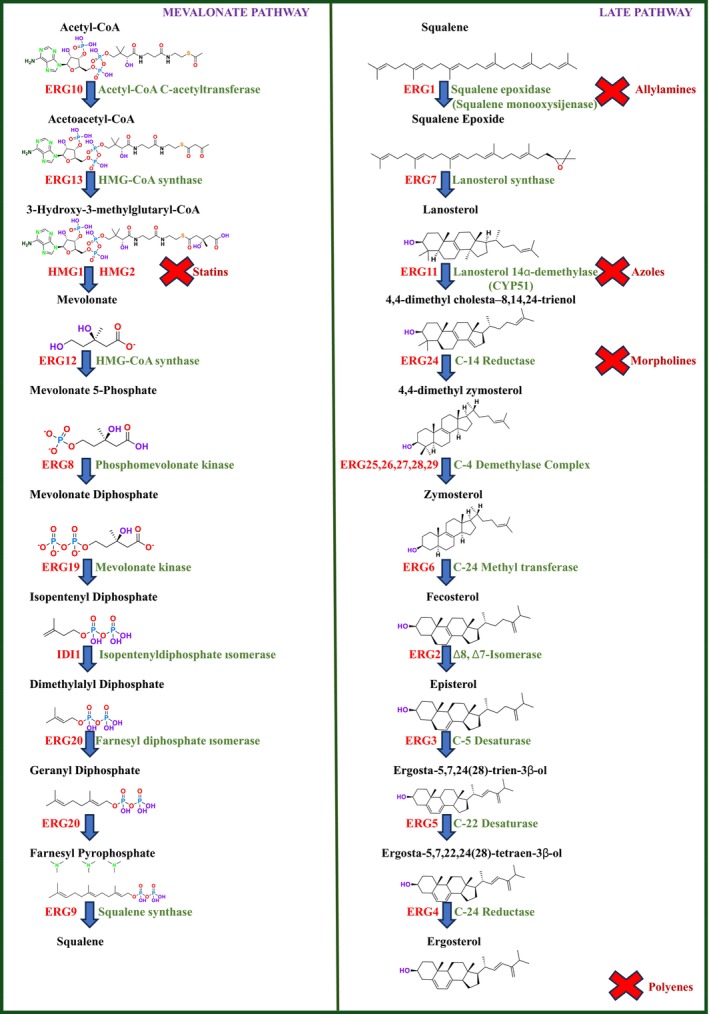
ERG biosynthesis pathway.

While cholesterol is the predominant sterol in humans, ERG is the primary sterol found in fungi (Zung et al. 2024). Sterols are essential for maintaining fungal cell integrity as they coordinate membrane heterogeneity, prevent water penetration, and preserve the integrity, rigidity, and fluidity of the plasma membrane. Antifungal treatments that target ERG biosynthesis include azoles, which inhibit CYP51, polyene drugs that disrupt ERG distribution across the membrane, and allylamines that inhibit SE (ERG1p) (Lv, Yan, and Jiang 2016). ERG biosynthesis involves a complex pathway with approximately 25 enzymes (Figure 16; Alcazar‐Fuoli et al. 2008). Among the enzymes involved in ERG biosynthesis are squalene SE, lanosterol synthase, C‐14 sterol reductase, C‐8 sterol isomerase, and C‐5,6 sterol desaturase. The ERG biosynthesis pathway is a highly complex process that requires significant energy and the participation of numerous enzymes (Hu et al. 2017; Jordá and Puig 2020).

The enzymes involved in ERG biosynthesis are categorized as essential or nonessential depending on whether the biosynthesis genes are necessary for fungal survival (Hu et al. 2017). Understanding the ERG biosynthesis pathway has led to the development of antifungal drugs that specifically target these enzymes. CYP450 proteins perform a three‐step reaction in the sterol biosynthesis pathway, leading to the production of cholesterol in animals, sitosterol in plants, and ERG in fungi (Schaller 2003; Dufourc 2008). Despite a billion years of divergent evolution between humans and fungi, there remains significant similarity between the genomes of humans and both beneficial and pathogenic fungi. Approximately one‐third of the genes found in the human genome have counterparts in fungal genomes, with more than 30% of human proteome amino acid sequences overlapping with those of fungi (Elias, Basu, and Fridman 2022).

Under aerobic conditions, fungal cells do not integrate external sterols; instead, they synthesize their own ERG to meet sterol requirements. Fungal ERG is synthesized via a highly conserved and complex pathway composed of three modules. The first module, conserved across all eukaryotes, leads to the formation of mevalonate from acetyl‐coenzyme A (acetyl‐CoA). The second module occurs in the vacuole and involves the formation of farnesyl pyrophosphate (farnesyl‐PP), an important intermediate in the biosynthesis of ubiquinone, dolichol, heme, and prenylated proteins. The third module, often referred to as the “terminal pathway,” involves ERG synthesis and sequential reactions primarily occurring in the endoplasmic reticulum membrane. Initially, two molecules of farnesyl‐PP are used to produce squalene. Subsequently, squalene is converted to lanosterol through the sequential actions of SE and lanosterol synthase (ERG7). In later stages, lanosterol is converted to zymosterol through a series of complex reactions, including demethylation, reduction, and desaturation catalyzed by also CYP51, C‐14 reductase (ERG24), and the C‐4 demethylation complex (ERG25‐ERG26‐ERG27; Liu et al. 2019; Ward et al. 2018). The ERG biosynthesis pathway plays a critical role in both fungal cell viability and resistance to antifungal agents.

Overexpression of ERG11 transcripts leads to decreased azole sensitivity and may result from increased numbers of gain‐of‐function mutations in the transcriptional regulator Upc2 or increased chromosome copy number. Mutations in ERG11 are frequently observed among azole‐resistant clinical fungal strains (Flowers et al. 2015). Molecular oxygen serves as an electron acceptor in the enzymatic steps catalyzed by ERG1, ERG11, ERG25, ERG3, and ERG5. Heme is directly involved with ERG biosynthesis, requiring oxygen and iron for ERG11 and ERG5 and functioning as a cytochrome b5 coenzyme for ERG25 and ERG3. Depletion of oxygen and iron is associated with reduced activity of these enzymes and alterations in sterol production. Disruptions in ERG biosynthesis lead to impairments in endocytosis, cell polarization, cell fusion, and cell wall organization (Joshua and Höfken 2017).

Deletion of many ERG genes in the terminal pathway is lethal to fungi under standard growth conditions without ERG supplementation. The only exception is the last five enzymes encoded by genes from ERG2 to ERG6, and possibly ERG28, due to their substrates having relatively similar physicochemical properties. The enzymes from ERG2 to ERG6 show low substrate specificity; hence, their deletion does not only lead to the accumulation of pathway intermediates but also to the buildup of sterol mixtures. The ERG6 mutation results in the accumulation of several sterols due to its substrate as well as the catalytic activities of ERG2, ERG3, and ERG5 on zymosterol. These ERG mutations exhibit defects in various cellular processes and alterations in resistance to specific stresses (Johnston, Moses, and Rosser 2020; Sokolov et al. 2019). Notably, the overexpression of each ERG gene leads to significant variability in tolerance to stress and antifungal drugs (Bhattacharya, Esquivel, and White 2018).

ERG6, ERG2, ERG5, and ERG4 are ERG enzymes not conserved in mammals. Among them, deletion and overexpression of ERG6 provoke the most compromised phenotypes, suggesting it could be a target for a new generation of antifungal agents (Kodedová and Sychrová 2015). Azoles bind to CYP51, causing depletion of intracellular ERG and accumulation of sterols in the fungal cell membrane, leading to the formation of toxic sterol intermediates, which halt growth and induce cell membrane stress (Cowen and Steinbach 2008). The antifungal activity of azole drugs is attributed to the depletion of ERG from the fungal membrane and the accumulation of the toxic product 14α‐methyl‐3,6‐diol, which leads to growth arrest. Modifications in the final stages of the ERG biosynthesis pathway through inactivation of the ERG3 gene can result in complete inactivation of C5 sterol desaturase and cause cross‐resistance to all azole drugs (Kanafani and Perfect 2008).

**FIGURE 17:**
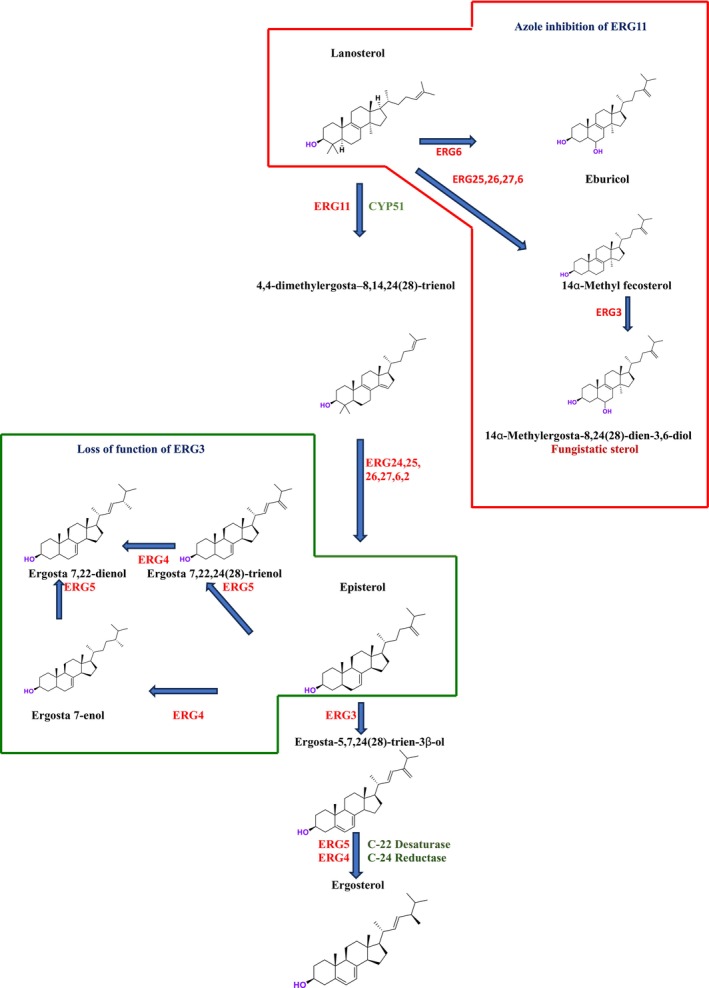
Alternative pathway for ERG biosynthesis (Vale‐Silva 2015).

A reduction or complete absence of ERG in the plasma membrane is a resistance mechanism among mutations in nonessential genes. For example, ERG3 mutation in *C. albicans* clinical isolates or ERG6 mutation in *C. glabrata* (Vandeputte, Ferrari, and Coste 2012). Azoles, the most used drugs to treat fungal infections, directly target ERG11 by binding to the iron atom in the enzyme's heme group. When ERG11 is inhibited, an alternative pathway catalyzed by ERG6, ERG25‐ERG26‐ERG27, and ERG3 is activated, leading to the formation of fungistatic 14α‐methylergosta 8–24 (28) dienol (Figure 17; Kelly et al. 1997). Consequently, mutations in the ERG6 and ERG3 genes contribute to the development of azole resistance (Bhattacharya, Esquivel, and White 2018; Sanglard et al. 2003). Loss of ERG3 function leads to the accumulation of C5‐C6 saturated sterols, which support fungal growth (as shown in the left panel). Upon exposure to azole drugs, ERG3 mutants accumulate these saturated sterols instead of fungistatic sterols, resulting in the fungus developing resistance to the drug (Figure 17).

##### 4.1.2.1 Squalene Epoxidase (SE; Squalene Monoxysigenase)

SE is a key enzyme in the ERG biosynthesis pathway, playing a critical role in cellular physiological processes. It converts squalene to 2,3‐epoxysqualene and catalyzes the first oxygenation step of the pathway (Figure 16; Zhang et al. 2024). As a rate‐limiting enzyme in ERG synthesis, SE is crucial for the biosynthesis of ERG. This enzyme is a flavin adenine dinucleotide (FAD)‐dependent epoxidase that catalyzes the stereo‐specific transformation of squalene to 2,3(S)‐oxidosqualene or dioxidosqualene (Figure 2). Overexpression of SE can elevate oxidative stress levels, potentially leading to the development of hepatocellular carcinoma. SE is also closely associated with extracellular signal‐regulated kinase pathways, lung cancer, and similar conditions. Therefore, ongoing research focuses on monitoring SE levels and distribution in cells and determining its biological functions (Zang et al. 2021).

During the formation of 2,3‐oxidosqualene from squalene, reduced NADPH‐hemoprotein reductase is oxidized in the presence of oxygen. FAD is an essential cofactor for SE activity. Due to the regio‐ and stereo‐specific epoxidation reaction catalyzed by SE, suitable molecular interactions are required for enzyme–substrate complex formation (Upadhyay et al. 2020). SE is commonly found in dermatophytes. Mutations in the SE gene can misdirect normal sterol formation, leading to fungal resistance to various drugs, including azoles and polyenes, affecting the fungal cell membrane. The SE gene contains two to three transcripts and two to three exons. Additionally, the SE gene in fungal groups of dermatophytes possesses a conserved structure for FAD‐dependent oxidoreductases and NADP binding (Muhammad Ismail, Ahmad, and Javed 2021).

The SE encoded by the ERG1 gene of *S. cerevisiae* catalyzes the epoxidation of squalene to 2,3(S)‐oxidosqualene and subsequently uses lanosterol as a substrate for cyclization. The enzyme exhibits very low specific activity, making it a rate‐limiting step in ERG biosynthesis (Leber et al. 2001). Terbinafine and naftifine are two drugs used in the treatment of fungal infections by inhibiting the SE enzyme. Terbinafine is available in both topical and oral forms and is particularly effective for treating nail fungal infections. Naftifine is generally used in topical form and is effective against skin fungal infections. Both drugs exert their antifungal effects by inhibiting ERG synthesis, a vital component of fungal cells (Mehta, Saini, and Bajaj 2023).

##### 4.1.2.2 Lanosterol 14‐α‐Demethylase (ERG11; CYP51)

One of the most well‐known targets of antifungal drugs is CYP51, a critical enzyme in the ERG biosynthesis pathway that affects the permeability of the fungal cell membrane (Lepesheva, Friggeri, and Waterman 2018). Azole antifungal agents, such as fluconazole (FLC), itraconazole (ITC), and voriconazole (VOR), primarily target this enzyme (Table 1). Azoles inhibit CYP51, thereby blocking ERG biosynthesis in fungal cell membranes and exhibiting fungistatic activity. The main molecular mechanisms of azole resistance include overexpression of CYP51, mutations in its structure, upregulation of efflux pumps, and fungal biofilm formation (Han et al. 2020). As a key component of ERG biosynthesis and an important part of the fungal life cycle, CYP51 belongs to the CYP450 superfamily. Selective inhibition of CYP51 leads to the depletion of ERG, a crucial component of the fungal cell wall, and the accumulation of lanosterol and other methylated sterols, ultimately resulting in the inhibition of fungal cell growth (Singh et al. 2023). Therefore, CYP51 is a prominent target for antifungal drug development.

ERG3 and ERG11 are among the most significant genes in the ERG biosynthesis pathway and play key roles in azole drug resistance (Figure 16; Zhou et al. 2018). In molds, such as *A. fumigatus* and *Penicillium digitatum*, the mechanisms of azole resistance involving CYP51 have been extensively analyzed. Structural alterations in CYP51, often referred to as “hot spot” mutations, serve as a resistance mechanism by causing structural changes that prevent azole binding (Bernhardt et al. 2018).

One disadvantage of azole drugs is that they are fungistatic rather than fungicidal, which contributes to the development of multiple resistance mechanisms. For example, point mutations in CYP51 (particularly around the enzyme's active site) can reduce the binding affinity of azoles to CYP51. Another azole resistance mechanism is the increased expression of ERG11 due to altered sterol composition in the plasma membrane of *C. albicans* , upregulation of the transcription factor UPC2, and overexpression of genes encoding multidrug resistance transporters (Derkacz, Bernat, and Krasowska 2022). These data underscore the importance of CYP51 in the ERG biosynthesis pathway.

### 4.2 Intracellular Targets

#### 4.2.1 Fungal Aspartate Pathway

**FIGURE 18:**
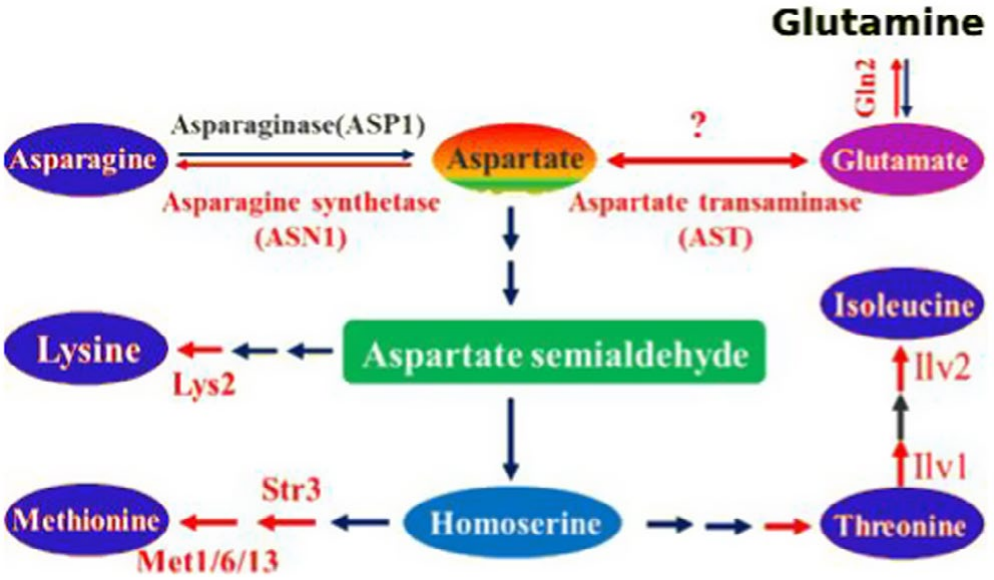
Aspartate pathway (Aron et al. 2021).

The fungal aspartate pathway is crucial for fungal survival because it is not present in mammals (Kuplińska and Rząd 2021). This makes the fungal aspartate pathway a highly suitable target for developing new antifungal agents (Yang et al. 2002; Pascon et al. 2004; Bareich, Nazi, and Wright 2003). In this pathway, most amino acids are derived from α‐keto acids and are typically synthesized by transamination from another amino acid, such as glutamate. Aminotransferase plays a key role in this process, whereas glutamate dehydrogenase catalyzes the reductive amination of α‐ketoglutarate to glutamate. This reaction is fundamental in the synthesis of members of the aspartate amino acid family, including threonine, lysine, methionine, isoleucine, asparagine, and aspartate. Aspartate transaminase (AST) plays a central role in the biosynthesis of aspartate, facilitating the interconversion of aspartate and oxaloacetate, thereby linking amino acid metabolism to the citric acid cycle by catalyzing the conversion between aspartate and glutamate. Amino acid metabolic pathways are integral to growth, conidiogenesis, and pathogenicity processes in pathogenic fungi (Figure 18; Aron et al. 2021).

Aspartate β‐semialdehyde dehydrogenase (ASADH) is a critical enzyme in the biosynthesis of amino acids in prokaryotes, fungi, and some higher plants. ASADH is a core component of the aspartate biosynthetic pathway, which is involved in the biosynthesis of essential amino acids and metabolites. ASADH plays a significant role in the diaminopimelate pathway leading to lysine biosynthesis (Kumar et al. 2024).

Key enzymes involved in methionine biosynthesis include cystathionine β‐lyase (STR3), cystathionine γ‐synthase (MET1), methionine synthase (MET6), and methylenetetrahydrofolate reductase (MET13) (Zhang, Fang, et al. 2022). Enzymes like ASADH convert substrate aspartyl phosphate to product aspartate semialdehyde (Dahal and Viola 2018). Other important enzymes in this pathway include homoserine transacetylase (MET2), ATP sulfurylase (MET3), transcription factor protein (MET4), methionine synthase (MET6), homocysteine synthase (MET15), aspartate kinase (HOM3), homoserine dehydrogenase (HOM6), homoserine kinase (THR1), threonine synthase (THR4), and acetolactate synthase (ILV2). Due to the absence of these enzymes in mammals, they present promising targets for the development of new antifungal agents (Su, Han, and Huang 2018). Thus, selected amino acid biosynthesis pathways are projected to offer new and effective strategies for antifungal therapy development.

**FIGURE 19:**
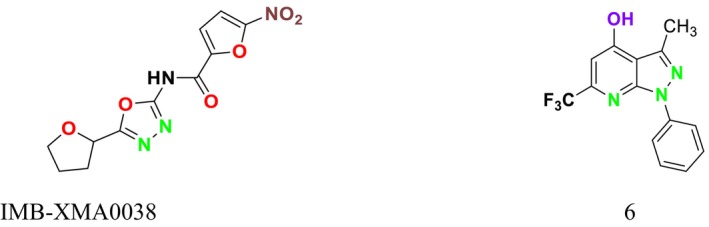
IMB‐XMA0038 and compounds 6 chemical structures.

In a study by Wang et al., a new antituberculosis compound, IMB‐XMA0038, was identified as targeting the ASADH enzyme of *Mycobacterium tuberculosis* (Figure 19). This compound was identified using a high‐throughput screening method with *Escherichia coli* type III aspartate kinase and demonstrated efficacy with an IC_50_ value of 0.59 μg/mL (Wang et al. 2021). In another study, De Pascale et al. screened the Prestwick, ChemDiv, and BIOMOL libraries and identified six compounds as homoserine kinase inhibitors. These compounds demonstrated effective antifungal activity against all tested fungal strains, including *Saccharomyces cerevisiae* , *Schizosaccharomyces pombe*, and *C. neoformans* . Further testing confirmed that compound 6 specifically targeted Thr1 in *S. pombe* and *S. cerevisiae* , inhibiting fungal growth (Figure 19). These results highlight the potential of Thr1 as a significant antifungal target for further research (De Pascale et al. 2011).

#### 4.2.2 Acetyltransferases and Deacetylases

Lysine acetylation in histones is an evolutionarily conserved and reversible posttranslational modification regulating protein functions in eukaryotes. First described by (Mukhopadhyay 2012), this process involves the transfer of an acetyl group from acetyl‐CoA to the ε‐amino side chain of a lysine residue on a protein. This modification occurs on both histone and nonhistone proteins. Acetylation of lysine residues in histones such as Histone 2A (H2A), Histone 2B (H2B), Histone 3 (H3), and Histone 4 (H4) generally results in the destabilization of DNA–histone interactions and an increase in transcriptional activity. This is because lysine acetylation neutralizes the positive charge of lysines, preventing salt bridge formation with the negatively charged phosphate backbone of DNA (Wang et al. 2020).

The reverse reaction, lysine deacetylation, is catalyzed by lysine deacetylases (KDACs), which include HDACs and sirtuins (or class III HDACs). This process affects protein structure, influencing enzyme activities, DNA‐binding affinities, and protein stability (Li, Ge, and Li 2020). The discovery that KDACs are vital for the viability of many filamentous fungi has led to the development of potent KDAC inhibitors (KDACIs), some of which are approved for treating various diseases. This underscores the importance of KDAC enzymes as potential target molecules in antifungal therapy (Bauer and Graessle 2021).

Catalase enzymes (CATs), present in living cells, play a critical role in breaking down hydrogen peroxide (H₂O₂) into water (H₂O) and oxygen (O₂) gas (Yuzugullu Karakus 2020). The evaluation of KDAC and CAT enzymes as novel targets for the treatment of fungal infections highlights the significance of protein acetylation in the biological processes of fungi. These enzymes are proposed as promising targets in antimicrobial therapies (Wassano et al. 2020).

**FIGURE 20:**
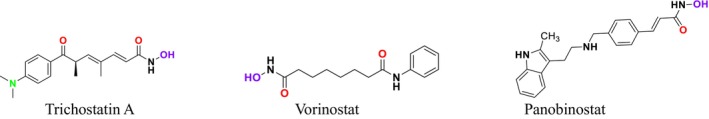
Trichostatin A, vorinostat, and panobinostat chemical structures.

Trichostatin A is a known KDAC inhibitor that has been shown to enhance the sensitivity of *Candida* species to azole‐derived antifungals (Figure 20). This effect may be related to trichostatin A's influence on ERG biosynthesis or its regulatory role in the SET3C KDAC complex (Li et al. 2021).

Vorinostat (SAHA) is a KDAC inhibitor that acts as a hypoxia‐activated prodrug, targeting cells under hypoxic conditions, which are often found in solid tumors, making SAHA particularly useful in cancer treatment (Figure 20). When used in combination with azoles, it exhibits a synergistic effect on *Aspergillus* species, especially on *A. fumigatus* biofilm and planktonic cells, enhancing the antifungal efficacy of azoles by suppressing HSP90 expression. Vorinostat's role as an HDAC inhibitor is crucial in reducing azole resistance, offering a potential approach for treating *Aspergillosis*‐induced infections (Tu, Yin, and Li 2020).

Panobinostat is another KDAC inhibitor designed to be activated under hypoxic conditions (Figure 20). Research on this drug focuses on its efficacy in targeting hypoxic tumor cells, particularly in preclinical trials aimed at tumors in low‐oxygen environments that are resistant to other treatment modalities (Skwarska et al. 2021).

#### 4.2.3 Heat Shock Proteins (HSP90)

In fungal pathogens, such as *C. albicans* and *A. fumigatus* , HSP90 plays a critical role in virulence and drug resistance. This function is mediated through the interaction of these evolutionarily conserved molecular chaperones with their co‐chaperones (O'Meara, Robbins, and Cowen 2017; Banerjee et al. 2021; Girstmair et al. 2019). Specifically, in *C. albicans* , heat shock proteins such as HSP90 and Hsp21 are closely associated with trehalose biosynthesis. Hsp21 has been shown to play a significant role in adapting to various environmental stresses by modulating trehalose homeostasis and the activation of the Cek1 kinase (Chen et al. 2020). Trehalose is a nonreducing glucose disaccharide that serves as an energy and carbon source in many organisms (Li, Xu, et al. 2023).

HSP90 stabilizes stress‐activated protein phosphatases and kinases, such as calcineurin and Pkc1, involved in mitogen‐activated protein kinase (MAPK) pathways, thereby contributing to drug resistance and virulence (O'Meara, Robbins, and Cowen 2017). Important proteins stabilized by HSP90 include calcineurin, a Ca^2+^‐calmodulin‐activated protein phosphatase, and several components of the PKC‐MAPK cell wall integrity cascade (Pkc1, Bck1, Mkk2, and Mkc1). Consequently, HSP90 inhibition blocks the activation of calcineurin‐dependent stress responses and PKC signaling, thereby eliminating tolerance and resistance to azoles and echinocandin drugs (Owens 2003; Singh et al. 2009; Caplan et al. 2018; Lafayette et al. 2010). Additionally, the emergence of polyene resistance in *Candida* is also dependent on HSP90, underscoring this protein's conserved role in resistance development against various antifungals (Vincent et al. 2013). Therefore, targeting HSP90 inhibition is considered a robust strategy for treating fungal infections and reducing antifungal drug resistance (Ancuceanu et al. 2022; Chatterjee and Tatu 2017; Gaziano et al. 2018).

Studies investigating antibody responses in patients infected with *C. albicans* and in animal models of infection have identified immunodominant antigens between 45 and 52 kDa. Among these antigens, a 48 kDa protein has been identified as enolase, and a 47 kDa protein has been identified as the carboxy‐terminal fragment of *C. albicans* HSP90, forming the basis of diagnostic tests (Matthews et al. 2003). HSP90 is a frequent target in cancer treatment research; however, due to issues of toxicity and immune suppression, this approach has yet to receive FDA approval. Given that HSP90 is ubiquitously present in all eukaryotic cells, developing HSP90 inhibitors specific to fungi is crucial for antifungal drug development strategies (Yin et al. 2022).

**FIGURE 21:**
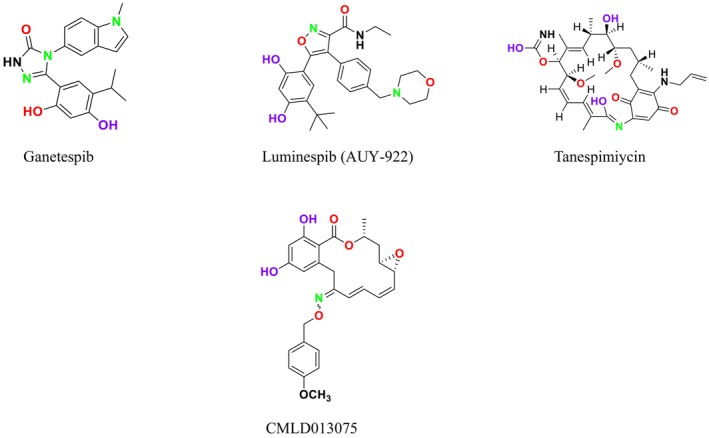
Ganetespib, luminespib, tanespimiycin, and CMLD013075 chemical structures.

Ganetespib, luminespib, and tanespimycin are HSP90 inhibitors evaluated in clinical trials for their efficacy against various cancer types (Figure 21). Ganetespib has been effective in treating lung cancer and other solid tumors, with fewer side effects. During phases I–III clinical trials, ganetespib demonstrated better tumor penetration and milder side effects compared to tanespimycin (Youssef et al. 2023).

Luminespib, a resorcinol derivative developed by the Cancer Research Institute in London, has been observed in preclinical studies to be active against tumor growth, angiogenesis, and metastasis. This compound has been subjected to clinical trials, particularly, for multiple myeloma and B‐cell malignancies (Zhang, Li, et al. 2022).

In a study by Huang et al., the researchers aimed to develop the first fungal‐selective HSP90 inhibitors based on semi‐synthetic oxime derivatives of radicicol and monocillin I resorcylate macrocyclic natural products that show activity against the *C. albicans* HSP90 isoform. Fungal selectivity is crucial for therapeutic applications, as existing inhibitors have limitations due to their action on host Hsp90, which presents various challenges in treating systemic infections. In their study, the oxime derivative monocillin CMLD013075 (Figure 21) exhibited 25‐fold higher binding selectivity to the nucleotide‐binding site of *C. albicans* HSP90 compared to its human ortholog, reduced fungal proliferation in whole‐cell assays, and was less toxic to human cells than the non‐selective compound radicicol (Huang et al. 2020).

#### 4.2.4 Histone Deacetylases (HDAC)

Histone acetylation and deacetylation are reversible processes catalyzed by two classes of enzymes: histone acetyltransferases (HATs) and HDACs. HATs catalyze the addition of acetyl groups to the ε‐amino groups of lysine side chains on histones and other proteins, using acetyl‐CoA as a cofactor (Liu, Zhang, et al. 2023; Simon et al. 2016). In contrast, HDACs remove acetyl groups from lysine residues on histones and nonhistone proteins, serving as an epigenetic enzyme family involved in deacetylation (Zwick et al. 2017; Zhou et al. 2020). The reversible posttranslational acetylation of conserved lysine residues on histones has been known to play a significant role in regulating gene expression for approximately 20 years.

While these enzymes are responsible for modifying histones through covalent modifications, bromodomains act as readers of the state of the acetylation, completing the epigenetic toolkit. HDACs are involved in cellular pathways that control cell shape and differentiation. Therefore, inhibitors of these enzymes are considered important candidates for cancer treatment. Vorinostat, panobinostat, belinostat, and romidepsin are relatively simple‐structured drugs recently approved by the FDA (Servatius and Kazmaier 2018).

**FIGURE 22:**
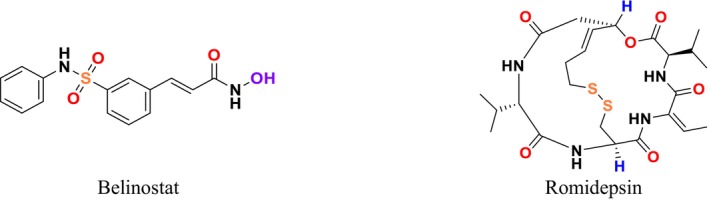
Belinostat and romidepsin chemical structures.

Belinostat, as an HDAC inhibitor, has been approved for the treatment of peripheral T‐cell lymphoma (PTCL) (Figure 22). In clinical trials, it has shown efficacy, particularly when used in combination with standard chemotherapy agents such as cyclophosphamide, doxorubicin, vincristine, and prednisone in a regimen known as Bel‐CHOP for newly diagnosed PTCL patients (Johnston et al. 2021).

Romidepsin has been extensively studied for its efficacy and applicability in various treatment contexts (Figure 22). Studies have particularly focused on using it in combination with other treatments to enhance its efficacy against T‐cell malignancies. For example, a phase I/II study explored the potential of increasing graft‐versus‐tumor effects through natural killer cell cytotoxicity by combining romidepsin with a conditioning regimen administered before allogeneic stem cell transplantation (Hosing et al. 2023).

In the co‐cultivation of certain fungal species with *Streptomyces* spp. 13F051, it has been shown that this bacterium, which produces HDAC inhibitors, can stimulate secondary metabolite production in fungi. This is significant for activating silent gene clusters and discovering new bioactive compounds (Hwang et al. 2023).

Class I HDACs are highly homologous to fungal HDAC Rpd3 and possess a completely conserved deacetylase domain compared to other classes. They are primarily located in the nucleus and exhibit strong deacetylase activity against histones at their sites of production. Additionally, they function as catalytic subunits in complexes with the same origin corepressors regulated by inositol phosphates to suppress target genes (Park and Kim 2020).

Class II HDACs show high homology with fungal HDAC I and exhibit a conserved deacetylase domain at their C‐terminus. Subdivided into class IIa HDACs (HDAC4, 5, 7, and 9), they contain a unique adapter domain at their N‐terminus that forms a binding site for the DNA‐binding transcription factor MEF2. The subsequent 3–4 phosphorylation sites serve as regulatory signals for the binding of 14‐3‐3 proteins, which shuttle between the cytoplasm and nucleus in response to various regulatory signals (Anthony and Muslin 2000).

Many studies have shown that Rpd3 HDAC plays a critical role in virulence (Bauer et al. 2019; Brandão et al. 2018; Hee Lee et al. 2021). Pho23 is a component of Rpd3 HDAC; however, its potential role in regulating virulence factors and its impact on virulence remain unclear in *C. albicans* . In a study by Du et al. (2023), Pho23 was identified in *C. albicans* and shown to be a member of Rpd3 HDAC in *S. cerevisiae* . By creating a Pho23 Δ/Δ mutant, they found that Pho23 regulates autophagy by upregulating the expression of ATG genes and increasing the number of autophagosomes. Disruption of Pho23 led to decreased cell wall stress resistance, reshaping of cell wall components, and reduced cell wall integrity (CWI) pathway activity. Additionally, Pho23 deletion reduced protease secretion and filamentous growth, indicating its necessity for virulence in *C. albicans* . Overall, Pho23 plays a significant role in the transcriptional regulation of many physiological processes in *C. albicans* (Du et al. 2023).

#### 4.2.5 Fructose‐1,6‐Bisphosphate Aldolase (FBA)

FBA is a key enzyme in the glycolysis and gluconeogenesis pathways. It catalyzes the reversible cleavage of fructose‐1,6‐bisphosphate (FBP) into glyceraldehyde 3‐phosphate (GAP) and dihydroxyacetone phosphate (DHAP), thereby providing ATP and a substrate for organisms (Han et al. 2017; Capodagli et al. 2014). There are two different classes of FBA with distinct catalytic mechanisms: FBA‐I and FBA‐II. FBA‐I is primarily found in higher organisms, whereas FBA‐II is found exclusively in fungi and bacterial cells (Mabiala‐Bassiloua et al. 2011; Capodagli et al. 2014). Notably, FBA‐II is often present in pathogenic microorganisms, such as fungi, bacteria, and cyanobacteria. Studies aimed at disrupting FBA‐II genes from various microbial species have shown that FBA‐II is essential for the viability of these organisms, making it an especially attractive target for treating pathogenic microorganisms (Wen et al. 2022).

Additionally, there are several fungi‐specific enzymes closely associated with the virulence of *C. albicans* , including acid trehalose, trehalose‐6‐phosphate synthase, trehalose‐6P phosphatase, enolase, class II fructose bisphosphate aldolases, pyruvate kinase, and glucosamine‐6‐phosphate synthase. Inhibitors against these specific enzymes have been shown to be applicable for candidiasis (Barelle et al. 2006).

**FIGURE 23:**
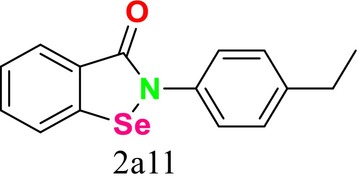
2a11 compound chemical structure.

Research on FBA inhibitors is still in its early stages, with most studies focusing on the structural design of inhibitors and understanding their mechanisms of action. For example, in a study by Wen et al. (2022), a specific FBA inhibitor, 2a11, demonstrated potent activity against resistant fungi, suggesting a promising strategy for developing antifungal drugs (Figure 23; Wen et al. 2022).

## 5 Development of New Single‐Target Antifungal Drugs

The development of potential new drugs for fungal infections, particularly IFIs, is challenging due to the eukaryotic structure of fungal cells. The widespread use of antifungal agents in treating IFIs has led to the emergence of clinical resistance. This resistance may not always be detected by in vitro susceptibility testing or in vivo animal models, posing a significant issue for researchers. Consequently, due to their clinical importance, there is ongoing research aimed at overcoming drug resistance by developing optimized derivatives and new hybridization approaches to enhance the efficacy of antifungals. This is a critical need to maintain the effectiveness of antifungal therapy (Teixeira et al. 2022).

For instance, rezafungin, approved by the FDA, has marked significant progress in the treatment of candidemia and invasive candidiasis (Connolly 2023). Additionally, researchers are developing new antifungal molecules by making structural modifications to AmB, which reduce toxicity while maintaining efficacy. This development is particularly promising because it aims to minimize one of the common side effects of current treatments—kidney damage (Touchstone 2023). Similar new developments represent a significant step forward in treating fungal infections that are increasingly resistant to existing treatments (Table 4).

**TABLE 4:** New single‐target antifungal drugs and compounds at various phase stages.

Target	Drugs name	Class	Mechanism of action	Clinical research phase	Fungus type of activity
**Cell membrane**	Suba‐Itraconazole ***Tolsura***	Azoles	Increasing the bioavailability of itraconazole	FDA approved	*Aspergillosis* spp., *Blastomyces dermatitidis*
	Otesoconazole ***Vivjoa*** **VT‐1161**		CYP51 inhibition	FDA approved	*Candida* spp.
	Quilseconazole **VT‐1129**			Faz I	*Cruptococcus neoformans*, *Candida* spp.
	**VT‐1598**				Molds, *Aspergillosis* spp., *Rhizopus arrhizus* and *Coccidioides*
	Opelconazole **PC‐945**			Faz III	*Aspergillosis* spp.
	Isavuconazole ***Cresemba***			FDA approved	Invasive aspergillosis, Mucormycosis
	Luliconazole ***Luzu*** **NND‐502**				Tinea pedis, Tinea cruris, and Tinea corporis.
	Efinaconazole ***Jublia*** **KP‐103**				Aspergillosis, Onychomycosis
	Albaconazole **UR‐9825**			Faz III	*C. albicans* , *C. neoformans* , *A. fumigatus*, and *N. gypsea*
	Ravuconazole **BMS‐207147**			Faz II	*Aspergillus fumigatus* , *Cryptococcus neoformans* , *Candida* spp.
	Aureobasidin A **LY 295337**		Inositol phosphorylceramide synthase inhibitors		*C. albicans* , *Cruptococcus neoformans*
	AmB Cochleat (CAmB)	Polyenes	ERG binding inhibitors		*C. albicans*
**Cell wall**	İbrexafungerp ***Brexafemme*** **SCY‐078**	Glucan synthase inhibitors	1,3‐β‐D‐glucan synthase inhibition	FDA approved	*Candida* spp.
	Rezafungin ***Rezzayo*** **CD101**				*Candida* spp., *Aspergillosis* spp.
	Fosmanogepix **APX001A**	Glycosyl phosphatidylnositol inhibitor	Glycosyl phosphatidylnositol inhibition	Faz III	Yeast, Molds, *Candida*, Cryptococcosis, *Coccidioides*, and *Aspergillosis* spp.
	Nikkomycin Z	Chitin synthase inhibitors	Chitin synthase inhibition	Faz II	*C. albicans and A. fumigatus*
**Intracellular**	**VL‐2397** ASP2397	Sidedefor	Iron chelation	Faz II	*A. fumigatus*
	**T‐2307**	Arylamidine	Inhibition of mitochondria synthesis	Faz I	*Candida* spp., Cryptococcosis, *and Aspergillosis* spp.
	**MGCD290**	HDAC inhibitors	HDAC inhibitions	Faz II	*Candida and Aspergillosis* spp.
	Olorofim **F901318**	Orotomidine synthase inhibitors	Inhibition of dihydroorotate dehydrogenase		*Aspergillosis and Scedosporium* spp.

### 5.1 Suba‐Itraconazole

**FIGURE 24:**
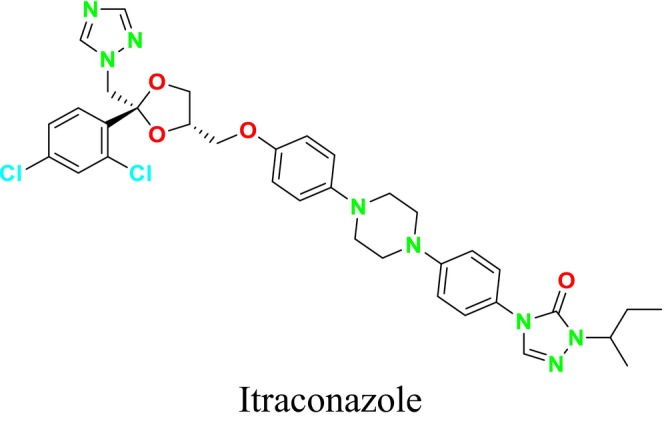
Itraconazole chemical structure.

While significant efforts have been made in identifying new antifungal compounds and classes, progress has also been achieved in optimizing existing antifungal agents. Itraconazole, a broad‐spectrum triazole, has clinical use limitations due to its poor bioavailability (Figure 24; Nield, Larsen, and Van Hal 2019; Liu et al. 2024). To address this issue, super bioavailability itraconazole (Suba‐Itraconazole), developed to enhance the drug's bioavailability, has been approved by the FDA. This formulation is used as a priority antifungal drug for treating allergic bronchopulmonary aspergillosis and other fungal infections in children (Abbotsford et al. 2021).

Suba‐Itraconazole, developed by Mayne Pharma Ltd., provides improved bioavailability through a solid dispersion in a pH‐dependent matrix and interferes with CYP450 activity to reduce ERG synthesis rates. This antifungal formulation has also demonstrated broad‐spectrum activity against *Blastomycosis* spp., *Histoplasmosis* spp., and *Aspergillosis* spp. (Houšť, Spížek, and Havlíček 2020; Gintjee, Donnelley, and Thompson 2020).

Traditional oral itraconazole has an approximate bioavailability of 55% when taken with food, but this decreases in patients with high gastric pH. The oral capsule formulation of Suba‐Itraconazole utilizes a solid dispersion of itraconazole in a pH‐dependent polymer matrix to enhance dissolution and absorption. Compared to the conventional oral formulation, it has been observed to significantly increase oral bioavailability by 173% (Abuhelwa et al. 2015). Moreover, this new formulation shows minimal food or acid effects on bioavailability, marking a substantial improvement over traditional itraconazole (Lindsay, Mudge, and Thompson 2018).

Suba‐Itraconazole represents a significant advancement in antifungal therapy by addressing the limitations of itraconazole's bioavailability and providing a more effective treatment option for various fungal infections.

### 5.2 Otesoconazole (VT‐1161), Quilsekonazol (VT‐1129) ve VT‐1598

**FIGURE 25:**
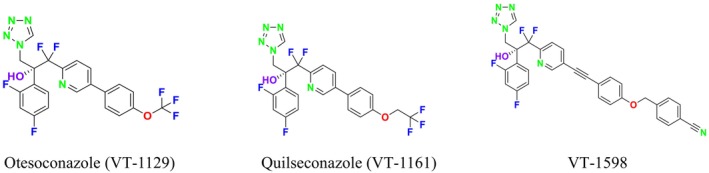
Otesoconazole, quilseconazole, and VT‐1598 chemical structures.

Tetrazole antifungals are new azole derivative compounds with a higher affinity for fungal cell CYP51 than human cytochrome enzymes. Currently used triazoles have higher side effect rates, toxicity, and drug–drug interactions due to their interactions with human CYP450 enzymes (Wiederhold, Xu, et al. 2018; Hoekstra et al. 2014). Therefore, the development of tetrazole antifungal drugs has become crucial in recent years to address these issues. One such development is otesoconazole (Vivjoa), which has been approved by the FDA and is marketed by Mycovia Pharmaceuticals (Figure 25; McCoy 2022). This drug is a highly selective antifungal designed to target fungal CYP51, helping to avoid off‐target toxicities commonly associated with azole drugs, such as concerns about pregnancy and miscarriage. Additionally, otesoconazole is highly effective against *Candida* species, including azole‐resistant strains, making it a safer and more effective antifungal option (Brand et al. 2021).

Another tetrazole derivative, quilseconazole, is a new fungal CYP51 inhibitor with potent in vitro activity against fluconazole‐resistant *Cryptococcus* species (Figure 25). This drug selectively inhibits the fungal CYP450 enzyme CYP51, more so than clinically used azole antifungals (Wiederhold, Shubitz, et al. 2018). Quilseconazole is an orally available antifungal developed by Mycovia Pharmaceuticals and has been investigated for its efficacy against cryptococcosis in mouse models. It has a relatively long half‐life of approximately 6 days, facilitating easy administration with a loading dose and maintenance dose (Wiederhold, Xu, et al. 2018).

In a separate study, Mycovia Pharmaceuticals reported on VT‐1598, the latest antifungal inhibitor of CYP51, encoded by the CYP450 gene. VT‐1598, currently in Phase I clinical trials, is effective against *Aspergillus*, *Coccidioides*, and *Rhizopus arrhizus* species (Figure 25; Hargrove et al. 2017). Additionally, VT‐1598 has demonstrated activity against fungi causing yeast infections, molds, and rare fungal infections. Wiederhold and colleagues investigated the efficacy of VT‐1598, evaluating its activity against *Coccidioides immitis* and *Coccidioides posadasii* in mouse models. Their study found that the MIC for fluconazole was 16 mcg/mL, whereas it was 0.5 and 1 mcg/mL for VT‐1598, respectively. Groups treated with VT‐1598 showed lower fungal loads and increased survival rates. Higher fungal loads were observed in groups treated with lower doses of VT‐1598 compared to those treated with fluconazole. These findings suggest that VT‐1598 may be effective against severe fungal infections (Wiederhold, Shubitz, et al. 2018).

In vitro results revealed that researchers tested VT‐1598 and otesoconazole (VT‐1161) antifungals against *C. albicans* strains, including those resistant to fluconazole, itraconazole, posaconazole, and voriconazole (Table 1). Both antifungals showed potent activity against many fungal strains, including fluconazole‐resistant strains with MIC values greater than 8 mcg/mL (Nishimoto et al. 2019).

### 5.3 Opelconazole

**FIGURE 26:**
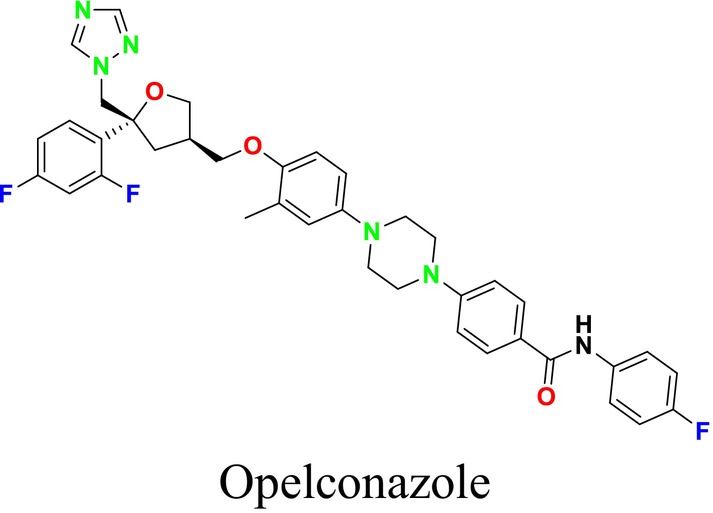
Opelconazole chemical structure.

Opelconazole (PC945) is a new triazole class antifungal designed for inhalation therapy (Figure 26). It provides targeted drug delivery to the lungs due to its low systemic exposure and high retention in lung tissue, enhancing efficacy while minimizing side effects and drug interactions (Kriegl et al. 2024). Opelconazole has the potential to be beneficial for a variety of *Aspergillosis*‐related infections, including chronic pulmonary aspergillosis, cystic fibrosis, severe asthma, allergic bronchopulmonary aspergillosis, chronic obstructive pulmonary disease, severe influenza infections, and lung infections associated with post‐COVID‐19 conditions.

Opelconazole is reported to be well tolerated, and its safety and efficacy are currently being investigated in a Phase III clinical trial for patients resistant to invasive pulmonary aspergillosis. The Phase II prophylactic safety study in lung transplant recipients has recently completed enrollment, with results expected in early 2024 (Pulmocide Ltd 2023). Pulmocide, a biopharmaceutical company focused on developing new treatment options for patients with chronic lung disease, has received Orphan Drug, Fast Track, and Qualified Infectious Disease Product designations from the FDA for opelconazole for the treatment of invasive pulmonary aspergillosis (Pulmocide Ltd 2021).

### 5.4 Isavuconazole and Its Clinical Application

**FIGURE 27:**
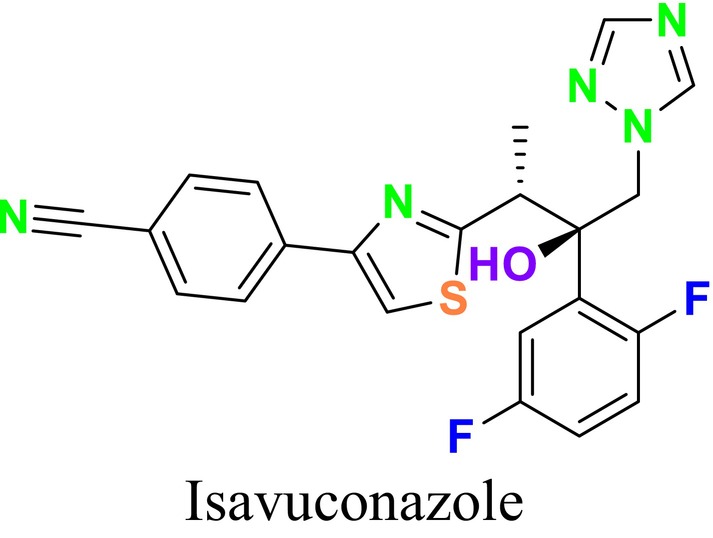
Isavuconazole chemical structure.

Isavuconazonium sulfate, the active component of isavuconazole, is rapidly converted to isavuconazole in the body through the action of esterases (Figure 27). As a potent CYP51 enzyme inhibitor, this drug exhibits high bioavailability and can be administered at the same dosage both orally and intravenously, offering significant flexibility in clinical use. Isavuconazole has a broad tissue distribution and binds extensively to plasma proteins, with approximately 98% binding affinity (Ellsworth and Ostrosky‐Zeichner 2020).

Belonging to the triazole class of antifungal agents, isavuconazole was approved by the FDA in 2015 for the treatment of invasive aspergillosis and mucormycosis. Research conducted in 2024 indicates that isavuconazole is effective for both prophylactic and therapeutic purposes against various IFIs. It has been shown to be safe across a wide patient population, including those who have exhibited toxicity to previous triazole therapies. Notably, therapeutic drug monitoring and dose adjustments were necessary in 37.7% of patients (Ergün et al. 2024).

### 5.5 Luliconazole

**FIGURE 28:**
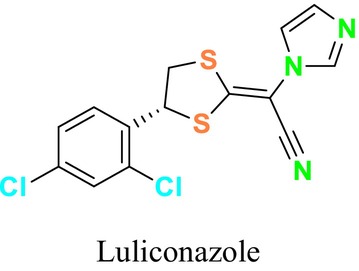
Luliconazole chemical structure.

Luliconazole, an antifungal agent approved by the FDA in 2013, is used topically to treat fungal infections such as tinea pedis, tinea cruris, and tinea corporis, which are caused by *Trichophyton rubrum* and *Epidermophyton floccosum* organisms (Figure 28; Drugs.com 2024). As a new member of the imidazole class of antifungal drugs, luliconazole inhibits ERG synthesis. The 1% topical formulation is utilized for the treatment of superficial fungal infections like tinea pedis, whereas the 5% formulation is indicated for onychomycosis treatment.

Luliconazole demonstrates potent antifungal activity not only against the *Trichophyton* species responsible for superficial fungal infections but also against nondermatophyte fungi such as *Aspergillus* and *Fusarium* species. It exhibits strong antifungal activity against the *Fusarium* complex isolated from patients with fungal keratitis and *Aspergillus* and *Fusarium* species isolated from fungal keratitis samples (Arimoto et al. 2023).

Additionally, luliconazole alters the synthesis of triglycerides and phospholipids, leading to increased levels of toxic hydrogen peroxide within fungal cells. This accumulation of toxic hydrogen peroxide causes damage to intracellular organelles, resulting in cell death (Gupta and Daigle 2016). Notably, the optically active R‐enantiomer of luliconazole has shown greater antifungal activity compared to the racemic mixture (Rani et al. 2024).

### 5.6 Albaconazole

**FIGURE 29:**
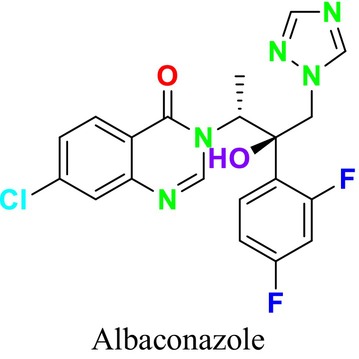
Albaconazole chemical structure.

Albaconazole (UR‐9825), developed by Palau Pharma SA, is a novel triazole antifungal agent characterized by broad‐spectrum activity, favorable pharmacokinetics, and excellent oral bioavailability (Figure 29). Albaconazole has successfully completed various clinical trials, demonstrating efficacy in treating fungal infections caused by pathogens such as *Candida* spp., tinea pedis, and onychomycosis (Ni et al. 2024). This antifungal is particularly noteworthy for its high success rates in treating onychomycosis and its favorable tolerability profile. In a Phase II study, albaconazole was shown to offer high cure rates, positioning it as a promising alternative to existing treatments like terbinafine and itraconazole (Teixeira et al. 2022). Its in vitro activity against *Scedosporium prolificans* and *Paecilomyces* species further distinguishes it from other new triazoles (Chang and Slavin 2017).

### 5.7 Efinaconazole

**FIGURE 30:**
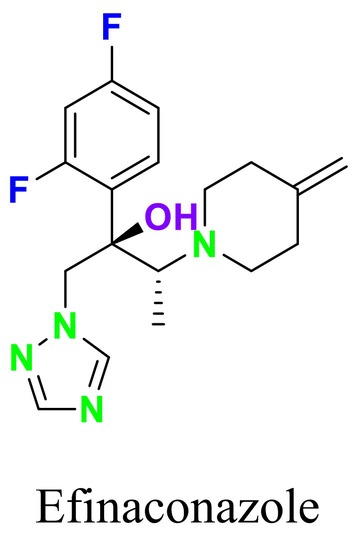
Eficonazole chemical structure.

Efinaconazole (Jublia), developed by Valeant, is an oral triazole antifungal agent with significant pharmacological properties (Figure 30). Approved by the FDA in 2014, efinaconazole has gained widespread clinical use (Ju et al. 2023). It is a topical medication primarily used for the treatment of onychomycosis infections caused by dermatophyte fungi (Mohan and Rudroju 2024). The drug is formulated as a nail lacquer, which facilitates direct application to the affected area, allowing for localized treatment of toenail fungal infections.

Efinaconazole exerts its antifungal effects by penetrating the nail plate and inhibiting CYP51 in the ERG biosynthesis pathway, thereby exhibiting strong antifungal activity. Additionally, it is highly effective against molds beyond dermatophytes and *Candida* species. Clinical trials have yielded promising results, indicating that the drug can be applied once daily for several weeks under the guidance of a healthcare provider (Khan et al. 2024).

In a 2024 study by Ishii and Ohata, the sensitivity of the *Trichophyton rubrum* fungus to the azole antifungal efinaconazole was investigated. The addition of efinaconazole was found to increase the mRNA expression of the Cyp51A isozyme. Through genetic modifications, researchers were able to reduce the resistance response of Cyp51A to azole drugs, thereby lowering the MICs (Ishii, Yamada, and Ohata 2024).

### 5.8 Ravuconazole (Fosravuconazole)

**FIGURE 31:**
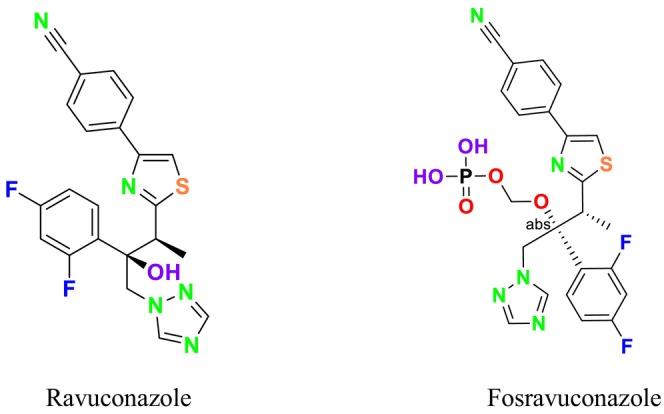
Ravuconazole and fosravuconazole chemical structures.

Ravuconazole is a second‐generation triazole antifungal agent with a broad spectrum of antifungal activity (Figure 31). This drug exhibits potent activity against major pathogenic fungi such as *A. fumigatus* , *C. neoformans* , various *Candida* species, and dermatophytes. Clinical studies have reported a long half‐life of approximately 5–7 days, which makes ravuconazole a promising antifungal agent for the treatment of various invasive fungal diseases (Gazzinelli, Brêtas, and César 2022).

Ravuconazole is currently being evaluated in Phase II clinical trials for its efficacy and safety in treating fungal infections caused by pathogens such as onychomycosis and eumycetoma. These trials have demonstrated that ravuconazole is well tolerated and has high oral bioavailability, establishing its potential as a viable option for new antifungal therapies (Crasto 2024).

Fosravuconazole, a prodrug of ravuconazole, is an antifungal of the triazole class used in the treatment of fungal infections. Formulated as hard gelatin capsules for oral administration, fosravuconazole is converted to ravuconazole in the body, where it inhibits the CYP51 enzyme, disrupting the fungal cell membrane structure. Phase I and II studies have shown that both ravuconazole and its prodrug fosravuconazole l‐lysine ethanolate exhibit significant pharmacokinetic properties and are well tolerated (GlobalData 2024).

Fosravuconazole is available in both intravenous and oral formulations, and following oral administration, it can achieve serum concentrations higher than the MIC90 for *Madurella mycetomatis* (Figure 31; Chandler, Bonifaz, and van de Sande 2023). Additionally, fosravuconazole has been licensed to the Drugs for Neglected Diseases Initiative for the treatment of Chagas disease in Latin America and the Caribbean (Crasto 2014).

### 5.9 Aureobasidin A

**FIGURE 32:**
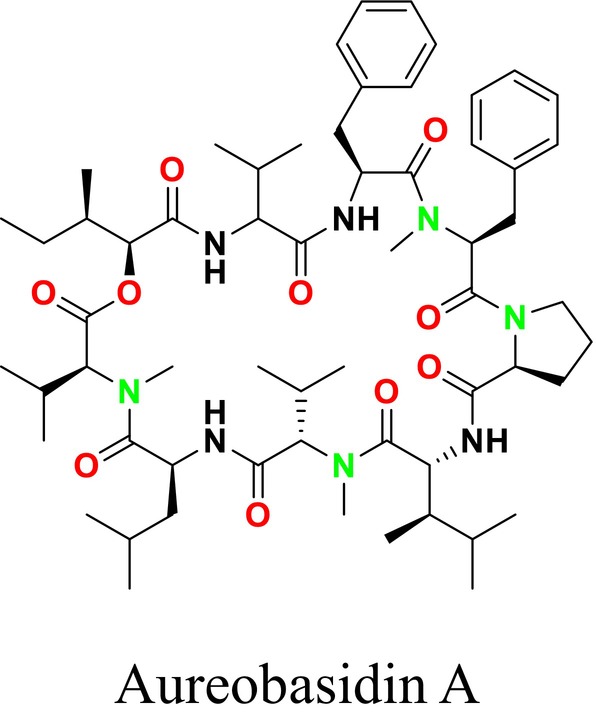
Aureobasidin A chemical structure.

Aureobasidin A is an antifungal agent developed by Takara Bio (Shiga, Japan; Figure 32). Initially discovered in 1989, the licensing patent for aureobasidin A was granted to AureoGen Biosciences/Merck & Co. in 2007 for further research and development (Rauseo et al. 2020). Aureobasidin A inhibits inositol phosphorylceramide synthase, an enzyme expressed by the AUR1 gene in yeast. This enzyme plays a crucial role in the synthesis of sphingolipids in fungal cell membranes, and the AUR1‐C gene is used as an indicator gene for protein interactions (Takara Bio 2024).

Aureobasidin A is a natural cyclic depsipeptide isolated from the fungus *Aureobasidium pullulans*. Structurally, aureobasidin A consists of eight α‐amino acid units and one hydroxy acid unit, which include 2(R)‐hydroxy‐3(R)‐methylpentanoic acid, beta‐hydroxy‐N‐methyl‐L‐valine, N‐methyl‐L‐valine, L‐proline, allo‐L‐isoleucine, N‐methyl‐L‐phenylalanine, L‐leucine, and L‐phenylalanine (Bouz and Doležal 2021). A cyclic depsipeptide is a type of peptide that forms a cyclic structure (Gogineni and Hamann 1862).

Resistance mechanisms to aureobasidin A have been investigated in *S. cerevisiae* . It has been reported that mutations in the AUR1 gene or overexpression of this gene can confer resistance to aureobasidin A. Additionally, the overexpression of *PDR16* reduces the effectiveness of aureobasidin A on the *Ipc1* gene, leading to resistance. The deletion of other genes encoding enzymes that metabolize sphingolipids can also cause resistance to aureobasidin A. Furthermore, aureobasidin A is known to be a substrate for ATP‐binding cassette (ABC) transporters in *S. cerevisiae* , and overexpression of *YOR1*, which encodes a plasma membrane ABC transporter, provides resistance to aureobasidin A. However, more research is needed to understand the resistance mechanisms to aureobasidin A in pathogenic fungi (Zheng et al. 2023).

### 5.10 Amphotericin B Cochleates (CAmB)

AmB is considered the first‐line treatment for severe systemic fungal infections. Due to its poor water solubility and low membrane permeability, the parenteral formulation of AmB is the most used method in clinical practice (Groll et al. 2019). Intravenous formulations, including Fungizone, Abelcet, Ambisome, and Amphocil, have been marketed for decades. However, the toxicity and costs associated with lipid formulations of AmB have limited their widespread use in hospitals (Suberviola 2021).

**FIGURE 33:**
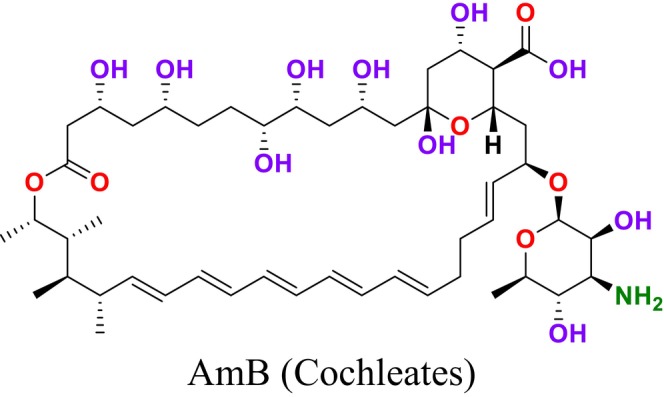
CAmB chemical structure.

CAmB is a new oral formulation of AmB derived from the polyene class (Figure 33). Developed by Matinas BioPharma, this drug is currently in Phase II clinical trials. CAmBs are formulated using phosphatidylserine with phospholipid‐calcium, making them stable in the gastrointestinal tract. This formulation has been reported to successfully treat *C. albicans* infection in a mouse model (Santangelo et al. 2000).

Cochleates are composed of a negatively charged lipid, such as phosphatidylserine, and a divalent cation, such as calcium. This structure acts as a binding agent to encapsulate hydrophobic and amphiphilic molecules. The interaction of cations with phospholipids rearranges the phospholipid bilayers to form spiral structures without an inner aqueous phase. CAmB is an antifungal drug produced using this approach (Hamill 2013; Aigner and Lass‐Flörl 2020).

Infusion‐related reactions and dose‐dependent nephrotoxicity limit the use of AmB deoxycholate (Hamill 2013). Unlike currently approved polyene formulations, CAmB is sensitive to degradation by the gastrointestinal system. Cochleates consist of phosphatidylserine, which are phospholipid–calcium precipitates. Their multilayered structures form solid, lipid bilayers arranged in spirals without an aqueous inner cavity (Santangelo et al. 2000).

Following oral administration, cochleates are absorbed from the gastrointestinal tract and enter the circulation. When calcium concentrations in cochleates decrease, the spiral formation opens, releasing the encapsulated drug into the cell. Various formulations using cochleates have been developed to deliver substances such as proteins, peptides, and anticancer drugs like raloxifene, fisetin, and doxorubicin. Limitations of cochleate formulations include loss of stability at temperatures above 4°C, precipitation during storage, and production costs (Shende, Khair, and Gaud 2019).

### 5.11 Ibrexafungerp

Ibrexafungerp is an antifungal drug with high bioavailability that can be administered orally or intravenously (Davis, Donnelley, and Thompson 2020). When taken with food, the drug's dissolution in the stomach and systemic absorption increases. Phase III studies of oral ibrexafungerp utilized an initial loading dose of 750 mg PO BID for the first 2 days, followed by 750 mg PO daily for subsequent doses. Earlier Phase II studies used a loading dose of 1250 mg PO once. It was well tolerated in these studies, with side effects primarily limited to gastrointestinal symptoms, and no significant differences were observed compared to placebo in several studies (Spec et al. 2019).

**FIGURE 34:**
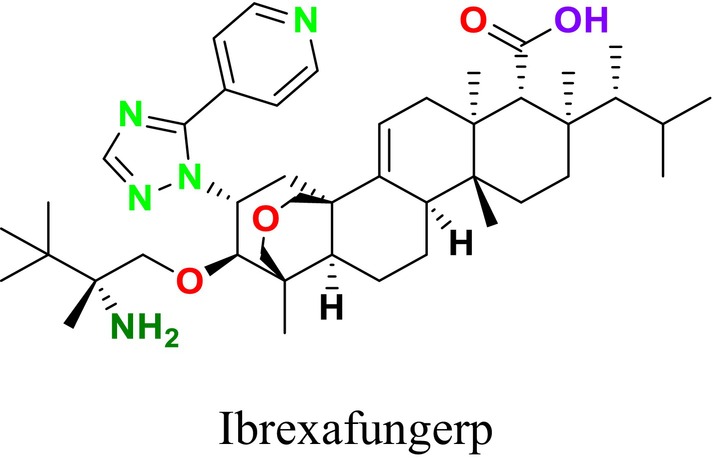
Ibrexafungerp chemical structure.

Ibrexafungerp (SCY‐078) is a novel oral glucan synthase inhibitor currently under clinical investigation for the treatment of candidiasis (Figure 34; Mesquida et al. 2022). Like echinocandins, its spectrum of activity includes *Candida* spp., such as *C. glabrata* and *C. auris* (Davis, Donnelley, and Thompson 2020). Despite similar mechanisms of action, ibrexafungerp maintains in vitro activity against echinocandin‐resistant *Candida* strains, suggesting a difference in target site avidity (the strength of antibody binding to an antigen). Additionally, in vitro studies have shown fungistatic activity against *Aspergillus* spp., including azole‐resistant strains (Nunnally et al. 2019).

Vulvovaginal candidiasis (VVC) is a global issue and the second most common cause of vaginitis in women. Current treatment options for VVC include oral and topical azoles with fungistatic effects. One of the reasons for the development of ibrexafungerp is to offer an alternative oral treatment option for VVC. Ibrexafungerp received FDA approval in June 2021 for the treatment of VVC and was later approved in December 2022 for the treatment of recurrent VVC.

Like echinocandin antifungals, ibrexafungerp inhibits (1,3)‐β‐D‐glucan, a critical component of the fungal cell wall (Phillips, Rocktashel, and Merjanian 2023). This inhibition leads to a reduction in (1,3)‐β‐D‐glucan polymers, weakening the fungal cell wall and ultimately causing cell lysis and death (Nyirjesy et al. 2022). Ibrexafungerp is also a semi‐synthetic derivative of enfumafungin, a triterpene glycoside, and represents a novel antifungal inhibitor (Lu et al. 2023). Enfumafungin is a glycosylated fernene‐type triterpenoid produced by the fungus *Hormoconis resinae* (Kuhnert et al. 2018).

The Infectious Diseases Society of America (IDSA) recommends echinocandins, lipid formulation AmB, or voriconazole as treatment options for *C. krusei* (Pappas et al. 2015). *C. krusei* is inherently resistant to fluconazole, which may reduce susceptibility to AmB (Abbas et al. 2000; Schilling et al. 2008). It is generally susceptible to echinocandins and may exhibit various susceptibility patterns to voriconazole (Overgaauw et al. 2020). Recent studies have shown that ibrexafungerp exhibits in vitro activity against different *Candida* species, including *C. krusei* (Alghamdi et al. 2024).

### 5.12 Rezafungin

**FIGURE 35:**
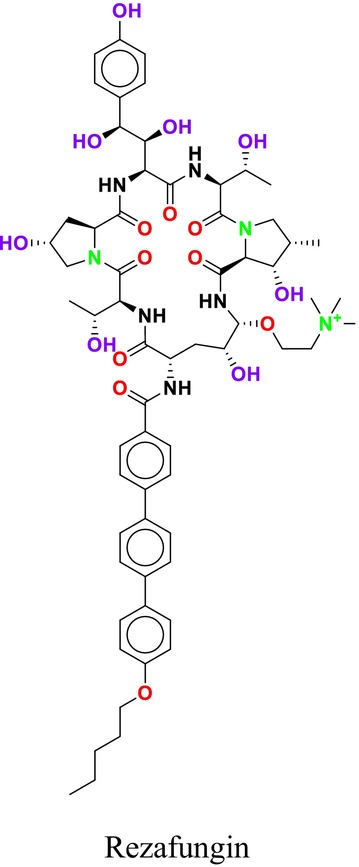
Rezafungin chemical structure.

Rezafungin is a novel, semi‐synthetic echinocandin antifungal agent administered intravenously, characterized by its extended half‐life (Figure 35). Derived from a fermentation product of *A. nidulans* , rezafungin inhibits the 1,3‐β‐D‐glucan synthase enzyme complex, exerting fungicidal effects against *Candida* species and fungistatic effects against *Aspergillus* species, similar to other echinocandins (Adeel et al. 2021). Structural modifications have made rezafungin a more stable and soluble analog of anidulafungin, significantly enhancing its tissue distribution and resulting in a substantially increased half‐life (~133 h) compared to other echinocandins, offering notable pharmacokinetic advantages (Sandison et al. 2017).

Recent Phase II clinical trials compared rezafungin to caspofungin for the treatment of candidemia and invasive candidiasis. Rezafungin, administered at a loading dose of 400 mg in the first week followed by 200 mg once weekly, emerged as the most effective dosing regimen and is currently under investigation in Phase III trials (Van Daele et al. 2019; Gangneux et al. 2019). Moreover, rezafungin has been granted orphan drug designation by the FDA for the treatment of vulvovaginal candidiasis and has shown efficacy against *Candida* spp., *Aspergillus* spp., and *Pneumocystis* spp. (Gintjee, Donnelley, and Thompson 2020; Van Daele et al. 2019).

The spectrum of activity of rezafungin encompasses numerous clinically significant fungal species, including *C. albicans* , *C. krusei* , and *C. tropicalis* , in addition to various *Aspergillus* species. The impact of FKS mutations, which confer echinocandin resistance, on rezafungin MIC values has been reported, although not consistently across all isolates. Furthermore, cross‐resistance between rezafungin, caspofungin, and anidulafungin has been observed; however, the front‐loading dosing regimen used in studies is suggested to mitigate the development of resistance (Zhao et al. 2017). In vitro studies have also demonstrated that rezafungin exhibits stronger activity against *C. auris* than both caspofungin and micafungin (Lepak, Zhao, and Andesa 2018).

### 5.13 Fosmanogepix

**FIGURE 36:**
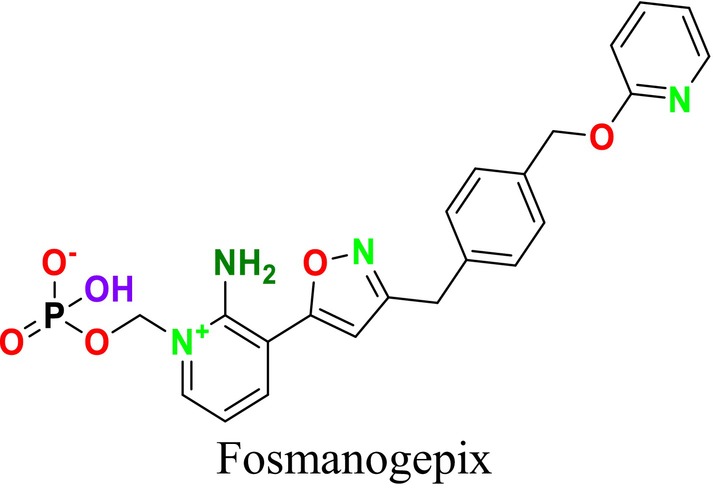
Fosmanogepix chemical structure.

Fosmanogepix, developed by Amplyx Pharmaceuticals, is an antifungal inhibitor targeting the enzyme Gwt1, which is responsible for the biosynthesis of glycosylphosphatidylinositol (GPI) and GPI anchors (Figure 36; Alkhazraji et al. 2020; Shaw and Ibrahim 2020). The Gwt1 enzyme plays a crucial role in the GPI anchor biosynthesis pathway and is involved in the localization of over 60 mannoproteins across fungal cell structures (August and Kale‐Pradhan 2024). Clinically, fosmanogepix is administered as an N‐phosphonooxymethyl prodrug, which is rapidly converted into manogepix by host phosphatases. It is currently undergoing Phase III clinical trials (Wiederhold 2022). Fosmanogepix has been reported to inhibit the growth of yeasts, molds, *Candida* spp., *Cryptococcus* spp., *Coccidioides* spp., and *Aspergillus* spp. (McCarthy et al. 2017).

Fosmanogepix (PF‐07842805; APX001; E1211) is the first member of the “manogepix” class of antifungal drugs, characterized by a unique mechanism of action (Miyazaki et al. 2011; Watanabe et al. 2012). In fungi, GPI mediates the cross‐linking of cell wall mannoproteins to β‐1,6‐glucan. Inhibition of Gwt1 results in pleiotropic effects on fungal cells, including inhibition of adhesion to surfaces, biofilm formation, and germ tube formation, leading to severe growth defects and cell death (Watanabe et al. 2012; McLellan et al. 2012). Manogepix does not inhibit PIG‐W, a mammalian orthologue that also catalyzes the inositol acylation of GPI but shares low homology with Gwt1 (Murakami et al. 2003). The pleiotropic mechanism of manogepix, where a single biochemical target binding induces multiple biological effects, has been shown to result in broad‐spectrum activity against a range of clinically significant fungi and molds, including resistant strains (Shaw and Ibrahim 2020).

However, fosmanogepix exhibits weak in vitro activity against *C. krusei* (MIC range: 2 to > 32 mg/L). This reduced efficacy is likely due to nontarget‐based resistance mechanisms, such as differences in cell permeability of *C. krusei* , rather than target‐based expression of the Gwt1 protein (Miyazaki et al. 2011; Kapoor et al. 2020).

### 5.14 Nikkomisin Z

**FIGURE 37:**
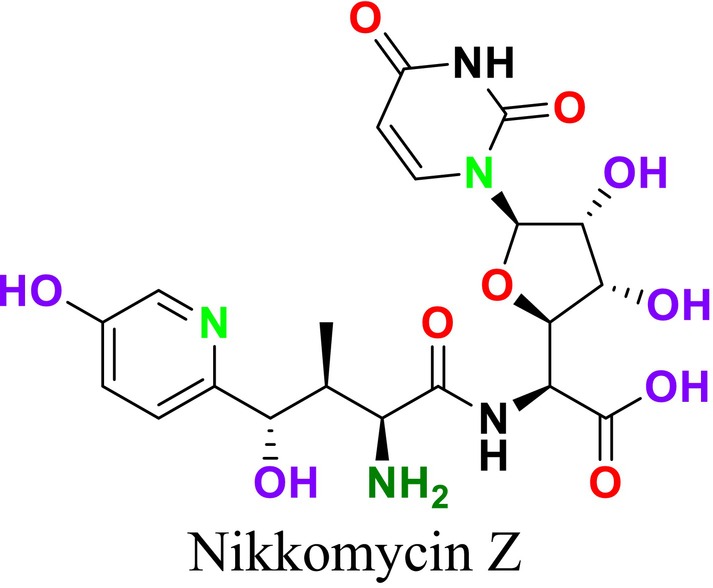
Nikkomycin Z chemical structure.

Nikkomycin Z, derived from *Streptomyces tendae* , is a peptidyl nucleoside antifungal drug candidate with a novel mechanism of action that targets chitin synthesis in the fungal cell wall (Figure 37; Adnan et al. 2023). Chitin is a critical component of the fungal cell wall, and Nikkomycin Z competitively inhibits the enzyme responsible for its synthesis due to its structural similarity to UDP‐N‐acetylglucosamine. By inhibiting this enzyme, Nikkomycin Z disrupts the synthesis of the fungal cell wall, thereby hindering fungal growth.

Nikkomycin Z is particularly effective against dimorphic fungi, which can transition between two different morphological forms depending on environmental conditions. It also holds potential for use against species such as *Coccidioides*, *C. albicans* , and *A. fumigatus* (Sass et al. 2021). Additionally, in vitro studies have demonstrated significant activity against *Sporothrix* species, making nikkomycin Z a promising candidate for the treatment of fungal infections (Poester et al. 2023).

As of 2024, Phase II clinical trials for Nikkomycin Z have commenced, focusing on assessing its efficacy and safety in patients with severe fungal infections. Current studies indicate that nikkomycin Z exhibits synergistic effects when used in combination with other antifungal drugs, significantly enhancing therapeutic efficacy. Furthermore, in vitro animal models have shown that Nikkomycin Z achieves a 100% survival rate in cases of severe fungal infections and completely suppresses brain infections (Valley Fever Solutions 2024).

### 5.15 VL‐2397

**FIGURE 38:**
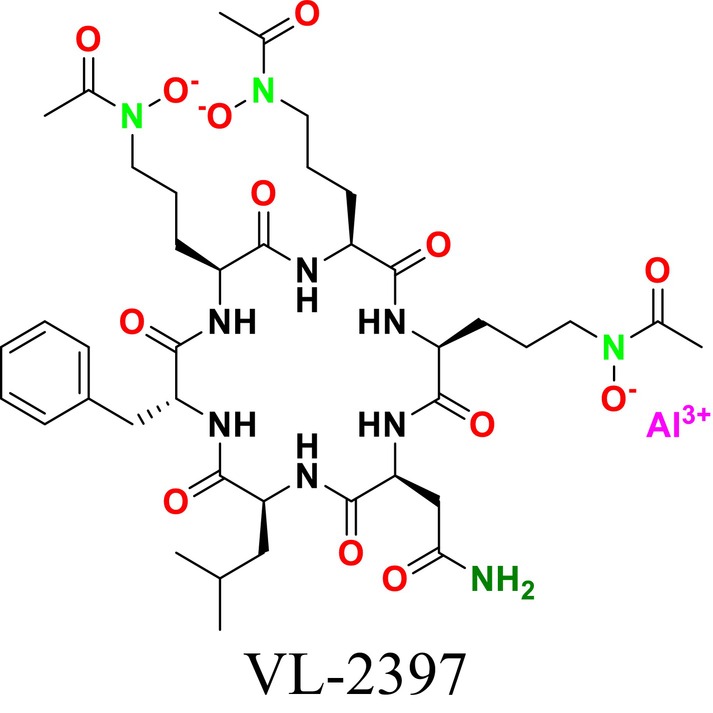
VL‐2397 chemical structure.

VL‐2397 is an antifungal drug developed by Vical Pharmaceuticals. Fungi uptake this drug through the siderophore iron transporter 1 (Sit1), leading to the disruption of intracellular processes (Figure 38). Currently, VL‐2397 is in Phase II clinical trials and has been reported to be highly effective against *A. fumigatus* (Van Daele et al. 2019; Kovanda et al. 2019; Dietl et al. 2019). Sit1 has been shown to mediate the uptake of VL‐2397 (ASP2397), a novel antifungal drug with a ferrichrome‐type structure. Additionally, research is ongoing to investigate the role of the fifth potential siderophore transporter, MirC—a specific siderophore transporter protein found in fungi such as *A. fumigatus* —in the biosynthesis of fusarin C, a mycotoxin produced by *Fusarium moniliforme* and *Fusarium venenatum* (Happacher et al. 2023).

VL‐2397 is derived from *Acremonium persicinum* and has demonstrated protective effects against lethal outcomes following *A. fumigatus* infection in both a silkworm larva model and a murine model of invasive pulmonary aspergillosis (Nakamura et al. 2017). Unlike existing classes of antifungal drugs that target fungal cell wall or plasma membrane components, VL‐2397 operates via a novel antifungal mechanism involving a cyclic hexapeptide structure that contains aluminum instead of iron (Nakamura et al. 2019). Siderophore iron transporters are utilized by various fungi to transport iron‐bound siderophores from the environment into fungal cells, a process critical for the growth and survival of *A. fumigatus* and other fungal pathogens (Haas 2012, 2014; Schrettl et al. 2004).

Mammalian cells lack Sit1, which means VL‐2397, leveraging this mechanism, does not affect human cells and allows for selective uptake by fungal cells, potentially providing a favorable safety profile (Hsiang and Baillie 2005). Recent studies have indicated that the Sit1 protein plays a significant role in the uptake of this antifungal agent by fungal cells, although the antifungal activity does not depend on the uptake of aluminum from the external environment by fungal cells (Dietl et al. 2019).

### 5.16 T‐2307

**FIGURE 39:**
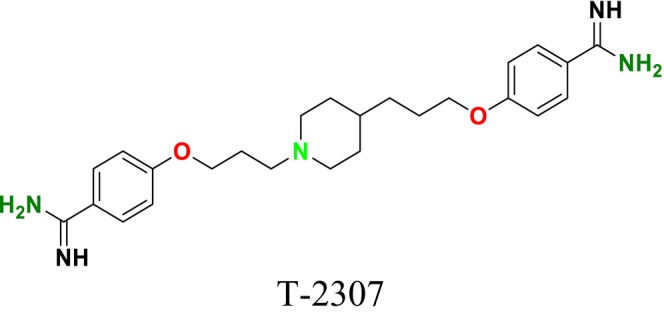
T‐2307 chemical structure.

T‐2307 is a novel arylamidine antifungal drug structurally similar to pentamidine, which causes the collapse of mitochondrial membrane potential (Figure 39). It exhibits potent activity against *Candida* species, including azole‐resistant isolates, and has been reported to be highly effective against echinocandin‐resistant *C. albicans* (Wiederhold et al. 2016). T‐2307 targets the inhibition of the fungal respiratory chain. Developed by Toyama Chemical Co. Ltd. (Tokyo, Japan), T‐2307 shows broad‐spectrum in vitro and in vivo antifungal activity against most pathogenic fungi. Unlike traditional antifungal drugs used clinically, T‐2307 selectively disrupts fungal mitochondrial function while sparing mammalian cells (Maione et al. 2022).

The uptake of T‐2307 by fungal cells is mediated by a specific polyamine transporter related to spermine and spermidine uptake, resulting in an intracellular concentration in *C. albicans* that is 3000 times higher than that in the extracellular environment. Upon entering the cytosol, a gel‐like fluid found between the cell membrane and the nucleus (Fry 2017), T‐2307 reaches the mitochondria, where it disrupts the mitochondrial membrane potential, leading to mitochondrial dysfunction. Remarkably, T‐2307 has minimal effects on rat liver mitochondrial functions, indicating its selectivity as an inhibitor of fungal mitochondrial function (Yamashita et al. 2019).

Moreover, the MICs of T‐2307 are particularly low for fungi such as *Candida* species, suggesting that despite its low ocular permeability, T‐2307 could still be effective against ocular candidiasis, a known complication of candidemia (Abe et al. 2019; Chen et al. 2024).

### 5.17 MGCD290

MGCD290 is an antifungal agent that inhibits Hos2, an enzyme belonging to the HDAC family of lysine deacetylases. It also disrupts the interaction between the mediator complex and Pdr1 (a transcription factor in *C. glabrata* ) through a small molecule, iKIX1, which is effective against antifungal‐resistant fungal species such as *C. glabrata* (Lu et al. 2023; Jenner 2020). This inhibition leads to decreased expression of Pdr5, an ABC transporter protein in *C. glabrata* . MGCD290 also affects non‐histone proteins such as HSP90 and is administered orally.

Developed by Mirati Therapeutics Inc., MGCD290 has been tested in phase II clinical trials against fungal infections. It is used in combination with azoles and echinocandins, showing fungicidal activity against *Candida* species and *Aspergillus* species (Gintjee, Donnelley, and Thompson 2020). In addition to its intrinsic antifungal activity, MGCD290 has demonstrated synergistic effects with other antifungal drugs in multiple studies. This synergy may enhance the fungicidal effects of agents targeting the fungal cell wall or membrane by disrupting the inhibition of fungal proteins and cellular stress responses. Several in vitro studies have shown that the addition of low concentrations of MGCD290 to *Candida* spp. and *Aspergillus* spp. strains enhances both azole and echinocandin activity, reduces MICs, and leads to categorical shifts from resistant to intermediate or susceptible in numerous cases (Pfaller et al. 2009, 2015).

Despite promising in vitro results, MGCD290 has not yet demonstrated in vivo efficacy (Perfect 2017). A Phase II study evaluating MGCD290 as an adjunct to fluconazole in patients with severe vulvovaginal candidiasis showed no additional benefit compared to fluconazole monotherapy, although it was well tolerated (MethylGene Inc 2013).

### 5.18 Olorofim

**FIGURE 40:**
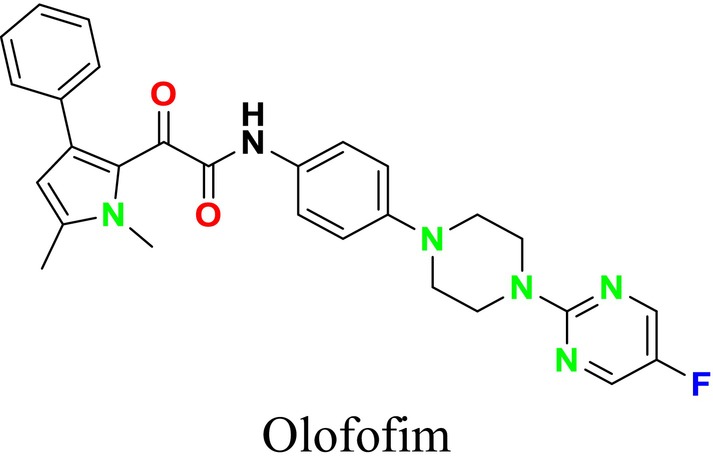
Olorofim chemical structure.

Olorofim is a next‐generation antifungal agent belonging to the orotomide class, currently undergoing Phase IA and Phase III clinical trials for various mold infections, including those caused by azole‐resistant isolates (Figure 40). Orotomides exert their antifungal effect by inhibiting dihydroorotate dehydrogenase, an enzyme essential for pyrimidine synthesis (Oliver et al. 2016). This inhibition disrupts nucleic acid and cell wall synthesis in fungi, representing a novel mechanism of action. Olorofim has shown potential efficacy against fungi such as *Aspergillus* species and *Scedosporium* species (Hoenigl et al. 2021; Rivero‐Menendez, Cuenca‐Estrella, and Alastruey‐Izquierdo 2019).

The inhibition of pyrimidine production adversely affects fungal cell nucleic acid, cell wall, phospholipid synthesis, cell regulation, and protein production. Olorofim demonstrates a time‐dependent antifungal effect and can be administered orally or intravenously, with most studies focusing on the oral formulation. Pharmacokinetic studies have revealed that olorofim distributes to various tissues, including brain tissue, albeit at low levels. It is metabolized by the CYP450 system, particularly CYP3A4, making it susceptible to drug interactions (Van Daele et al. 2019; Tom 2017).

Olorofim exhibits broad‐spectrum mold activity and is particularly effective against *Aspergillus* species. It shows strong activity against common *Aspergillus* species ( *A. fumigatus* , *A. nidulans* , *A. terreus* , and *A. niger* ) as well as species that are difficult to treat and often exhibit intrinsic resistance to many existing antifungal classes ( *A. lentulus* and *A. calidoustus*). Additionally, Olorofim is effective against multidrug‐resistant *Aspergillus* strains, indicating a lack of cross‐resistance due to its novel mechanism of action. It has been observed that exposure to Olorofim does not easily induce resistance in *A. fumigatus* isolates.

Moreover, Olorofim is effective against *Lomentospora prolificans* (which currently has no other effective therapeutic alternative) and *Scedosporium* species. Both in vitro and in vivo studies have demonstrated olorofim's activity against *Coccidioides* and other rare pathogenic fungi (Van Daele et al. 2019; Rivero‐Menendez, Cuenca‐Estrella, and Alastruey‐Izquierdo 2019; Tom 2017; Wiederhold, Najvar, et al. 2018).

## 6 Development of Dual‐Target Antifungal Agents

In recent years, the number of studies investigating the combination of antifungal agents with different mechanisms of action to counteract the development of resistance and enhance antimicrobial efficacy has significantly increased (Huang et al. 2023). The molecular and genetic complexity of diseases and infections often reveals that targeting a single pathway is insufficient to achieve effective and sustained remission. Recent drug discovery research has primarily focused on developing drug combinations targeting multiple signaling pathways or single compounds capable of inhibiting multiple pathways. This approach has transformed the traditional design of single‐target drugs into the concept of “polypharmacology” or “multi‐target” drug discovery, aiming to develop a single drug that simultaneously targets multiple biological systems (Pinzi et al. 2020). The increasing incidence of hepatotoxicity, nephrotoxicity, and the emergence of resistant fungal strains have made this approach even more compelling. Combination or dual‐target drugs can synergistically affect enzymes targeted by conventional single‐target inhibitors, creating a clinical therapeutic effect that can be described as “1 + 1 > 2” (Sun, Liu, et al. 2023).

Zhou and colleagues have developed dual‐target antifungal compounds to address the problem of resistance to clinically used antifungal drugs. These compounds simultaneously target both the fungal cell membrane and DNA. Specifically, poly(2‐oxazoline) compounds containing DNA‐binding naphthalene groups that mimic host defense peptides, such as (Gly 0.8Nap0.2)_20_, have demonstrated high antifungal selectivity against drug‐resistant fungal pathogens, including *Candida* spp., *C. neoformans* , *C. gattii*, and *A. fumigatus* . In vivo experiments have shown that these compounds are effective in various infection models in mice, including skin abrasions, corneal infections, and systemic infections, with minimal side effects and high biocompatibility.

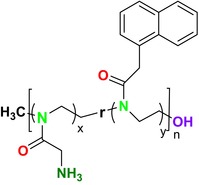

$${\left(\mathrm{Gly}x\mathrm{Nap}y\right)}_{20}\;\left(x+y=1,y=10\%–50\%\right).$$

The compound (Gly 0.8Nap0.2)_20_ has demonstrated effectiveness against multidrug‐resistant fungal pathogens, making it a promising candidate for future antifungal therapy (Zhou et al. 2024).

This dual‐target approach represents a promising strategy in the fight against fungal resistance, providing a potential avenue for developing new antifungal therapies that can circumvent the limitations of current single‐target agents. As the need for effective antifungal treatments continues to grow, especially in the face of rising drug resistance, these innovative approaches may play a crucial role in enhancing the efficacy and safety of antifungal therapy.

In recent years, dual‐targeted antifungal studies have gained great importance in preventing the development of resistance against fungal infections and increasing treatment effectiveness. These studies aim to expand the scope of antifungal treatments by blocking two different biological targets simultaneously. This makes dual‐targeted antifungal drug development studies important. Targets such as cyclooxygenase (COX) and SE are associated with oxireductase enzymes, while the Pd‐L1 target interacts with B7 proteins that play an immune regulatory role. Epigenetic regulators such as HDAC, Bromodomain and Extra‐Terminal (BET) and Bromodomain‐Containing Protein 4 (BRD4) play a critical role in the control of gene expression and aim to suppress BET response, studies carried out on this target aim to reduce the resistance of fungi to environmental stresses. Furthermore, tyrosine kinases belonging to the Januse Kinase 2 (JAK2) family play an important role in cell signaling pathways, and studies on these kinases aim to disrupt fungal cellular communication systems. These studies enable more effective antifungal drug development strategies in the future (Table 5).

**TABLE 5:** Studies on dual‐action antifungal drugs.

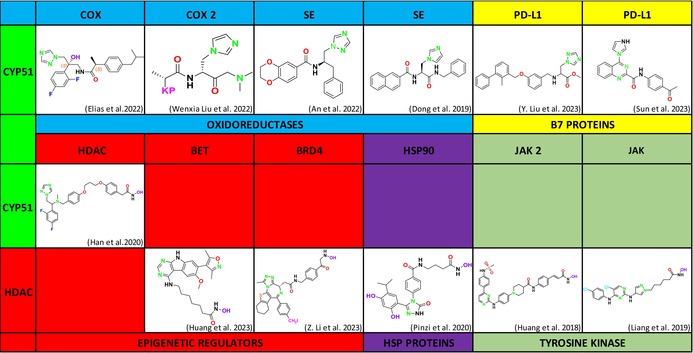

Dual‐target combinations such as CYP51‐COX, CYP51‐HDAC, and SE‐CYP51 act by disrupting the life cycle of fungi at both the cell membrane and epigenetic level. Studies against targets involved in immune regulation, such as programmed death‐ligand 1 (PD‐L1) and B7 proteins, as well as epigenetic regulators, such as HDAC, BRD4, and HSP90, inhibit the adaptation of fungi to environmental stress. Combinations of JAK2‐HDAC and BET‐HDAC provide a more comprehensive antifungal effect by targeting tyrosine kinase and epigenetic pathways. These strategies both prevent the development of resistance and increase treatment effectiveness.

### 6.1 CYP51‐Cyclooxygenase (COX)

**FIGURE 41:**
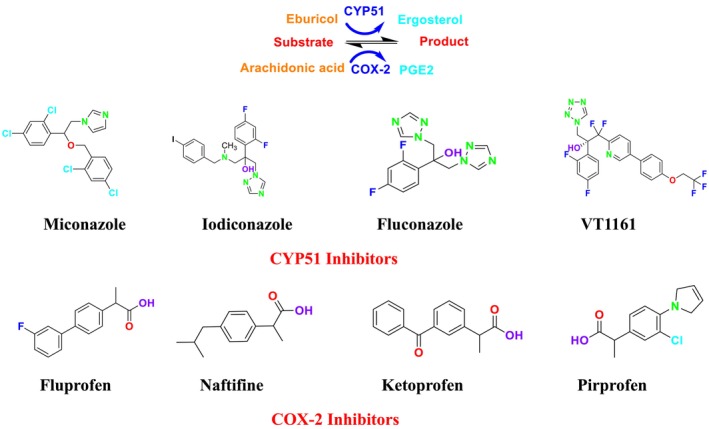
CYP51 and COX2 dual inhibitors (Liu et al. 2022).

One of the studies aiming to develop a new class of antifungal agents that enhance antifungal efficacy and reduce tolerance by combining the pharmacophores of CYP51 and COX inhibitors was conducted by Elias and colleagues in 2022 (Figure 41; Elias, Basu, and Fridman 2022). In this study, enantiomerically pure azole pharmacophores were linked to different COX inhibitors to synthesize 24 chiral hybrid compound derivatives. The antifungal activities of these compounds against *Candida* species and the effects of chirality on their potential and tolerance were reported. Additionally, regarding the dual mode of action, it was demonstrated that these hybrids predominantly inhibit CYP51, the target of azole drugs, and possess a secondary mode of action contributed by the COX inhibitor segment. It was also determined that these hybrids are active in a mutant lacking CYP51 and exhibit lower tolerance levels than fluconazole and voriconazole. By examining the structure–activity relationships of the compounds, it was shown that compound **1** is particularly active and that the absolute configuration of the chiral center on the benzylic carbon of the azole pharmacophore segment has a decisive effect on antifungal activity in both diastereomeric tetrads and enantiomeric pairs. However, hybrids with the S‐configuration on the benzylic carbon were found to have higher antifungal potential compared to those with the R‐configuration. The findings also highlighted the structural differences in antifungal activity between ibuprofen‐ and flurbiprofen‐based tetrads and niflumic acid‐ and diflunisal‐based enantiomeric pairs as COX inhibitors. These findings provide significant insights into how the antifungal activity of azole derivatives can be correlated with molecular‐level structural features, offering important guidance in the design and development of such compounds. Additionally, the study noted that by examining the tolerance levels of the hybrids, the synthesized hybrids exhibited lower tolerance levels compared to FLC and VOR, which could provide a significant advantage in reducing the persistence and recurrence of infection.

**FIGURE 42:**
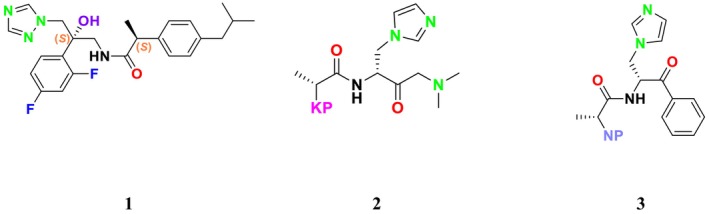
Compounds 1, 2 and 3 chemical structures.

In a study by Wenxia Liu and colleagues (2022), focusing on determining the relationship between fungal infections and inflammation, it was suggested that the synergistic function between azole antifungal agents (CYP51 inhibitors) and nonsteroidal anti‐ınflammatory drugs (NSAIDs; COX‐2 inhibitors) could effectively enhance their therapeutic efficacy in vivo (Figure 41; Liu et al. 2022). In this context, the binding models of COX‐2 and CYP51 inhibitors were analyzed for the design of new dual‐target inhibitors aiming at COX‐2 and CYP51. Through scaffold screening and combination, three new series of compounds were designed and synthesized. Among these compounds, **2** and **3** were found to inhibit CYP51, blocking the ERG synthesis pathway, reducing the fluidity and permeability of the fungal cell membrane, leading to the disintegration of fungal cells, accumulation of reactive oxygen species (ROS), and mitochondrial damage, ultimately causing apoptosis (Figure 42). On the other hand, compounds **2** and **3** were also found to inhibit COX‐2 activity, reducing the expression of enzymes that induce inflammation. Additionally, these compounds exhibited immunomodulatory effects by increasing CD3 and CD8 T‐cell levels.

These studies offer a new path and strategy for developing dual‐acting hybrid antifungal drugs that contain both azole antifungals and COX inhibitors. These hybrid structures are anticipated to potentially provide more effective treatments due to their potent antifungal activity and low tolerance levels. These findings are considered a promising potential avenue for advancing antifungal drug development.

### 6.2 CYP51‐Histone Deasetilase (HDAC)

**FIGURE 43:**
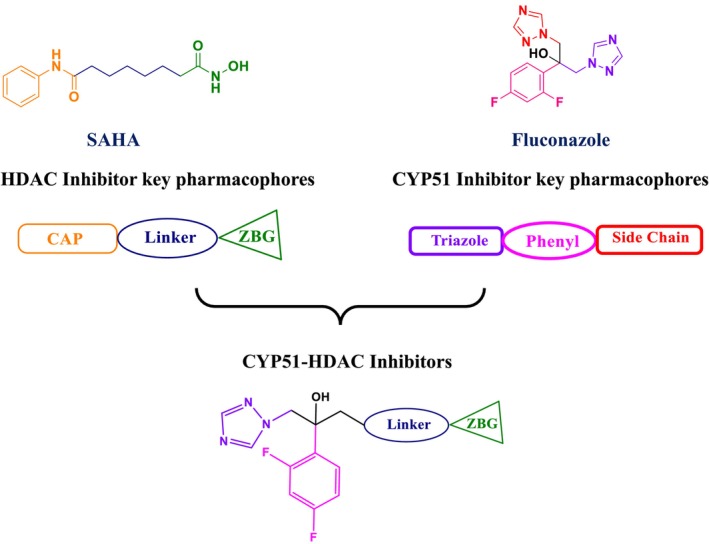
Design of CYP51/HDAC dual inhibitors via pharmacophore fusion (Han et al. 2020).

CYP51‐HDAC dual inhibitors are suggested to be a promising strategy for developing new antifungal agents against azole‐resistant clinical isolates. The design and synthesis of the first‐generation CYP51‐HDAC dual inhibitors for the treatment of azole‐resistant *Candida* species were carried out through pharmacophore fusion by Han and colleagues in 2020 (Figure 43; Han et al. 2020).

A significant portion of the compounds exhibited strong inhibitory activities against resistant fungi in vitro. Notably, compounds **4** and **5** were found to have particularly potent effects for treating resistant strains both in vitro and in vivo. In murine models of azole‐resistant strains, these compounds significantly reduced fungal kidney burden and extended survival time.

**FIGURE 44:**
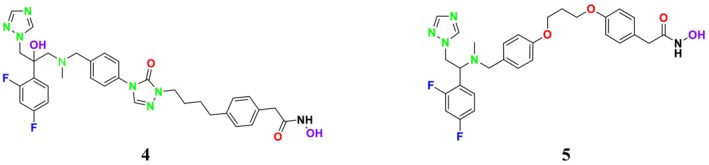
Compounds 4 and 5 chemical structures.

Mechanism studies of antifungal activity for compounds **4** and **5** revealed that they demonstrated antifungal activity by suppressing ERG biosynthesis (CYP51 inhibition) and exhibited anti‐resistant profiles by downregulating the expression of CYP51 and the efflux pump (HDAC inhibition), as well as effectively inhibiting biofilm formation (Figure 44).

The findings suggest that CYP51‐HDAC dual inhibitors could provide an effective strategy for the treatment of azole‐resistant candidiasis. Further investigation into the antifungal mechanisms and structural optimization of dual CYP51/HDAC inhibitors is currently in progress.

### 6.3 SE‐CYP51

It is well known that ERG plays a vital role in maintaining fungal cell membrane fluidity and osmotic pressure. An and colleagues conducted a study evaluating the synergistic effects of key enzymes in ERG biosynthesis, such as SE and CYP51 (An et al. 2022). In this context, a study was conducted to discover new antifungal compounds with dual‐target (SE/CYP51) inhibitory activity. Fragment‐based drug discovery (FBDD) was employed to develop three benzodioxane compounds by screening potent fragments across various species ( *C. albicans* 17#, CaR, 103, 901) based on dual‐target characteristics.

**FIGURE 45:**
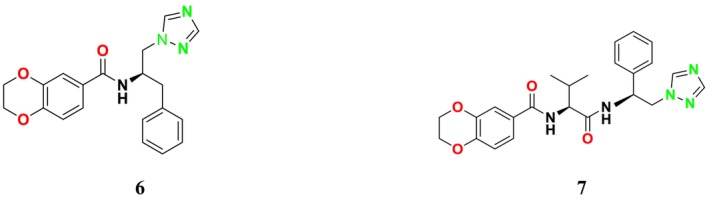
Compounds 6 and 7 chemical structures.

After the synthesis of the designed compounds, their potent activities against pathogenic fungal strains were determined. Notably, compounds **6** and **7** were found to exhibit broad‐spectrum antifungal activity and notable activity against drug‐resistant strains (Figure 45). Mechanistic studies confirmed that these compounds effectively inhibited SE and CYP51 targets, blocking the ERG biosynthesis pathway, which could lead to the death of fungal cells. Further studies revealed that compounds **6** and **7** also maintained their antifungal activities in vivo.

**FIGURE 46:**
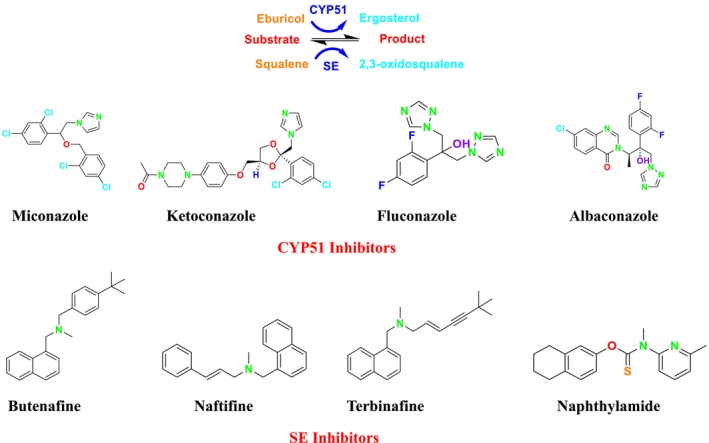
CYP51 and SE dual inhibitors (An et al. 2022).

This study not only presented a novel dual‐target drug design strategy and methodology but also contributed to the discovery of potential antifungal compounds. While the crystal structure of *C. albicans* CYP51 and the homology model of *C. albicans* SE had been previously reported (An et al. 2022), it was observed that dual‐target drug design could not prevent reduced drug‐binding capacity due to mutations in the receptor protein. This limitation hindered the expected synergistic inhibitory effect of dual‐target compounds on drug‐resistant fungi. To address this issue, a CYP51 mutation model was developed and used as a guide in designing new dual‐target (SE/CYP51) antifungal inhibitors (Figure 46). It was also noted that dual‐target or multi‐target drug design involves challenges due to the increased spatial complexity of target enzymes. Conventional methods may struggle to flexibly cover this complexity, which is why the FBDD approach was recommended. In the FBDD method, core fragment structures that can adapt to different active sites of the target enzyme are used by flexibly handling the molecular structure. The study analyzed the structural characteristics of antifungal inhibitors, extracting core groups and using them as a source for subsequent fragment screening. The spatial distribution and structural characteristics of ligand molecules were examined in the identified active sites, and the binding regions of SE and CYP51 inhibitors (e.g., naftifine and VT1161) in the relevant active sites were identified. This study presented new strategies to overcome challenges in dual‐target drug design and made significant progress toward developing potential antifungal compounds.

In a study conducted by Dong and colleagues in 2019, it was stated that fungal infections and drug resistance have increased with environmental changes, and SE and CYP51 have emerged as important antifungal targets (Dong et al. 2019). The relevant pharmacophore models were used to guide the development of new inhibitors. A common feature pharmacophore model for the SE inhibitor and a structure‐based pharmacophore model for the CYP51 receptor were constructed. Selected suitable organic fragments were overlapped onto these pharmacophore features to design and synthesize the compounds. Notably, compound 8 attracted attention due to its ability to match the features of both SE and CYP51 pharmacophores.

**FIGURE 47:**
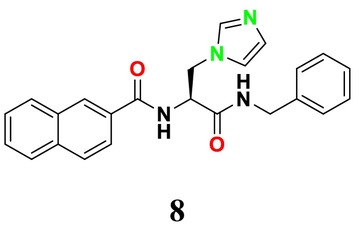
Compound **8** chemical structure.

Analyses revealed that these compounds exhibited strong antifungal activity. Mechanism studies confirmed that these compounds could exert inhibitory effects on the relevant target enzymes. It was stated that compound **8** could block the ERG synthesis pathway through dual‐target inhibition (Figure 47; SE and CYP51). This study highlights the importance and effectiveness of pharmacophore modeling in the design and discovery of new antifungal inhibitors. Pharmacophore models can provide critical guidance in the development of potential antifungal agents, forming a significant step toward the discovery of effective new treatments against fungal infections (Dong et al. 2019). These findings demonstrate that pharmacophore models developed for SE and CYP51 can guide the design and discovery of new antifungal inhibitors.

### 6.4 Programmed Death‐Ligand 1 (PD L1) ‐Lanosterol 14‐α Demethylase (CYP51)

**FIGURE 48:**
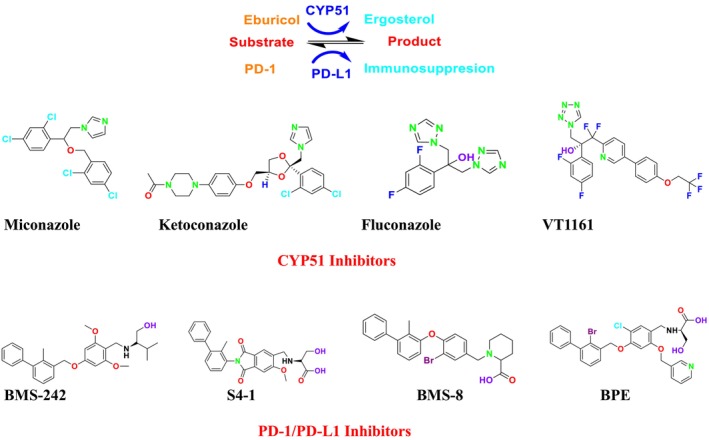
Chemical structures of CYP51 and PD‐1/PD‐L1 inhibitors (Liu, Wang, et al. 2023).

In a study conducted by Y. Liu and colleagues in 2023, PD‐L1 and CYP51 enzymes were identified as primary targets due to their key roles in fungal proliferation and immunosuppression processes. Various new bifonazole‐based compounds have been designed as PD‐L1 and CYP51 inhibitors using the FBDD method (Figure 48; Liu, Wang, et al. 2023).

**FIGURE 49:**
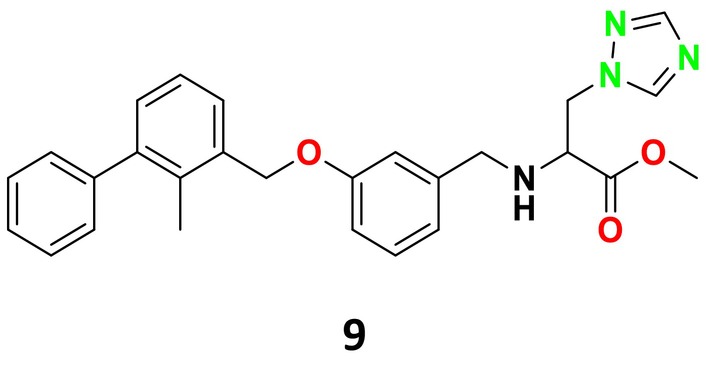
Compound 9 chemical structures.

The research findings revealed that the compounds exhibited potent antifungal activity and inhibitory properties against drug‐resistant fungal strains in vitro. In particular, compound **9** emerged as a highly effective dual‐target inhibitor, demonstrating the ability to inhibit fungal proliferation and activate the immune system (Figure 49). To enhance the bioavailability of this compound, a corresponding covalent organic framework (COF) carrier was developed. This carrier significantly accelerated the recovery process from fungal infections in vivo. Consequently, to further validate the pharmacological properties of these compounds, the relevant COF (Bt‐Bch) carrier was successfully developed and demonstrated effective targeting capabilities.

**FIGURE 50:**
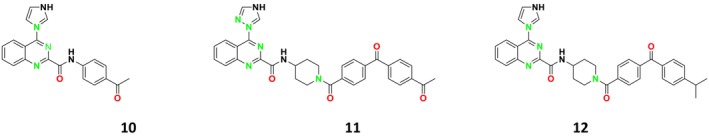
Compounds 10, 11, and 12 chemical structures.

In another study conducted by Sun and colleagues in 2023, the dual‐target inhibition of CYP51 and PD‐L1 was identified as playing a significant role in fungal proliferation and immunosuppression. In this context, new quinazoline compounds with dual‐target inhibition were developed using the scaffold growth method. The studies revealed that compounds **10, 11**, and **12** exhibited strong biological activity against various fungal strains in vitro. These compounds inhibited CYP51 activity, leading to the accumulation of reactive oxygen species and mitochondrial damage, which ultimately caused fungal cell disintegration and death (Figure 50). Additionally, by blocking the interaction between PD‐L1 and PD‐1, these compounds effectively activated the immune response, slowed down the inflammatory process, and accelerated the recovery from fungal infections.

This study demonstrated that dual‐target approaches have significant potential in antifungal drug development and may offer new strategies for treating fungal infections (Sun, Liu, et al. 2023).

### 6.5 Bromodomain‐Containing Protein 4 (BRD4)‐Histone Deacetylase (HDAC)

**FIGURE 51:**
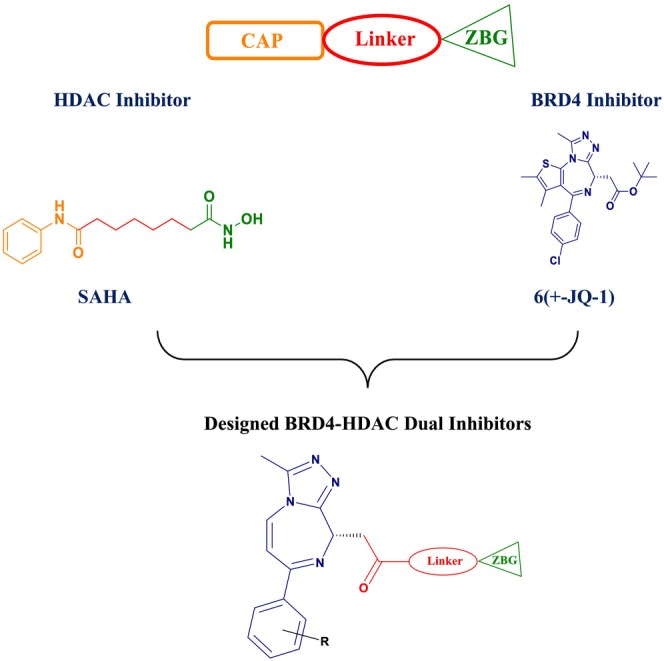
Design of BRD4–HDAC dual inhibitors (Li, Huang, et al. 2023).

**FIGURE 52:**
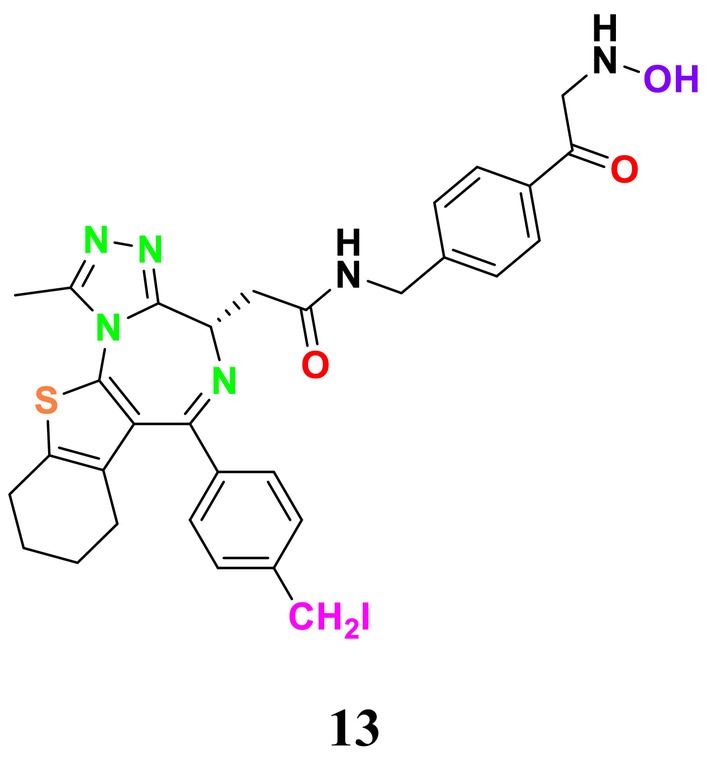
Compound 13 chemical structure.

In studies on this subject, dual inhibitors with high fungal selectivity and low toxicity to human cells have been designed by FBDD method using BRD4 and HDAC inhibitors (Figure 16). Li and colleagues have been working on developing novel BRD4‐HDAC inhibitors with the aim of restoring the sensitivity of *C. albicans* to FLC in IFIs (Figure 51). Among the developed compounds, compound 13 stood out for its high selectivity toward fungal HDACs (SI = 1653) and its excellent synergistic effect against FLC‐resistant *C. albicans* (FICI = 0.063; Figure 52). By acting in synergy with FLC, 13 inhibited biofilm formation and morphological transitions in resistant *C. albicans* , enhanced the antifungal efficacy of FLC in vivo, and significantly reduced fungal burden in the kidneys.

Mechanism studies revealed that the resistance of *C. albicans* could be reversed by the downregulation of the azole target gene CYP51, efflux pump genes, and genes associated with the extracellular matrix. This downregulation can enhance the efficacy of azoles and facilitate the entry of drugs into cells. The drug combination also reduced virulence by inhibiting changes in fungal morphology and biofilm formation. It was observed that compound 13, when used in synergy with FLC, reduced the *C. albicans* burden in a mouse model of candidiasis. These findings suggest that selective fungal HDAC inhibitors could be a promising approach for the treatment of resistant fungal infections. Structural optimization and further mechanism studies are ongoing (Li, Huang, et al. 2023).

### 6.6 *Heat Shock Protein 90* (HSP90)‐Histone Deasetilase (HDAC)

The clinical treatment of candidiasis is often hindered by increasing drug resistance and limited effectiveness. To enhance the efficacy of FLC against resistant *C. albicans* infections, the combined inhibition of HSP90 and HDAC has been proposed as a novel strategy. First‐generation HSP90/HDAC dual inhibitors, developed by Li and colleagues, have shown synergistic effects as agents for treating azole‐resistant candidiasis (Li et al. 2022).

**FIGURE 53:**
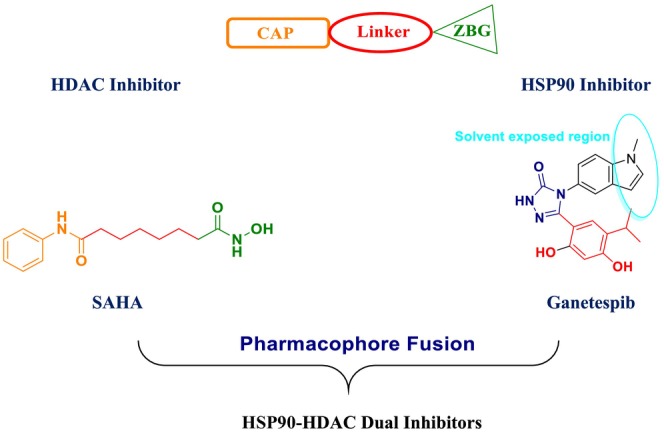
Rational design of HSP90/HDAC dual inhibitors (Li et al. 2022).

HSP90/HDAC dual inhibitors were designed by combining the pharmacophores of HSP90 and HDAC inhibitors. Ganetespib, a human HSP90 inhibitor currently in clinical trials, served as the basis for this design. While ganetespib tightly binds to the nucleotide‐binding domain of *C. albicans* HSP90, the designed dual inhibitors were integrated with diversifiable HDAC inhibitor pharmacophores. This approach lays the foundation for developing new and effective therapeutic agents for treating azole‐resistant candidiasis (Figure 53; Li et al. 2022).

**FIGURE 54:**
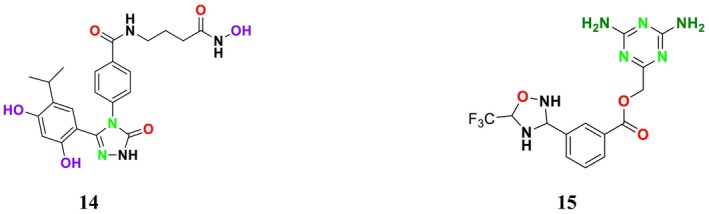
Compounds 14 and 15 chemical structures.

Notably, compound **14** (Figure 54) exhibited fungal‐selective inhibitory effects on HSP90 and HDACs, demonstrating low toxicity and excellent in vitro synergism. Additionally, in a study by Pinzi and colleagues in 2020, HDAC6 and HSP90 were extensively investigated as anticancer drug targets. They reported that compound **15** (Figure 54) selectively inhibited HDAC6 over HDAC1, increased tubulin acetylation levels in cell assays, and reduced cell proliferation (Pinzi et al. 2020).

In conclusion, HSP90/HDAC dual inhibitors present a promising strategy for developing new antifungal therapeutics to combat azole‐resistant candidiasis.

### 6.7 Januse Kinase 2 (JAK2)‐Histone Deasetilase (HDAC)

**FIGURE 55:**
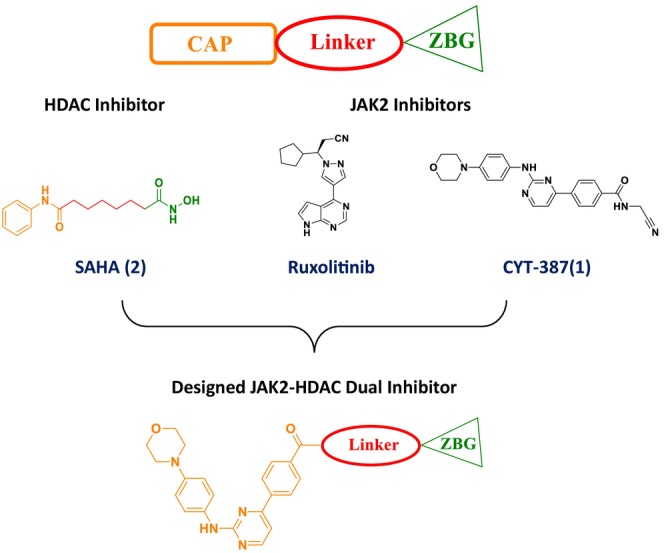
Design of JAK2‐HDAC dual inhibitors (Huang et al. 2018).

Leukemia patients often face significant challenges due to the limited efficacy of chemotherapy and the high risk of infections caused by invasive fungal pathogens. Research highlights the therapeutic potential of JAK2/HDAC dual inhibitors in treating acute myeloid leukemia and IFIs, suggesting that they could be an effective strategy in the field of multi‐target drug development. Consequently, Huang and colleagues (2018) developed a novel therapeutic strategy by designing a small molecule capable of treating both leukemia and IFIs. Their study demonstrated that newly developed JAK2 and HDAC dual inhibitors possess potent anti‐proliferative effects against hematologic cell lines and exhibit strong synergistic effects with FLC against resistant *C. albicans* infections (Figure 55; Huang et al. 2018).

**FIGURE 56:**
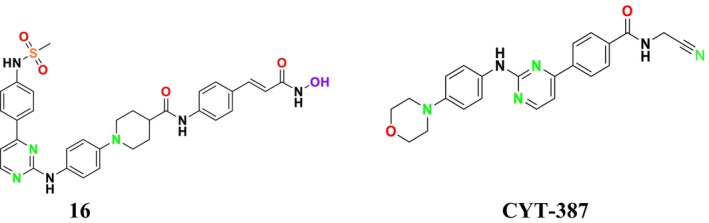
Compound 16 and CYT‐387 chemical structures.

Notably, compound 16 was identified as a highly active and selective JAK2/HDAC dual inhibitor (Figure 56). It showed significant in vivo antitumor efficacy in various AML models and demonstrated synergistic effects with FLC in treating resistant *C. albicans* infections. In the pursuit of multi‐target drug design, the structural features of typical JAK2 inhibitor pharmacophores and HDAC inhibitors were used as fundamental templates for designing dual inhibitors based on their synergistic effects.

By preserving the structural motifs of existing JAK2 inhibitors, such as CYT‐387 (Figure 56), and combining them with various structural characteristics of HDAC inhibitors, several new series of dual inhibitors were designed (Huang et al. 2018). These approaches revealed the potential of small structural modifications and substitutions to achieve effective dual inhibition against JAK2 and HDAC targets.

**FIGURE 57:**
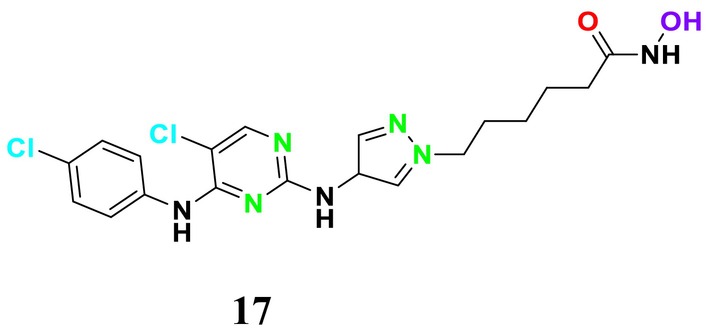
Compound 17 chemical structure.

Furthermore, a study by Liang and colleagues found that compound **17** exhibited a stronger antiproliferative effect in HEL cells carrying the JAK2 V617F mutation compared to the combination of SAHA and ruxolitinib (Figure 57; Liang et al. 2019).

The simultaneous inhibition of JAK and HDAC has been suggested to enhance the efficacy of HDAC inhibitors in treating cancers and fungal infections, addressing one of the most pressing issues today. This approach could potentially overcome resistance to HDAC inhibitors in certain tumors. Simultaneous inhibition of JAK and HDAC could enhance the efficacy of HDAC inhibitors in treating cancers and fungal infections, offering a solution to the resistance issue against HDAC inhibitors in some tumors.

### 6.8 *Bromodomain and Extra‐Terminal* (BET)‐Histone Deasetilase (HDAC)

The treatment of breast cancer alongside concurrent *C. albicans* infections presents a significant clinical challenge. In response to this, novel small molecule inhibitors targeting both BET proteins and HDACs have been designed. Among these new inhibitors, the BET family proteins have shown excellent and balanced inhibitory activity against BRD4 and HDAC1. These dual inhibitors demonstrated enhanced in vivo antitumor efficacy in MDA‐MB‐231 breast cancer xenograft models compared to either BRD4 or HDAC1 inhibitors alone. They also significantly reduced kidney fungal burden in a murine model of disseminated candidiasis when used synergistically with FLC. This indicates that BET‐HDAC dual inhibitors offer a novel therapeutic strategy for the combinational treatment of breast cancer and concurrent candidiasis.

**FIGURE 58:**
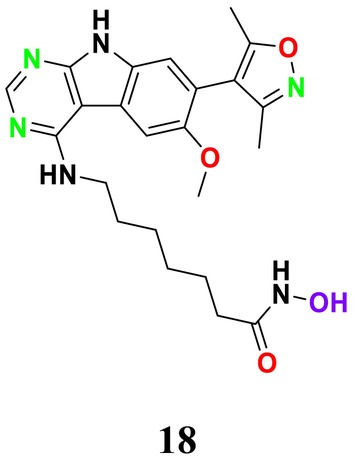
Compound 18 chemical structures.

In the design of BET‐HDAC dual inhibitors, various pan‐BET inhibitor chemotypes were explored, with the dimethylisoxazole scaffold being identified as a promising starting point due to its exceptional BET inhibitory activity and favorable pharmacokinetic profiles (Huang et al. 2023). The defined structure and excellent BRD4 inhibitory activity of compound 18 formed the basis for dual inhibitor design, and the retention of the dimethylisoxazole moiety was deemed appropriate (Figure 58).

**FIGURE 59:**
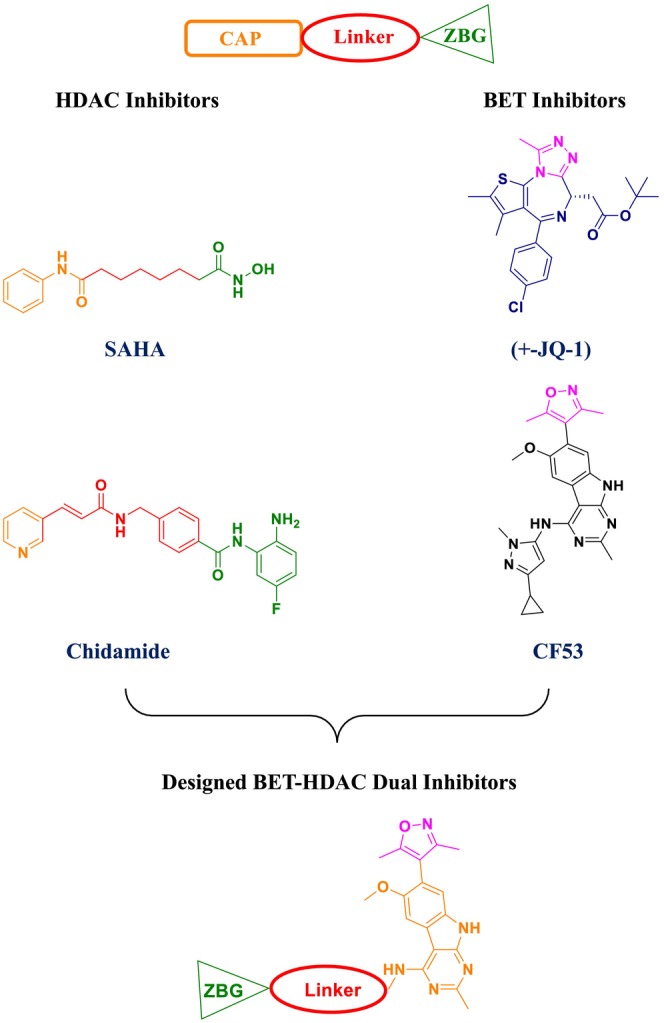
Rationale for the design of dual BET‐HDAC inhibitors (Huang et al. 2023).

The typical pharmacophore model of HDAC inhibitors was also considered in the design of these new dual inhibitors. A hydroxamic acid group and a linker were added to the scaffold of BET inhibitor 18. Additionally, strategies such as introducing a methyl group to the tricyclic scaffold and increasing the diversity of the linker were employed to enhance metabolic stability and improve the efficacy of the inhibitors (Figure 59).

## 7 Conclusion

In conclusion, the development of novel antifungal agents, both mono‐ and dual‐effective, represents a crucial advancement in the fight against IFIs. As IFIs continue to present significant challenges to global health, particularly among immunocompromised patients, the need for innovative therapeutic strategies is more critical than ever. The rapid emergence of antifungal resistance underscores the limitations of current treatment options and highlights the necessity for new antifungal drugs with distinct mechanisms of action and improved efficacy.

Antifungal agents used in the treatment of fungal infections target various components of fungal cells, including the cell membrane (azoles, morpholines, allylamines, and polyenes), the cell wall (echinocandins), or intracellular targets (antimetabolites). These drugs have been successfully utilized in antifungal therapy. However, rapid development of resistance to these agets has also been observed. Factors such as the increasing population of elderly and immunocompromised patients, the prevalence of cancer, organ transplants, immunosuppressive therapies, and surgical procedures contribute to the emergence of antifungal resistance due to prolonged drug treatments and prophylactic therapies. Mechanisms of resistance in fungi include reduced drug–target interaction (enhanced efflux), alterations in the drug's target membrane and composition, decreased oxidative damage, and the activation of alternative biosynthetic pathways for 14α‐demethylase.

Recent efforts have focused on exploring various novel targets within fungal cells, such as ERG biosynthesis pathways, cellular stress response mechanisms, and key enzymatic processes like CYP51 and HDAC. These targets offer promising avenues for the development of new antifungal agents that can overcome resistance by employing unique modes of action. Moreover, the design of dual inhibitors, which simultaneously target multiple biological pathways, provides a strategic approach to enhance antifungal activity and reduce the likelihood of resistance development.

Dual targeting with a single drug represents an innovative approach in both fungal and cancer therapy, compared to traditional drug combination strategies. The main rationale behind developing dual‐targeting agents is to address the limitations of single‐target therapies, such as incomplete efficacy and the development of drug resistance.

The incorporation of dual‐effective agents, such as CYP51‐COX and CYP51‐HDAC inhibitors, into the antifungal and anticancer therapeutic arsenal has shown potential in preclinical studies, demonstrating increased efficacy and reduced toxicity compared to single‐target agents. These innovative compounds can provide a more comprehensive attack on fungal pathogens by disrupting multiple cellular processes, thereby limiting the ability of the fungi to adapt and survive.

Overall, the continuous evolution of antifungal resistance necessitates a proactive approach to drug development, emphasizing the need for novel therapeutic agents that can effectively address the complexities of fungal infections. The ongoing research and clinical trials highlighted in this review suggest that the future of antifungal therapy lies in the strategic use of both mono‐ and dual‐effective agents, tailored to the specific needs of the patient population and the nature of the fungal pathogen. By advancing our understanding of fungal biology and leveraging cutting‐edge drug design techniques, we can hope to develop more effective treatments that improve patient outcomes and reduce the global burden of fungal diseases. As a result, dual‐targeting strategies are expected to yield more favorable outcomes in fungal and cancer treatment.

## Conflicts of Interest

The authors declare no conflicts of interest.

## Data Availability

The authors have nothing to report.

## References

[cbdd70045-bib-0001] Abbas, J. , G. P. Bodey , H. A. Hanna , et al. 2000. “Candida Krusei Fungemia.” Archives of Internal Medicine 160: 2659. 10.1001/archinte.160.17.2659. 10999981

[cbdd70045-bib-0002] Abbotsford, J. , D. A. Foley , Z. Goff , A. C. Bowen , C. C. Blyth , and D. K. Yeoh . 2021. “Clinical Experience With SUBA‐Itraconazole at a Tertiary Paediatric Hospital.” Journal of Antimicrobial Chemotherapy 76: 249–252. 10.1093/JAC/DKAA382. 32929460

[cbdd70045-bib-0003] Abe, M. , S. Nakamura , Y. Kinjo , et al. 2019. “Efficacy of T‐2307, a Novel Arylamidine, Against Ocular Complications of Disseminated Candidiasis in Mice.” Journal of Antimicrobial Chemotherapy 74: 1327–1332. 10.1093/jac/dkz020. 30753506

[cbdd70045-bib-0004] Abuhelwa, A. Y. , D. J. R. Foster , S. Mudge , D. Hayes , and R. N. Upton . 2015. “Population Pharmacokinetic Modeling of Itraconazole and Hydroxyitraconazole for Oral SUBA‐Itraconazole and Sporanox Capsule Formulations in Healthy Subjects in Fed and Fasted States.” Antimicrobial Agents and Chemotherapy 59: 5681–5696. 10.1128/AAC.00973-15. 26149987 PMC4538523

[cbdd70045-bib-0005] Adeel, A. , M. D. Qu , E. Siddiqui , S. M. Levitz , and R. T. Ellison . 2021. “Expanded Access Use of Rezafungin for Salvage Therapy of Invasive *Candida glabrata* Infection: A Case Report.” Open Forum Infectious Diseases 8: ofab431. 10.1093/ofid/ofab431. 35559122 PMC9088506

[cbdd70045-bib-0006] Adnan, A. , A. M. Borman , Z. Tóth , et al. 2023. “In Vitro Killing Activities of Anidulafungin and Micafungin With and Without Nikkomycin Z Against Four *Candida auris* Clades.” Pharmaceutics 15: 1365. 10.3390/pharmaceutics15051365. 37242607 PMC10222763

[cbdd70045-bib-0007] Ahmad, S. , and M. Asadzadeh . 2023. “Strategies to Prevent Transmission of *Candida auris* in Healthcare Settings.” Current Fungal Infection Reports 17: 36–48. 10.1007/s12281-023-00451-7. 36718372 PMC9878498

[cbdd70045-bib-0008] Aigner, M. , and C. Lass‐Flörl . 2020. “Encochleated Amphotericin B: Is the Oral Availability of Amphotericin B Finally Reached.” Journal of Fungi 6: 66. 10.3390/jof6020066. 32443486 PMC7344640

[cbdd70045-bib-0009] Alcazar‐Fuoli, L. , E. Mellado , G. Garcia‐Effron , et al. 2008. “Ergosterol Biosynthesis Pathway in *Aspergillus fumigatus* .” Steroids 73: 339–347. 10.1016/j.steroids.2007.11.005. 18191972

[cbdd70045-bib-0010] Alex, J. , K. González , T. Kindel , et al. 2020. “Caspofungin Functionalized Polymethacrylates With Antifungal Properties.” Biomacromolecules 21: 2104–2115. 10.1021/acs.biomac.0c00096. 32286800

[cbdd70045-bib-0011] Alghamdi, A. , T. Oravec , C. Nishi , et al. 2024. “Severe Hypercalcemia as a Result of Disseminated Candida Krusei Infection.” International Journal of Infectious Diseases 140: 110–112. 10.1016/j.ijid.2024.01.012. 38266977

[cbdd70045-bib-0012] Alkhazraji, S. , T. Gebremariam , A. Alqarihi , et al. 2020. “Fosmanogepix (APX001) is Effective in the Treatment of Immunocompromised Mice Infected With Invasive Pulmonary Scedosporiosis Or Disseminated Fusariosis.” https://journals.asm.org/journal/aac. 10.1128/AAC.01735-19 PMC7038288 31818813

[cbdd70045-bib-0013] Amangeldi, A. A. , B. B. Baimakhanova , L. P. Trenozhnikova , et al. 2024. “i.r. kulmagambetov, sh.m. miralimova, полиеновые антибиотики, используемые в противогрибковой терапии.” МИКРОБИОЛОГИЯ ЖӘНЕ ВИРУСОЛОГИЯ 2: 52–66. 10.53729/MV-AS.2024.02.04.

[cbdd70045-bib-0014] An, Y. , W. Liu , H. Xie , H. Fan , J. Han , and B. Sun . 2022. “Construction and Activity Evaluation of Novel Benzodioxane Derivatives as Dual‐Target Antifungal Inhibitors.” European Journal of Medicinal Chemistry 227: 113950. 10.1016/j.ejmech.2021.113950. 34731761

[cbdd70045-bib-0015] Ancuceanu, R. , M. V. Hovaneț , M. Cojocaru‐Toma , A. I. Anghel , and M. Dinu . 2022. “Potential Antifungal Targets for Aspergillus Sp. From the Calcineurin and Heat Shock Protein Pathways.” International Journal of Molecular Sciences 23: 12543. 10.3390/ijms232012543. 36293395 PMC9603945

[cbdd70045-bib-0016] Andes, D. R. , N. Safdar , J. W. Baddley , et al. 2012. “Impact of Treatment Strategy on Outcomes in Patients With Candidemia and Other Forms of Invasive Candidiasis: A Patient‐Level Quantitative Review of Randomized Trials.” Clinical Infectious Diseases 54: 1110–1122. 10.1093/cid/cis021. 22412055

[cbdd70045-bib-0017] Anthony, H. X. , and J. Muslin . 2000. “14‐3‐3 Proteins: Regulation of Subcellular Localization by Molecular Interference.” Cellular Signalling 12, no. 11‐12: 703–709. 11152955 10.1016/s0898-6568(00)00131-5

[cbdd70045-bib-0018] Apgar, J. M. , R. R. Wilkening , D. L. Parker , et al. 2021. “Ibrexafungerp: An Orally Active β‐1,3‐Glucan Synthesis Inhibitor.” Bioorganic and Medicinal Chemistry Letters 32: 127661. 10.1016/j.bmcl.2020.127661. 33160023

[cbdd70045-bib-0019] Arendrup, M. C. , E. Dzajic , R. H. Jensen , et al. 2013. “Epidemiological Changes With Potential Implication for Antifungal Prescription Recommendations for Fungaemia: Data From a Nationwide Fungaemia Surveillance Programme.” Clinical Microbiology and Infection 19: e343–e353. 10.1111/1469-0691.12212. 23607326

[cbdd70045-bib-0020] Arimoto, S. , K. Inagaki , D. Todokoro , T. Suzuki , K. Makimura , and T. Ishino . 2023. “Antifungal Efficacy of Luliconazole in an Experimental Rabbit Model of Fungal Keratitis Caused by Fusarium Solani.” Mycopathologia 188: 775–782. 10.1007/s11046-023-00783-5. 37603230

[cbdd70045-bib-0021] Aris, P. , M. Mohamadzadeh , M. Zarei , and X. Xia . 2024. “Computational Design of Novel Griseofulvin Derivatives Demonstrating Potential Antibacterial Activity: Insights From Molecular Docking and Molecular Dynamics Simulation.” International Journal of Molecular Sciences 25: 1039. 10.3390/ijms25021039. 38256112 PMC10816260

[cbdd70045-bib-0022] Aron, O. , M. Wang , L. Lin , et al. 2021. “Mogln2 Is Important for Vegetative Growth, Conidiogenesismaintenance of Cell Wall Integrity and Pathogenesis of *Magnaporthe oryzae* .” Journal of Fungi 7: 463. 10.3390/jof7060463. 34201222 PMC8229676

[cbdd70045-bib-0023] Arpitha, S. B. , B. E. Kumara Swamy , S. C. Sharma , M. R. Sanjana , and S. Varamahalakshmi . 2024. “Voltammetric Study of Dopamine at Tavaborole Modified Carbon Paste Electrode.” Sensing Technology 2: 2305873. 10.1080/28361466.2024.2305873.

[cbdd70045-bib-0024] Ashley, E. D. 2019. “Antifungal Drugs: Special Problems Treating Central Nervous System Infections.” Journal of Fungi 5: 97. 10.3390/jof5040097. 31614505 PMC6958367

[cbdd70045-bib-0025] August, B. A. , and P. B. Kale‐Pradhan . 2024. “Management of Invasive Candidiasis: A Focus on Rezafungin, Ibrexafungerp, and Fosmanogepix.” Pharmacotherapy 44: 467–479. 10.1002/phar.2926. 38721866

[cbdd70045-bib-0026] Bai, S. , M. Zhang , S. Tang , et al. 2024. “Research Progress on Benzimidazole Fungicides: A Review.” Molecules 29: 1218. 10.3390/molecules29061218. 38542855 PMC10975326

[cbdd70045-bib-0027] Baldo, A. , M. Monod , A. Mathy , et al. 2012. “Mechanisms of Skin Adherence and Invasion by Dermatophytes.” Mycoses 55: 218–223. 10.1111/j.1439-0507.2011.02081.x. 21831104

[cbdd70045-bib-0028] Banerjee, M. , I. Hatial , B. M. Keegan , and B. S. J. Blagg . 2021. “Assay Design and Development Strategies for Finding Hsp90 Inhibitors and Their Role in Human Diseases.” Pharmacology and Therapeutics 221: 107747. 10.1016/j.pharmthera.2020.107747. 33245994 PMC8744950

[cbdd70045-bib-0029] Bareich, D. C. , I. Nazi , and G. D. Wright . 2003. “Simultaneous In Vitro Assay of the First Four Enzymes in the Fungal Aspartate Pathway Identifies a New Class of Aspartate Kinase Inhibitor.” Chemistry and Biology 10: 967–973. 10.1016/j. 14583263

[cbdd70045-bib-0030] Barelle, C. J. , C. L. Priest , D. M. MacCallum , N. A. R. Gow , F. C. Odds , and A. J. P. Brown . 2006. “Niche‐Specific Regulation of Central Metabolic Pathways in a Fungal Pathogen.” Cellular Microbiology 8: 961–971. 10.1111/j.1462-5822.2005.00676.x. 16681837 PMC1472618

[cbdd70045-bib-0031] Barros, N. , R. E. Rosenblatt , M. M. Phipps , V. Fomin , and M. K. Mansour . 2023. “Invasive Fungal Infections in Liver Diseases.” Hepatology Communications 7: e0216. 10.1097/HC9.0000000000000216. 37639701 PMC10462082

[cbdd70045-bib-0032] Bassetti, M. , D. R. Giacobbe , A. Vena , and S. Esposito . 2022. “An Overview of Micafungin as a Treatment Option for Invasive Candidiasis in Pediatric Patients Younger Than 4 Months Old.” Expert Opinion on Pharmacotherapy 23: 1987–1993. 10.1080/14656566.2022.2147824. 36373395

[cbdd70045-bib-0033] Bauer, I. , and S. Graessle . 2021. “Fungal Lysine Deacetylases in Virulence, Resistance, and Production of Small Bioactive Compounds.” Genes (Basel) 12: 1470. 10.3390/genes12101470. 34680865 PMC8535771

[cbdd70045-bib-0034] Bauer, I. , M. Misslinger , Y. Shadkchan , et al. 2019. “The Lysine Deacetylase RpdA is Essential for Virulence in *Aspergillus fumigatus* .” Frontiers in Microbiology 10: 2773. 10.3389/fmicb.2019.02773. 31866965 PMC6905131

[cbdd70045-bib-0035] Berger, S. , Y. El Chazli , A. F. Babu , and A. T. Coste . 2017. “Azole Resistance in Aspergillus Fumigatus: A Consequence of Antifungal Use in Agriculture.” Frontiers in Microbiology 8: 1024. 10.3389/fmicb.2017.01024. 28638374 PMC5461301

[cbdd70045-bib-0036] Bernhardt, A. , W. Meyer , V. Rickerts , T. Aebischer , and K. Tintelnot . 2018. “Identification of 14α‐Lanosterol Demethylase (CYP51) in Scedosporium Species.” https://journals.asm.org/journal/aac. 10.1128/AAC.02599-17 PMC6105797 29891611

[cbdd70045-bib-0037] Bhattacharjee, R. , and S. Dogra . 2018. “‘End of the Road for Terbinafine’ in Dermatophytosis: Is It a Valid Conclusion?” Indian Journal of Dermatology, Venereology and Leprology 84: 706–707. 10.4103/ijdvl.IJDVL_717_18. 30289120

[cbdd70045-bib-0038] Bhattacharya, S. , B. D. Esquivel , and T. C. White . 2018. “Overexpression or Deletion of Ergosterol Biosynthesis Genes Alters Doubling Time, Response to Stress Agents, and Drug Susceptibility in *Saccharomyces cerevisiae* .” mBio 9, no. 4: e01291‐18. 10.1128/mBio.01291-18. 30042199 PMC6058291

[cbdd70045-bib-0039] Bhattacharya, S. , S. Sae‐Tia , and B. C. Fries . 2020. “Candidiasis and Mechanisms of Antifungal Resistance.” Antibiotics 9: 312. 10.3390/antibiotics9060312. 32526921 PMC7345657

[cbdd70045-bib-0040] Bibi, M. , S. Murphy , R. I. Benhamou , et al. 2021. “Combining Colistin and Fluconazole Synergistically Increases Fungal Membrane Permeability and Antifungal Cidality.” ACS Infectious Diseases 7: 377–389. 10.1021/acsinfecdis.0c00721. 33471513 PMC7887753

[cbdd70045-bib-0041] Bin Bai, Y. , M. Zhang , D. Li , Y. Zhao , L. Z. Huang , and J. M. Gao . 2023. “Synthesis and Antifungal Activity of Derivatives of the Natural Product Griseofulvin Against Phytopathogenic Fungi.” Journal of Agricultural and Food Chemistry 71: 6236–6248. 10.1021/acs.jafc.2c09037. 37061927

[cbdd70045-bib-0042] Bouz, G. , and M. Doležal . 2021. “Advances in Antifungal Drug Development: An Up‐To‐Date Mini Review.” Pharmaceuticals 14: 1312. 10.3390/ph14121312. 34959712 PMC8706862

[cbdd70045-bib-0043] Brand, S. R. , J. D. Sobel , P. Nyirjesy , M. A. Ghannoum , R. J. Schotzinger , and T. P. Degenhardt . 2021. “A Randomized Phase 2 Study of VT‐1161 for the Treatment of Acute Vulvovaginal Candidiasis.” Clinical Infectious Diseases 73: E1518–E1524. 10.1093/cid/ciaa1204. 32818963 PMC8492116

[cbdd70045-bib-0044] Brandão, F. , S. K. Esher , K. S. Ost , et al. 2018. “HDAC Genes Play Distinct and Redundant Roles in Cryptococcus Neoformans Virulence.” Scientific Reports 8: 5209. 10.1038/s41598-018-21965-y. 29581526 PMC5979944

[cbdd70045-bib-0045] Caplan, T. , E. J. Polvi , J. L. Xie , et al. 2018. “Functional Genomic Screening Reveals Core Modulators of Echinocandin Stress Responses in Candida Albicans.” Cell Reports 23: 2292–2298. 10.1016/j.celrep.2018.04.084. 29791841

[cbdd70045-bib-0046] Capodagli, G. C. , W. G. Sedhom , M. Jackson , K. A. Ahrendt , and S. D. Pegan . 2014. “A Oncompetitive Inhibitor for Mycobacterium Tuberculosis's Class Iia Fructose 1,6‐Bisphosphate Aldolase.” Biochemistry 53: 202–213. 10.1021/bi401022b. 24325645 PMC4167715

[cbdd70045-bib-0047] Carmo, A. , M. Rocha , P. Pereirinha , R. Tomé , and E. Costa . 2023. “Antifungals: From Pharmacokinetics to Clinical Practice.” Antibiotics 12: 884. 10.3390/antibiotics12050884. 37237787 PMC10215229

[cbdd70045-bib-0048] Carolus, H. , S. Pierson , K. Lagrou , and P. Van Dijck . 2020. “Amphotericin B and Other Polyenes—Discovery, Clinical Use, Mode of Action and Drug Resistance.” Journal of Fungi 6: 1–20. 10.3390/jof6040321. PMC7724567 33261213

[cbdd70045-bib-0049] Carvalho, T. 2023. “A New Class of Antibiotics Delivers Promising Trial Results Against Tuberculosis.” Nature Medicine 29: 4–5. 10.1038/d41591-022-00114-z. 36450842

[cbdd70045-bib-0050] Chandler, D. J. , A. Bonifaz , and W. W. J. van de Sande . 2023. “An Update on the Development of Novel Antifungal Agents for Eumycetoma.” Frontiers in Pharmacology 14: 1165273. 10.3389/fphar.2023.1165273. 37274106 PMC10232793

[cbdd70045-bib-0051] Chang, C. C. , and M. A. Slavin . 2017. “Albaconazole.” In Kucers' The Use of Antibiotics. London: CRC Press. https://www.taylorfrancis.com/chapters/edit/10.1201/9781498747967‐159/albaconazole‐christina‐chang‐monica‐slavin?context=ubx/.

[cbdd70045-bib-0052] Chatterjee, S. , and U. Tatu . 2017. “Heat Shock Protein 90 Localizes to the Surface and Augments Virulence Factors of Cryptococcus Neoformans.” PLoS Neglected Tropical Diseases 11: e0005836. 10.1371/journal.pntd.0005836. 28783748 PMC5559104

[cbdd70045-bib-0053] Chen, K. , L. Jennifer I , M. Munro , et al. 2024. “Ocular Candidiasis.” https://eyewiki.org/ocular_candidiasis/.

[cbdd70045-bib-0054] Chen, X. , Z. Zhang , Z. Chen , Y. Li , S. Su , and S. Sun . 2020. “Potential Antifungal Targets Based on Glucose Metabolism Pathways of Candida Albicans.” Frontiers in Microbiology 11: 296. 10.3389/fmicb.2020.00296. 32256459 PMC7093590

[cbdd70045-bib-0055] Chua, N. K. , H. W. Coates , and A. J. Brown . 2020. “Squalene Monooxygenase: A Journey to the Heart of Cholesterol Synthesis.” Progress in Lipid Research 79: 101033. 10.1016/j.plipres.2020.101033. 32360125

[cbdd70045-bib-0056] Cleveland Clinic . 2021. “Antifungal Resistance.” https://my.clevelandclinic.org/health/articles/21557‐antifungal‐resistance/.

[cbdd70045-bib-0057] Connolly, L. 2023. “New Treatment for Invasive Fungal Infection Candidiasis Approved by FDA.” https://health.ucdavis.edu/news/headlines/new‐treatment‐for‐invasive‐fungal‐infection‐candidiasis‐approved‐by‐fda/.

[cbdd70045-bib-0058] Costantino, L. , S. Ferrari , M. Santucci , et al. 2022. “Destabilizers of the Thymidylate Synthase Homodimer Accelerate Its Proteasomal Degradation and Inhibit Cancer Growth.” eLife 11: e73862. 10.7554/ELIFE.73862. 36475542 PMC9831607

[cbdd70045-bib-0059] Cowen, L. E. , D. Sanglard , S. J. Howard , P. D. Rogers , and D. S. Perlin . 2015. “Mechanisms of Antifungal Drug Resistance.” Cold Spring Harbor Perspectives in Medicine 5: a019752. 10.1101/cshperspect.a019752. PMC4484955 25384768

[cbdd70045-bib-0060] Cowen, L. E. , and W. J. Steinbach . 2008. “Stress, Drugs, and Evolution: The Role of Cellular Signaling in Fungal Drug Resistance.” Eukaryotic Cell 7: 747–764. 10.1128/EC.00041-08. 18375617 PMC2394973

[cbdd70045-bib-0061] Crasto, A. 2014. “Fosravuconazole in Phase 1 for the Treatment of Fungal Infections.” https://newdrugapprovals.org/2014/09/08/fosravuconazole‐in‐phase‐1‐for‐the‐treatment‐of‐fungal‐infections/.

[cbdd70045-bib-0062] Crasto, M. 2024. “Ravuconazole.” https://newdrugapprovals.org/2014/09/07/ravuconazole/.

[cbdd70045-bib-0063] Dahal, G. P. , and R. E. Viola . 2018. “A Fragment Library Screening Approach to Identify Selective Inhibitors Against an Essential Fungal Enzyme.” SLAS Discovery 23: 520–531. 10.1177/2472555218767844. 29608391

[cbdd70045-bib-0064] Davis, M. R. , M. A. Donnelley , and G. R. Thompson . 2020. “Ibrexafungerp: A Novel Oral Glucan Synthase Inhibitor.” Medical Mycology 58: 579–592. 10.1093/mmy/myz083. 31342066

[cbdd70045-bib-0065] Davood, A. , Y. EbrahimiNassimi , S. Sardari , and Y. F. Farahani . 2023. “N‐Unsubstituted Imidazoles: Design, Synthesis, and Antimicrobial Evaluation.” Current Pharmaceutical Design 29: 1875–1881. 10.2174/1381612829666230807120704. 37550905

[cbdd70045-bib-0066] De Francesco, M. A. 2023. “Drug‐Resistant Aspergillus Spp.: A Literature Review of Its Resistance Mechanisms and Its Prevalence in Europe.” Pathogens 12: 1305. 10.3390/pathogens12111305. 38003770 PMC10674884

[cbdd70045-bib-0067] De Pascale, G. , E. J. Griffiths , T. Shakya , I. Nazi , and G. D. Wright . 2011. “Identification and Characterization of New Inhibitors of Fungal Homoserine Kinase.” Chembiochem 12: 1179–1182. 10.1002/cbic.201100121. 21538764

[cbdd70045-bib-0068] Denning, D. W. 2003. “Echinocandin Antifungal Drugs.” Lancet 362: 1142–1151. 10.1016/S0140-6736(03)14472-8. 14550704

[cbdd70045-bib-0069] Denning, D. W. 2024. “Global Incidence and Mortality of Severe Fungal Disease.” Lancet Infectious Diseases 24, no. 7: e428–e438. 10.1016/S1473-3099(23)00692-8. 38224705

[cbdd70045-bib-0070] Derkacz, D. , P. Bernat , and A. Krasowska . 2022. “K143R Amino Acid Substitution in 14α‐Demethylase (ERG11p) Changes Plasma Membrane and Cell Wall Structure of *Candida albicans* .” International Journal of Molecular Sciences 23: 1631. 10.3390/ijms23031631. 35163552 PMC8836035

[cbdd70045-bib-0071] Dick, J. D. , W. G. Merz , and A. R. Saral . 1980. “Incidence of polyene‐resistant yeasts recovered from clinical specimens.” Antimicrobial Agents and Chemotherapy 18, no. 1: 158–163. https://journals.asm.org/journal/aac. 7416742 10.1128/aac.18.1.158 PMC283956

[cbdd70045-bib-0072] Dietl, A.‐M. , M. Misslinger , M. M. Aguiar , et al. 2019. “The Siderophore Transporter Sit1 Determines Susceptibility to the Antifungal VL‐2397.” Antimicrobial Agents and Chemotherapy 63: e00807‐19. 10.1128/AAC.00807-19. 31405865 PMC6761561

[cbdd70045-bib-0073] Dladla, M. , M. Gyzenhout , G. Marias , and S. Ghosh . 2024. “Azole Resistance in *Aspergillus fumigatus* – Comprehensive Review.” Archives of Microbiology 206: 305. 10.1007/s00203-024-04026-z. 38878211

[cbdd70045-bib-0074] Dong, Y. , M. Liu , J. Wang , Z. Ding , and B. Sun . 2019. “Construction of Antifungal Dual‐Target (SE, CYP51) Pharmacophore Models and the Discovery of Novel Antifungal Inhibitors.” RSC Advances 9: 26302–26314. 10.1039/c9ra03713f. 35531010 PMC9070380

[cbdd70045-bib-0075] Doorley, L. A. 2023. “Investigation of Clinically Relevant Fluconazole Resistance Mechanisms in the Fungal Pathogen *Candida parapsilosis*.” University of Tennessee Health Science Center. 10.21007/etd.cghs.2023.0620.

[cbdd70045-bib-0076] Drugs.com . 2024. “Luliconazole Prescribing Information.” https://www.drugs.com/pro/luliconazole.html#_ref/.

[cbdd70045-bib-0077] Du, J. , Y. Dong , H. Zhao , et al. 2023. “Transcriptional Regulation of Autophagy, Cell Wall Stress Response and Pathogenicity by Pho23 in *C. albicans* .” FEBS Journal 290: 855–871. 10.1111/febs.16636. 36152022

[cbdd70045-bib-0078] Dufourc, E. J. 2008. “Sterols and Membrane Dynamics.” Journal of Chemical Biology 1: 63–77. 10.1007/s12154-008-0010-6. 19568799 PMC2698314

[cbdd70045-bib-0079] Efimova, S. S. , L. V. Schagina , and O. S. Ostroumova . 2014. “Investigation of Channel‐Forming Activity of Polyene Macrolide Antibiotics in Planar Lipid Bilayers in the Presence of Dipole Modifiers.” Acta Naturae 6: 67–79. 25558397 PMC4273094

[cbdd70045-bib-0080] Elghblawi, E. 2017. “Tinea Capitis in Children and Trichoscopic Criteria.” International Journal of Trichology 9: 47–49. 28839385 10.4103/ijt.ijt_54_16 PMC5551304

[cbdd70045-bib-0081] Elias, R. , P. Basu , and M. Fridman . 2022. “Fluconazole‐COX Inhibitor Hybrids: A Dual‐Acting Class of Antifungal Azoles.” Journal of Medicinal Chemistry 65: 2361–2373. 10.1021/acs.jmedchem.1c01807. 35084852 PMC8842223

[cbdd70045-bib-0082] Ellsworth, M. , and L. Ostrosky‐Zeichner . 2020. “Isavuconazole: Mechanism of Action, Clinical Efficacy, and Resistance.” Journal of Fungi 6: 1–10. 10.3390/jof6040324. PMC7712939 33260353

[cbdd70045-bib-0083] Enoch, D. A. , H. Yang , S. H. Aliyu , and C. Micallef . 2017. “The Changing epidemiology of Invasive fungal Infections.” Methods in Molecular Biology 1508: 17–65. 10.1007/978-1-4939-6515-1_2. 27837497

[cbdd70045-bib-0084] Ergün, M. , A. M. E. Jansen , L. B. Hilbrands , et al. 2024. “Isavuconazole as Prophylaxis and Therapy for Invasive Fungal Diseases: A Real‐Life Observational Study.” Journal of Antimicrobial Chemotherapy 79: 1801–1810. 10.1093/jac/dkae139. 38935893 PMC11290874

[cbdd70045-bib-0085] Fang, W. , J. Wu , M. Cheng , et al. 2023. “Diagnosis of Invasive Fungal Infections: Challenges and Recent Developments.” Journal of Biomedical Science 30: 42. 10.1186/s12929-023-00926-2. 37337179 PMC10278348

[cbdd70045-bib-0086] Fenton, A. , and G. K. John . 2024. “Candida Auris Resistance Mechanisms to Amphotericin b Alternative Treatments Development.” Current Clinical Microbiology Reports 11: 166–176. 10.1007/s40588-024-00233-w.

[cbdd70045-bib-0087] Fisher, M. C. , A. Alastruey‐Izquierdo , J. Berman , et al. 2022. “Tackling the Emerging Threat of Antifungal Resistance to Human Health.” Nature Reviews. Microbiology 20: 557–571. 10.1038/s41579-022-00720-1. 35352028 PMC8962932

[cbdd70045-bib-0088] Flowers, S. A. , B. Colón , S. G. Whaley , M. A. Schuler , and P. David Rogers . 2015. “Contribution of Clinically Derived Mutations in ERG11 to Azole Resistance in *Candida albicans* .” Antimicrobial Agents and Chemotherapy 59: 450–460. 10.1128/AAC.03470-14. 25385095 PMC4291385

[cbdd70045-bib-0089] Flyway Pharmacy . 2024. “Griseofulvin: Mechanism of Action and Uses.” https://www.flywaypharmacy.com/2024/02/12/griseofulvin‐mechanism‐of‐action‐and‐uses/.

[cbdd70045-bib-0090] Fry, S. C. 2017. “Cells.” In Encyclopedia of Applied Plant Sciences, 174–184. Amsterdam: Elsevier. 10.1016/B978-0-12-394807-6.00119-2.

[cbdd70045-bib-0091] Gangneux, J. P. , O. Lortholary , O. A. Cornely , and L. Pagano . 2019. “9th Trends in Medical Mycology Held on 11–14 October 2019, Nice, France, Organized Under the Auspices of EORTC‐IDG and ECMM.” Journal of Fungi 5: 95. 10.3390/jof5040095. 31597285 PMC7807382

[cbdd70045-bib-0092] García‐García, I. , and A. M. Borobia . 2021. “Current Approaches and Future Strategies for the Implementation of Pharmacogenomics in the Clinical Use of Azole Antifungal Drugs.” Expert Opinion on Drug Metabolism and Toxicology 17: 509–514. 10.1080/17425255.2021.1890715. 33622115

[cbdd70045-bib-0093] Gaziano, R. , E. Campione , F. Iacovelli , et al. 2018. “Antifungal Activity of *Cardiospermum halicacabum* L. (Sapindaceae) Against *Trichophyton rubrum* Occurs Through Molecular Interaction With Fungal Hsp90.” Drug Design, Development and Therapy 12: 2185–2193. 10.2147/DDDT.S155610. 30034223 PMC6047602

[cbdd70045-bib-0094] Gazzinelli, B. P. , C. M. Brêtas , and I. C. César . 2022. “Development of a Stability‐Indicating Assay Method by HPLC‐DAD and MS Characterization of Forced Degradation Products of Ravuconazole.” Journal of Chromatographic Science 60: 157–163. 10.1093/chromsci/bmab064. 34075394

[cbdd70045-bib-0095] Ghannoum, M. , M. C. Arendrup , V. P. Chaturvedi , et al. 2020. “Ibrexafungerp: A Novel Oral Triterpenoid Antifungal in Development for the Treatment of Candida Auris Infections.” Antibiotics 9: 539. 10.3390/antibiotics9090539. 32854252 PMC7559578

[cbdd70045-bib-0096] Ghannoum, M. A. , and L. B. Rice . 1999. “Antifungal Agents: Mode of Action, Mechanisms of Resistance, and Correlation of These Mechanisms With Bacterial Resistance.” Clinical Microbiology Reviews 12, no. 4: 501–517. https://journals.asm.org/journal/cmr. 10515900 10.1128/cmr.12.4.501 PMC88922

[cbdd70045-bib-0097] Gintjee, T. J. , M. A. Donnelley , and G. R. Thompson . 2020. “Aspiring Antifungals: Review of Current Antifungal Pipeline Developments.” Journal of Fungi 6: 28. 10.3390/jof6010028. 32106450 PMC7151215

[cbdd70045-bib-0098] Girstmair, H. , F. Tippel , A. Lopez , et al. 2019. “The Hsp90 Isoforms From *S. cerevisiae* Differ in Structure, Function and Client Range.” Nature Communications 10: 3626. 10.1038/s41467-019-11518-w. PMC6689086 31399574

[cbdd70045-bib-0099] GlobalData . 2024. “Likelihood of Approval and Phase Transition Success Rate Model—Fosravuconazole in Tinea Pedis (Athlete Foot).” https://www.globaldata.com/store/report/fosravuconazole‐in‐tinea‐pedis‐athlete‐foot‐loa‐innovation‐and‐trend‐analysis/?utm_source=lgp5‐loa&utm_medium=24‐231800&utm_campaign=thematic‐report‐hyperlink/.

[cbdd70045-bib-0100] Gogineni, V. , and M. T. Hamann . 1862. “Marine Natural Product Peptides With Therapeutic Potential: Chemistry, Biosynthesis, and Pharmacology.” Biochimica et Biophysica Acta ‐ General Subjects 2018: 81–196. 10.1016/j.bbagen.2017.08.014. PMC5918664 28844981

[cbdd70045-bib-0101] Groll, A. H. , B. J. A. Rijnders , T. J. Walsh , J. Adler‐Moore , R. E. Lewis , and R. J. M. Brüggemann . 2019. “Clinical Pharmacokinetics, Pharmacodynamics, Safety and Efficacy of Liposomal Amphotericin B.” Clinical Infectious Diseases 68: S260–S274. 10.1093/cid/ciz076. 31222253 PMC6495018

[cbdd70045-bib-0102] Gupta, A. K. , and D. Daigle . 2016. “A Critical Appraisal of Once‐Daily Topical Luliconazole for the Treatment of Superficial Fungal Infections.” Infection and Drug Resistance 9: 1–6. 10.2147/IDR.S61998. 26848272 PMC4723097

[cbdd70045-bib-0103] Gupta, A. K. , K. A. Foley , and S. G. Versteeg . 2017. “New Antifungal Agents and New Formulations Against Dermatophytes.” Mycopathologia 182: 127–141. 10.1007/s11046-016-0045-0. 27502503

[cbdd70045-bib-0104] Gupta, A. K. , R. R. Mays , S. G. Versteeg , et al. 2018. “Tinea Capitis in Children: A Systematic Review of Management.” Journal of the European Academy of Dermatology and Venereology 32: 2264–2274. 10.1111/jdv.15088. 29797669

[cbdd70045-bib-0105] Haas, H. 2012. “Iron‐ a Key Nexus in the Virulence of Aspergillus Fumigatus.” Frontiers in Microbiology 3: 28. 10.3389/fmicb.2012.00028. 22347220 PMC3272694

[cbdd70045-bib-0106] Haas, H. 2014. “Fungal Siderophore Metabolism With a Focus on Aspergillus Fumigatus.” Natural Product Reports 31: 1266–1276. 10.1039/C4NP00071D. 25140791 PMC4162504

[cbdd70045-bib-0107] Hamill, R. J. 2013. “Amphotericin B Formulations: A Comparative Review of Efficacy and Toxicity.” Drugs 73: 919–934. 10.1007/s40265-013-0069-4. 23729001

[cbdd70045-bib-0108] Hammoudi Halat, D. , S. Younes , N. Mourad , and M. Rahal . 2022. “Allylamines, Benzylamines, and Fungal Cell Permeability: A Review of Mechanistic Effects and Usefulness Against Fungal Pathogens.” Membranes (Basel) 12: 1171. 10.3390/membranes12121171. 36557078 PMC9781035

[cbdd70045-bib-0109] Han, G. , N. Liu , C. Li , J. Tu , Z. Li , and C. Sheng . 2020. “Discovery of Novel Fungal Lanosterol 14α‐Demethylase (CYP51)/Histone Deacetylase Dual Inhibitors to Treat Azole‐Resistant Candidiasis.” Journal of Medicinal Chemistry 63: 5341–5359. 10.1021/acs.jmedchem.0c00102. 32347094

[cbdd70045-bib-0110] Han, X. , X. Zhu , Z. Hong , et al. 2017. “Structure‐Based Rational Design of Novel Inhibitors Against Fructose‐1,6‐Bisphosphate Aldolase From *Candida albicans* .” Journal of Chemical Information and Modeling 57: 1426–1438. 10.1021/acs.jcim.6b00763. 28475320

[cbdd70045-bib-0111] Hanaoka, K. , K. Nishikawa , A. Ikeda , et al. 2023. “Membrane Contact Sites Regulate Vacuolar Fission via Sphingolipid Metabolism.” Elife 12: RP89938. 10.7554/eLife.89938. PMC10972560 38536872

[cbdd70045-bib-0112] Happacher, I. , M. Aguiar , M. Alilou , et al. 2023. “The Siderophore Ferricrocin Mediates Iron Acquisition in Aspergillus Fumigatus.” Microbiology Spectrum 11: e0049623. 10.1128/spectrum.00496-23. 37199664 PMC10269809

[cbdd70045-bib-0113] Hargrove, T. Y. , E. P. Garvey , W. J. Hoekstra , et al. 2017. “Crystal Structure of the New Investigational Drug Candidate VT‐1598 in Complex With Aspergillus Fumigatus Sterol 14α‐Demethylase Provides Insights Into Its Broad‐Spectrum Antifungal Activity.” Antimicrobial Agents and Chemotherapy 61: e00570‐17. 10.1128/AAC.00570-17. 28461309 PMC5487673

[cbdd70045-bib-0114] Hee Lee, S. , M. El‐Agamy Farh , J. Lee , et al. 2021. “A Histone Deacetylase, Magnaporthe Oryzae Rpd3, Regulates Reproduction and Pathogenic Development in the Rice Blast Fungus.” http://hme.riceblast.snu.ac.kr. 10.1128/mBio.02600-21 PMC8593672 34781734

[cbdd70045-bib-0115] Hoekstra, W. J. , E. P. Garvey , W. R. Moore , S. W. Rafferty , C. M. Yates , and R. J. Schotzinger . 2014. “Design and Optimization of Highly‐Selective Fungal CYP51 Inhibitors.” Bioorganic and Medicinal Chemistry Letters 24: 3455–3458. 10.1016/j.bmcl.2014.05.068. 24948565

[cbdd70045-bib-0116] Hoenigl, M. , R. Sprute , M. Egger , et al. 2021. “The Antifungal Pipeline: Fosmanogepix, Ibrexafungerp, Olorofim, Opelconazole, and Rezafungin.” Drugs 81: 1703–1729. 10.1007/s40265-021-01611-0. 34626339 PMC8501344

[cbdd70045-bib-0117] Hon, K. L. E. , V. P. Chan , A. K. Leung , K. K. Y. Leung , and W. F. Hui . 2024. “Invasive Fungal Infections in Critically Ill Children: Epidemiology, Risk Factors and Antifungal Drugs.” Drugs in Context 13: 2023‐9‐2. 10.7573/dic.2023-9-2. PMC11195526 38915918

[cbdd70045-bib-0118] Hosing, C. , Z. Braunstein , E. McLaughlin , et al. 2023. “Post‐Allograft Romidepsin Maintenance Mitigates Relapse Risk and Stimulates the Graft‐Versus‐Malignancy Effect Through Enhanced NK‐Cell Cytotoxicity in Patients With t‐Cell Malignancies: Final Results of a Phase I/II Trial.” Blood 142: 184. 10.1182/blood-2023-190213.

[cbdd70045-bib-0119] Houšť, J. , J. Spížek , and V. Havlíček . 2020. “Antifungal drugs.” Metabolites 10: 106. 10.3390/metabo10030106. 32178468 PMC7143493

[cbdd70045-bib-0120] Howard, K. C. , E. K. Dennis , D. S. Watt , and S. Garneau‐Tsodikova . 2020. “A Comprehensive Overview of the Medicinal Chemistry of Antifungal Drugs: Perspectives and Promise.” Chemical Society Reviews 49: 2426–2480. 10.1039/C9CS00556K. 32140691

[cbdd70045-bib-0121] Howard, S. J. , and M. C. Arendrup . 2011. “Acquired Antifungal Drug Resistance in Aspergillus Fumigatus: Epidemiology and Detection.” Medical Mycology 49: S90–S95. 10.3109/13693786.2010.508469. 20795765

[cbdd70045-bib-0122] Hsiang, T. , and D. L. Baillie . 2005. “Comparison of the Yeast Proteome to Other Fungal Genomes to Find Core Fungal Genes.” Journal of Molecular Evolution 60: 475–483. 10.1007/s00239-004-0218-1. 15883882

[cbdd70045-bib-0123] Hsiung, E. , A. Celebioglu , M. E. Kilic , E. Durgun , and T. Uyar . 2023. “Fast‐Disintegrating Nanofibrous Web of Pullulan/Griseofulvin‐Cyclodextrin Inclusion Complexes.” Molecular Pharmaceutics 20: 2624–2633. 10.1021/acs.molpharmaceut.3c00074. 37014780

[cbdd70045-bib-0124] Hu, Z. , B. He , L. Ma , Y. Sun , Y. Niu , and B. Zeng . 2017. “Recent Advances in Ergosterol Biosynthesis and Regulation Mechanisms in Saccharomyces Cerevisiae.” Indian Journal of Microbiology 57: 270–277. 10.1007/s12088-017-0657-1. 28904410 PMC5574775

[cbdd70045-bib-0125] Huang, D. S. , E. V. Leblanc , T. Shekhar‐Guturja , et al. 2020. “Design and Synthesis of Fungal‐Selective Resorcylate Aminopyrazole Hsp90 Inhibitors.” Journal of Medicinal Chemistry 63: 2139–2180. 10.1021/acs.jmedchem.9b00826. 31513387 PMC7069776

[cbdd70045-bib-0126] Huang, Y. , G. Dong , H. Li , N. Liu , W. Zhang , and C. Sheng . 2018. “Discovery of Janus Kinase 2 (JAK2) and Histone Deacetylase (HDAC) Dual Inhibitors as a Novel Strategy for the Combinational Treatment of Leukemia and Invasive Fungal Infections.” Journal of Medicinal Chemistry 61: 6056–6074. 10.1021/acs.jmedchem.8b00393. 29940115

[cbdd70045-bib-0127] Huang, Y. , N. Liu , Z. Pan , Z. Li , and C. Sheng . 2023. “BET‐HDAC Dual Inhibitors for Combinational Treatment of Breast Cancer and Concurrent Candidiasis.” Journal of Medicinal Chemistry 66: 1239–1253. 10.1021/acs.jmedchem.2c01191. 36622852

[cbdd70045-bib-0128] Hüttel, W. 2021. “Echinocandins: Structural Diversity, Biosynthesis, and Development of Antimycotics.” Applied Microbiology and Biotechnology 105, no. 1: 55–66. 10.1007/s00253-020-11022-y/. 33270153 PMC7778625

[cbdd70045-bib-0129] Hwang, G. J. , J. Roh , S. Son , et al. 2023. “Induction of Fungal Secondary Metabolites by Co‐Culture With Actinomycete Producing HDAC Inhibitor Trichostatins.” Journal of Microbiology and Biotechnology 33: 1437–1447. 10.4014/jmb.2301.01017. 37670557 PMC10699267

[cbdd70045-bib-0130] Ishii, M. , T. Yamada , and S. Ohata . 2024. “An Efficient Gene Targeting System Using Δku80 and Functional Analysis of Cyp51A in Trichophyton Rubrum.” AMB Express 14, no. 1: 96. 39215862 10.1186/s13568-024-01755-8 PMC11365917

[cbdd70045-bib-0131] Jenner, E. 2020. “iKIX1 Inhibits the Interaction of the CgGal11A KIX Domain and the CgPdr1 Activation Domain.” https://www.immune‐system‐research.com/2020/11/10/ikix1‐inhibits‐the‐interaction‐of‐the‐cggal11a‐kix‐domain‐and‐the‐cgpdr1‐activation‐domain/.

[cbdd70045-bib-0132] Jiang, K. , P. Luo , X. Wang , and L. Lu . 2024. “Insight Into Advances for the Biosynthetic Progress of Fermented Echinocandins of Antifungals.” Microbial Biotechnology 17: e14359. 10.1111/1751-7915.14359. 37885073 PMC10832530

[cbdd70045-bib-0133] Johnston, E. J. , T. Moses , and S. J. Rosser . 2020. “The Wide‐Ranging Phenotypes of Ergosterol Biosynthesis Mutants, and Implications for Microbial Cell Factories.” Yeast 37: 27–44. 10.1002/yea.3452. 31800968

[cbdd70045-bib-0134] Johnston, P. B. , A. F. Cashen , P. G. Nikolinakos , et al. 2021. “Belinostat in Combination With Standard Cyclophosphamide, Doxorubicin, Vincristine and Prednisone as First‐Line Treatment for Patients With Newly Diagnosed Peripheral T‐Cell Lymphoma.” Experimental Hematology and Oncology 10: 15. 10.1186/s40164-021-00203-8. 33602316 PMC7893947

[cbdd70045-bib-0135] Jordá, T. , and S. Puig . 2020. “Regulation of Ergosterol Biosynthesis in *Saccharomyces cerevisiae* .” Genes 11: 795. 10.3390/genes11070795. 32679672 PMC7397035

[cbdd70045-bib-0136] Joseph‐Horne, T. , and D. W. Hollomon . 2006. “Molecular Mechanisms of Azole Resistance in Fungi.” FEMS Microbiology Letters 149: 141–149. 10.1111/j.1574-6968.1997.tb10321.x. 9141655

[cbdd70045-bib-0137] Joshua, I. M. , and T. Höfken . 2017. “From Lipid Homeostasis to Differentiation: Old and New Functions of the Zinc Cluster Proteins Ecm22, Upc2, Sut1 and Sut2.” International Journal of Molecular Sciences 18: 772. 10.3390/ijms18040772. 28379181 PMC5412356

[cbdd70045-bib-0138] Ju, Z. , Z. Li , M. Li , S. Xu , K. Kaliaperumal , and F.‐E. Chen . 2023. “A Chemo‐Enzymatic Approach for Preparing Efinaconazole With High Optical Yield.” Journal of Organic Chemistry 88: 14803–14808. 10.1021/acs.joc.3c01641. 37792295

[cbdd70045-bib-0139] Kaluzhskiy, L. , E. Yablokov , O. Gnedenko , et al. 2024. “The Effect of Membrane Composition on the Interaction Between Human CYP51 and Its Flavonoid Inhibitor‐ Luteolin 7,3′‐Disulfate.” Biochimica et Biophysica Acta ‐ Biomembranes 1866: 184286. 10.1016/j.bbamem.2024.184286. 38272204

[cbdd70045-bib-0140] Kanafani, Z. A. , and J. R. Perfect . 2008. “Resistance to Antifungal Agents: Mechanisms and Clinical Impact.” Clinical Infectious Diseases 46: 120–128. 10.1086/524071. 18171227

[cbdd70045-bib-0141] Kapoor, M. , M. Moloney , Q. A. Soltow , C. M. Pillar , and K. J. Shaw . 2020. “Evaluation of Resistance Development to the GWT1 Inhibitor Manogepix (APX001A) in Candida Species.” Antimicrobial Agents and Chemotherapy 64: e01387‐19. 10.1128/AAC.01387-19. PMC7187586 31611349

[cbdd70045-bib-0142] Kelly, S. L. , D. C. Lamb , D. E. Kelly , et al. 1997. “Resistance to Fluconazole and Cross‐Resistance to Amphotericin B in *Candida albicans* From AIDS Patients Caused by Defective Sterol Δ5,6‐Desaturation.” FEBS Letters 400: 80–82. 10.1016/S0014-5793(96)01360-9. 9000517

[cbdd70045-bib-0143] Khan, J. , A. rani , M. Aslam , R. S. Maharia , G. Pandey , and B. Nand . 2024. “Exploring Triazole‐Based Drugs: Synthesis, Application, FDA Approvals, and Clinical Trial Updates–A Comprehensive Review.” Tetrahedron 162: 134122. 10.1016/j.tet.2024.134122.

[cbdd70045-bib-0144] Khurana, A. , A. Masih , A. Chowdhary , et al. 2018. “Correlation of in vitro susceptibility based on mics and squalene epoxidase mutations with clinical response to terbinafine in patients with Tinea corporis/cruris.” Antimicrobial Agents and Chemotherapy 62: e01038‐18. 10.1128/AAC.01038-18. 30275090 PMC6256768

[cbdd70045-bib-0145] Kodedová, M. , and H. Sychrová . 2015. “Changes in the Sterol Composition of the Plasma Membrane Affect Membrane Potential, Salt Tolerance and the Activity of Multidrug Resistance Pumps in *Saccharomyces cerevisiae* .” PLoS One 10: e0139306. 10.1371/journal.pone.0139306. 26418026 PMC4587746

[cbdd70045-bib-0146] Kovanda, L. L. , S. M. Sullivan , L. R. Smith , A. V. Desai , P. L. Bonate , and W. W. Hope . 2019. “Population Pharmacokinetic Modeling of VL‐2397, a Novel Systemic Antifungal Agent: Analysis of a Single‐ and Multiple‐Ascending‐Dose Study in Healthy Subjects.” Antimicrobial Agents and Chemotherapy 63: e00163‐19. 10.1128/AAC.00163-19. 30988142 PMC6535549

[cbdd70045-bib-0147] Kreijkamp‐Kaspers, S. , K. Hawke , L. Guo , et al. 2017. “Oral Antifungal Medication for Toenail Onychomycosis.” Cochrane Database of Systematic Reviews 2017: CD010031. 10.1002/14651858.CD010031.pub2. PMC6483327 28707751

[cbdd70045-bib-0148] Kriegl, L. , M. Egger , J. Boyer , M. Hoenigl , and R. Krause . 2024. “New Treatment Options for Critically Important WHO Fungal Priority Pathogens.” Clinical Microbiology and Infection. 10.1016/j.cmi.2024.03.006. 38461942

[cbdd70045-bib-0149] Kristanc, L. , B. Božič , Š. Z. Jokhadar , M. S. Dolenc , and G. Gomišček . 2019. “The Pore‐Forming Action of Polyenes: From Model Membranes to Living Organisms.” Biochimica et Biophysica Acta ‐ Biomembranes 1861: 418–430. 10.1016/j.bbamem.2018.11.006. 30458121

[cbdd70045-bib-0150] Kröber, A. , S. Etzrodt , M. Bach , et al. 2017. “The Transcriptional Regulators SteA and StuA Contribute to Keratin Degradation and Sexual Reproduction of the Dermatophyte Arthroderma Benhamiae.” Current Genetics 63: 103–116. 10.1007/s00294-016-0608-0. 27170358

[cbdd70045-bib-0151] Kuhnert, E. , Y. Li , N. Lan , et al. 2018. “Enfumafungin synthase represents a novel lineage of fungal triterpene cyclases.” Environmental Microbiology 20, no. 9: 3325–3342. 30051576 10.1111/1462-2920.14333 PMC6237087

[cbdd70045-bib-0152] Kumar, R. , R. Rajkumar , V. Diwakar , N. Khan , G. K. Meghwanshi , and P. Garg . 2024. “Structural–Functional Analysis of Drug Target Aspartate Semialdehyde Dehydrogenase.” Drug Discovery Today 29: 103908. 10.1016/j.drudis.2024.103908. 38301800

[cbdd70045-bib-0153] Kuplińska, A. , and K. Rząd . 2021. “Molecular Targets for Antifungals in Amino Acid and Protein Biosynthetic Pathways.” Amino Acids 53: 961–991. 10.1007/s00726-021-03007-6. 34081205 PMC8241756

[cbdd70045-bib-0154] Lafayette, S. L. , C. Collins , A. K. Zaas , et al. 2010. “PKC Signaling Regulates Drug Resistance of the Fungal Pathogen *Candida albicans* via Circuitry Comprised of mkc1, Calcineurin, and hsp90.” PLoS Pathogens 6: 79–80. 10.1371/journal.ppat.1001069. PMC2928802 20865172

[cbdd70045-bib-0155] Leber, R. , R. Zenz , K. Schröttner , S. Fuchsbichler , B. Pühringer , and F. Turnowsky . 2001. “A Novel Sequence Element Is Involved in the Transcriptional Regulation of Expression of the ERG1 (Squalene Epoxidase) Gene in *Saccharomyces cerevisiae* .” European Journal of Biochemistry 268: 914–924. 10.1046/j.1432-1327.2001.01940.x. 11179957

[cbdd70045-bib-0156] Léchenne, B. , U. Reichard , C. Zaugg , et al. 2007. “Sulphite Efflux Pumps in *Aspergillus fumigatus* and Dermatophytes.” Microbiology 153: 905–913. 10.1099/mic.0.2006/003335-0. 17322211

[cbdd70045-bib-0157] Lee, H. , and D. G. Lee . 2018. “Novel Approaches for Efficient Antifungal Drug Action.” Journal of Microbiology and Biotechnology 28: 1771–1781. 10.4014/jmb.1807.07002. 30178649

[cbdd70045-bib-0158] Lee, Y. , N. Robbins , and L. E. Cowen . 2023. “Molecular Mechanisms Governing Antifungal Drug Resistance.” Npj Antimicrobials and Resistance 1: 5. 10.1038/s44259-023-00007-2. 38686214 PMC11057204

[cbdd70045-bib-0159] Lepak, A. J. , M. Zhao , and D. R. Andesa . 2018. “Pharmacodynamic Evaluation of Rezafungin (CD101) Against Candida Auris in the Neutropenic Mouse Invasive Candidiasis Model.” Antimicrobial Agents and Chemotherapy 62: e01572‐18. 10.1128/AAC.01572-18. 30181375 PMC6201117

[cbdd70045-bib-0160] Lepesheva, G. I. , L. Friggeri , and M. R. Waterman . 2018. “CYP51 as Drug Targets for Fungi and Protozoan Parasites: Past, Present and Future.” Parasitology 145: 1820–1836. 10.1017/S0031182018000562. 29642960 PMC6185833

[cbdd70045-bib-0161] Li, C. , J. Tu , G. Han , N. Liu , and C. Sheng . 2022. “Heat Shock Protein 90 (Hsp90) /Histone Deacetylase (HDAC) Dual Inhibitors for the Treatment of Azoles‐Resistant Candida Albicans.” European Journal of Medicinal Chemistry 227: 113961. 10.1016/j.ejmech.2021.113961. 34742014

[cbdd70045-bib-0162] Li, G. , L. Chen , H. Bai , L. Zhang , J. Wang , and W. Li . 2024. “Depletion of Squalene Epoxidase in Synergy With Glutathione Peroxidase 4 Inhibitor RSL3 Overcomes Oxidative Stress Resistance in Lung Squamous Cell Carcinoma.” Precision Clinical Medicine 7: pbae011. 10.1093/pcmedi/pbae011. 38779359 PMC11109822

[cbdd70045-bib-0163] Li, H. , D. Xu , X. Tan , et al. 2023. “The Role of Trehalose Biosynthesis on Mycolate Composition and L‐Glutamate Production in *Corynebacterium glutamicum* .” Microbiological Research 267: 127260. 10.1016/j.micres.2022.127260. 36463830

[cbdd70045-bib-0164] Li, P. , J. Ge , and H. Li . 2020. “Lysine Acetyltransferases and Lysine Deacetylases as Targets for Cardiovascular Disease.” Nature Reviews. Cardiology 17: 96–115. 10.1038/s41569-019-0235-9. 31350538

[cbdd70045-bib-0165] Li, Y. , Y. Zhao , H. Peng , et al. 2021. “Histone Deacetylase Inhibitor Trichostatin a Reduces Endothelial Cell Proliferation by Suppressing STAT5A‐Related Gene Transcription.” Frontiers in Oncology 11: 746266. 10.3389/fonc.2021.746266. 34650929 PMC8506210

[cbdd70045-bib-0166] Li, Z. , Y. Huang , J. Tu , et al. 2023. “Discovery of BRD4‐HDAC Dual Inhibitors With Improved Fungal Selectivity and Potent Synergistic Antifungal Activity Against Fluconazole‐Resistant *Candida albicans* .” Journal of Medicinal Chemistry 66: 5950–5964. 10.1021/acs.jmedchem.3c00165. 37037787

[cbdd70045-bib-0167] Liang, X. , J. Zang , X. Li , et al. 2019. “Discovery of Novel Janus Kinase (JAK) and Histone Deacetylase (HDAC) Dual Inhibitors for the Treatment of Hematological Malignancies.” Journal of Medicinal Chemistry 62: 3898–3923. 10.1021/acs.jmedchem.8b01597. 30901208

[cbdd70045-bib-0168] Lin, E. S. , and C. Y. Huang . 2024. “Binding Pattern and Structural Interactome of the Anticancer Drug 5‐Fluorouracil: A Critical Review.” International Journal of Molecular Sciences 25: 3404. 10.3390/ijms25063404. 38542377 PMC10970046

[cbdd70045-bib-0169] Lindsay, J. , S. Mudge , and G. R. Thompson . 2018. “Effects of Food and Omeprazole on a Novel Formulation of Super Bioavailability Itraconazole in Healthy Subjects.” Antimicrobial Agents and Chemotherapy 62: e01723‐18. 10.1128/AAC.01723-18. PMC6256753 30297369

[cbdd70045-bib-0170] Liu, J. , K. A. Vanderwyk , M. A. Donnelley , and G. R. Thompson III . 2024. “SUBA‐Itraconazole in the Treatment of Systemic Fungal Infections.” Future Microbiology 19: 1171–1175. 10.1080/17460913.2024.2362128. 39011995 PMC11529195

[cbdd70045-bib-0171] Liu, J.‐F. , J.‐J. Xia , K.‐L. Nie , F. Wang , and L. Deng . 2019. “Outline of the Biosynthesis and Regulation of Ergosterol in Yeast.” World Journal of Microbiology and Biotechnology 35: 98. 10.1007/s11274-019-2673-2. 31222401

[cbdd70045-bib-0172] Liu, M. , K. Zhang , Q. Li , et al. 2023. “Recent Advances on Small‐Molecule Bromodomain‐Containing Histone Acetyltransferase Inhibitors.” Journal of Medicinal Chemistry 66: 1678–1699. 10.1021/acs.jmedchem.2c01638. 36695774

[cbdd70045-bib-0173] Liu, W. , Y. Liu , H. Fan , et al. 2022. “Design, Synthesis, and Biological Evaluation of Dual‐Target COX‐2/CYP51 Inhibitors for the Treatment of Fungal Infectious Diseases.” Journal of Medicinal Chemistry 65: 12219–12239. 10.1021/acs.jmedchem.2c00878. 36074863

[cbdd70045-bib-0174] Liu, W. , L. Yuan , and S. Wang . 2020. “Recent Progress in the Discovery of Antifungal Agents Targeting the Cell Wall.” Journal of Medicinal Chemistry 63: 12429–12459. 10.1021/acs.jmedchem.0c00748. 32692166

[cbdd70045-bib-0175] Liu, Y. , Q. Wang , S. Yu , M. Liu , J. Han , and B. Sun . 2023. “Construction and Evaluation of Novel Dual‐Function Antifungal Inhibitors and Covalent Organic Framework Carriers Based on the Infection Microenvironment.” Journal of Medicinal Chemistry 66: 13838–13857. 10.1021/acs.jmedchem.3c01372. 37752076

[cbdd70045-bib-0176] Logviniuk, D. , Q. Z. Jaber , R. Dobrovetsky , et al. 2022. “Benzylic Dehydroxylation of Echinocandin Antifungal Drugs Restores Efficacy Against Resistance Conferred by Mutated Glucan Synthase.” Journal of the American Chemical Society 144: 5965–5975. 10.1021/jacs.2c00269. 35347986 PMC8991007

[cbdd70045-bib-0177] Lokeswari, R. , S. Pal , and D. Naveen . 2024. “Antifungal Resistance in Animals: A Brief Note.” Vet Farm Frontier e‐Magazine 1: 9–12.

[cbdd70045-bib-0178] Lu, H. , T. Hong , Y. Jiang , M. Whiteway , and S. Zhang . 2023. “Candidiasis: From Cutaneous to Systemic, New Perspectives of Potential Targets and Therapeutic Strategies.” Advanced Drug Delivery Reviews 199: 114960. 10.1016/j.addr.2023.114960. 37307922

[cbdd70045-bib-0179] Lv, Q. Z. , L. Yan , and Y. Y. Jiang . 2016. “The Synthesis, Regulation, and Functions of Sterols in *Candida albicans*: Well‐Known but Still Lots to Learn.” Virulence 7: 649–659. 10.1080/21505594.2016.1188236. 27221657 PMC4991322

[cbdd70045-bib-0180] Lyu, X. , C. Zhao , Z. M. Yan , and H. Hua . 2016. “Efficacy of Nystatin for the Treatment of Oral Candidiasis: A Systematic Review and Meta‐Analysis.” Drug Design, Development and Therapy 10: 1161–1171. 10.2147/DDDT.S100795. 27042008 PMC4801147

[cbdd70045-bib-0181] Mabiala‐Bassiloua, C. G. , G. Arthus‐Cartier , V. Hannaert , H. Thérisod , J. Sygusch , and M. Thérisod . 2011. “Mannitol Bis‐Phosphate Based Inhibitors of Fructose 1,6‐Bisphosphate Aldolases.” ACS Medicinal Chemistry Letters 2: 804–808. 10.1021/ml200129s. 24900268 PMC4018070

[cbdd70045-bib-0182] Maione, A. , A. La Pietra , A. Siciliano , et al. 2022. “The Arylamidine T‐2307 as a Novel Treatment for the Prevention and Eradication of Candida Tropicalis Biofilms.” International Journal of Molecular Sciences 23: 16042. 10.3390/ijms232416042. 36555687 PMC9786618

[cbdd70045-bib-0183] Matthews, R. C. , G. Rigg , S. Hodgetts , et al. 2003. “Preclinical Assessment of the Efficacy of Mycograb, a Human Recombinant Antibody Against Fungal HSP90.” Antimicrobial Agents and Chemotherapy 47: 2208–2216. 10.1128/AAC.47.7.2208-2216.2003. 12821470 PMC161838

[cbdd70045-bib-0184] McCarthy, M. W. , D. P. Kontoyiannis , O. A. Cornely , J. R. Perfect , and T. J. Walsh . 2017. “Novel Agents and Drug Targets to Meet the Challenges of Resistant Fungi.” Journal of Infectious Diseases 216: S474–S483. 10.1093/infdis/jix130. 28911042

[cbdd70045-bib-0185] McCoy, M. 2022. “The FDA Approves New Antifungal.” Chemical and Engineering News 100: 19.

[cbdd70045-bib-0186] McLellan, C. A. , L. Whitesell , O. D. King , A. K. Lancaster , R. Mazitschek , and S. Lindquist . 2012. “Inhibiting GPI Anchor Biosynthesis in Fungi Stresses the Endoplasmic Reticulum and Enhances Immunogenicity.” ACS Chemical Biology 7: 1520–1528. 10.1021/cb300235m. 22724584

[cbdd70045-bib-0187] Mehravar, S. , G. S. Leite , M. Pimentel , and A. Rezaie . 2024. “Antifungal Effects of Echinocandins Diminish When Exposed to Intestinal Lumen Contents: A Finding With Potentially Significant Clinical Implications.” Frontiers in Pharmacology 15: 1376656. 10.3389/fphar.2024.1376656. 38601473 PMC11004442

[cbdd70045-bib-0188] Mehta, D. , V. Saini , and A. Bajaj . 2023. “Recent Developments in Membrane Targeting Antifungal Agents to Mitigate Antifungal Resistance.” RSC Medicinal Chemistry 14: 1603–1628. 10.1039/d3md00151b. 37731690 PMC10507810

[cbdd70045-bib-0189] Mersinli, C. 2020. “Antifungal Ilaçlar.” https://www.konsultasyon.net/antifungal‐ilaclar/.

[cbdd70045-bib-0190] Mesa‐Arango, A. C. , L. Scorzoni , and O. Zaragoza . 2012. “It Only Takes One to Do Many Jobs: Amphotericin B as Antifungal and Immunomodulatory Drug.” Frontiers in Microbiology 3: 286. 10.3389/fmicb.2012.00286. 23024638 PMC3441194

[cbdd70045-bib-0191] Mesquida, A. , J. Díaz‐García , C. Sánchez‐Carrillo , P. Muñoz , P. Escribano , and J. Guinea . 2022. “In Vitro Activity of Ibrexafungerp Against Candida Species Isolated From Blood Cultures, Determination of Wild‐Type Populations Using the EUCAST Method.” Clinical Microbiology and Infection 28: 140.e1–140.e4. 10.1016/j.cmi.2021.09.030. 34619396

[cbdd70045-bib-0192] MethylGene Inc . 2013. “Methylgene Reports Results of Phase 2 Trial of MGCD290.” https://www.biospace.com/methylgene‐reports‐results‐of‐phase‐2‐trial‐of‐mgcd290/.

[cbdd70045-bib-0193] Misas, E. , E. Seagle , E. N. Jenkins , et al. 2024. “Genomic Description of Acquired Fluconazole‐ and Echinocandin‐Resistance in Patients With Serial Candida Glabrata Isolates.” Journal of Clinical Microbiology 62: e0114023. 10.1128/jcm.01140-23. 38265207 PMC10865870

[cbdd70045-bib-0194] Miyazaki, M. , T. Horii , K. Hata , et al. 2011. “In Vitro Activity of E1210, a Novel Antifungal, Against Clinically important Yeasts and Molds.” Antimicrobial Agents and Chemotherapy 55: 4652–4658. 10.1128/AAC.00291-11. 21825291 PMC3186989

[cbdd70045-bib-0195] Mohan, M. , and A. Rudroju . 2024. “Optimization and Characterization of Hyper Cross‐Linked Cyclodextrins for Improved Efinaconazole Delivery: A Comprehensive Study.” Journal of Applied Pharmaceutical Science 14, no. 8: 216–229. 10.7324/JAPS.2024.180236.

[cbdd70045-bib-0196] Mood, A. D. , I. D. U. A. Premachandra , S. Hiew , et al. 2017. “Potent Antifungal Synergy of Phthalazinone and isoquinolones With Azoles Against *Candida albicans* .” ACS Medicinal Chemistry Letters 8: 168–173. 10.1021/acsmedchemlett.6b00355. 28197306 PMC5304299

[cbdd70045-bib-0197] Mudenda, S. 2024. “Global Burden of Fungal Infections and Antifungal Resistance From 1961 to 2024: Findings and Future implications.” Pharmacology and Pharmacy 15: 81–112. 10.4236/pp.2024.154007.

[cbdd70045-bib-0198] Muhammad Ismail, F. , I. Ahmad , and E. Javed . 2021. “Metadata Analysis of the Squalene Epoxidase Gene in Dermatophytes.” International Journal of Endorsing Health Science Research 9: 129–142. 10.29052/ijehsr.v9.i1.2021.129-142.

[cbdd70045-bib-0329] Mukhopadhyay, R. 2012. “Vincent Allfrey's work on histone acetylation.” Journal of Biological Chemistry 287: 2270–2271.

[cbdd70045-bib-0199] Murakami, Y. , U. Siripanyapinyo , Y. Hong , et al. 2003. “PIG‐W Is Critical for Inositol Acylation but Not for Flipping of Glycosylphosphatidylinositol‐Anchor.” Molecular Biology of the Cell 14: 4285–4295. 10.1091/mbc.E03-03-0193. 14517336 PMC207019

[cbdd70045-bib-0200] Nakamura, I. , K. Ohsumi , S. Takeda , et al. 2019. “ASp2397 Is a Novel Natural Compound That Exhibits Rapid and Potent Fungicidal Activity Against Aspergillus Species Through a Specific Transporter.” Antimicrobial Agents and Chemotherapy 63: e02689‐18. 10.1128/AAC.02689-18. 31405853 PMC6761492

[cbdd70045-bib-0201] Nakamura, I. , S. Yoshimura , T. Masaki , et al. 2017. “ASP2397: A Novel Antifungal Agent Produced by Acremonium Persicinum MF‐347833.” Journal of Antibiotics 70: 45–51. 10.1038/ja.2016.107. 27599768

[cbdd70045-bib-0202] Neoh, C. F. , W. Jeong , D. C. Kong , and M. A. Slavin . 2023. “The Antifungal Pipeline for Invasive Fungal Diseases: What Does the Future Hold?” Expert Review of Anti‐Infective Therapy 21: 577–594. 10.1080/14787210.2023.2203383. 37057677

[cbdd70045-bib-0203] Nett, J. E. , and D. R. Andes . 2016. “Antifungal Agents.” Infectious Disease Clinics of North America 30: 51–83. 10.1016/j.idc.2015.10.012. 26739608

[cbdd70045-bib-0204] Ni, T. , Y. Hao , Z. Ding , et al. 2024. “Discovery of a Novel Potent Tetrazole Antifungal Candidate With High Selectivity and Broad Spectrum.” Journal of Medicinal Chemistry 67: 6238–6252. 10.1021/acs.jmedchem.3c02188. 38598688

[cbdd70045-bib-0205] Nield, B. , S. R. Larsen , and S. J. Van Hal . 2019. “Clinical Experience With New Formulation SUBA‐Itraconazole for Prophylaxis in Patients Undergoing Stem Cell Transplantation or Treatment for Haematological Malignancies.” Journal of Antimicrobial Chemotherapy 74: 3049–3055. 10.1093/jac/dkz303. 31360992

[cbdd70045-bib-0206] Nishimoto, A. T. , N. P. Wiederhold , S. A. Flowers , et al. 2019. “In Vitro Activities of the Novel Investigational Tetrazoles VT‐1161 and VT‐1598 Compared To The Triazole Antifungals Against Azole‐Resistant Strains and Clinical Isolates of *Candida albicans* .” Antimicrobial Agents and Chemotherapy 10: e00341‐19. 10.1128/AAC.00341-19 PMC6535515 30910896

[cbdd70045-bib-0207] Nunnally, N. S. , K. A. Etienne , D. Angulo , S. R. Lockhart , and E. L. Berkow . 2019. “In Vitro Activity of Ibrexafungerp, a Novel Glucan Synthase Inhibitor against *Candida glabrata* Isolates with FKS Mutations.” Antimicrobial Agents and Chemotherapy 63, no. 11: e01692‐19. 10.1128/AAC.01692-19. PMC6811432 31481447

[cbdd70045-bib-0208] Nyirjesy, P. , J. R. Schwebke , D. A. Angulo , I. A. Harriott , N. E. Azie , and J. D. Sobel . 2022. “Phase 2 Randomized Study of Oral Ibrexafungerp Versus Fluconazole in Vulvovaginal Candidiasis.” Clinical Infectious Diseases 74: 2129–2135. 10.1093/cid/ciab841. 34555149 PMC9258939

[cbdd70045-bib-0209] Odds, F. C. , A. J. P. Brown , and N. A. R. Gow . 2003. “Antifungal Agents: Mechanisms of Action.” Trends in Microbiology 11: 272–279. 10.1016/S0966-842X(03)00117-3. 12823944

[cbdd70045-bib-0210] Odiba, A. S. , O. A. Durojaye , I. M. Ezeonu , A. C. Mgbeahuruike , and B. C. Nwanguma . 2022. “A New Variant of Mutational and Polymorphic Signatures in the ERG11 Gene of Fluconazole‐Resistant Candida Albicans.” Infection and Drug Resistance 15: 3111–3133. 10.2147/IDR.S360973. 35747333 PMC9213107

[cbdd70045-bib-0211] Oliver, J. D. , G. E. M. Sibley , N. Beckmann , et al. 2016. “F901318 Represents a Novel Class of Antifungal Drug That Inhibits Dihydroorotate Dehydrogenase.” Proceedings of the National Academy of Sciences of the United States of America 113: 12809–12814. 10.1073/pnas.1608304113. 27791100 PMC5111691

[cbdd70045-bib-0212] O'Meara, T. R. , N. Robbins , and L. E. Cowen . 2017. “The Hsp90 Chaperone Network Modulates Candida Virulence Traits.” Trends in Microbiology 25: 809–819. 10.1016/j.tim.2017.05.003. 28549824 PMC5610082

[cbdd70045-bib-0213] Osset‐Trénor, P. , A. Pascual‐Ahuir , and M. Proft . 2023. “Fungal Drug Response and Antimicrobial Resistance.” Journal of Fungi 9: 565. 10.3390/jof9050565. 37233275 PMC10219139

[cbdd70045-bib-0214] Ou, X. , S. Li , Y. Chen , H. Rong , A. Li , and M. Lu . 2022. “Polymorphism in Griseofulvin: New Story Between an Old Drug and Polyethylene Glycol.” Crystal Growth and Design 22: 3778–3785. 10.1021/acs.cgd.2c00156.

[cbdd70045-bib-0215] Overgaauw, A. J. C. , D. C. De Leeuw , S. P. Stoof , K. Van Dijk , J. C. J. Bot , and E. J. Hendriks . 2020. “Case Report: Candida Krusei Spondylitis in an Immunocompromised Patient.” BMC Infectious Diseases 20: 739. 10.1186/s12879-020-05451-3. 33032533 PMC7542866

[cbdd70045-bib-0216] Owens, J. 2003. “References and Links A Helping Hand.” www.nature.com/reviews/drugdisc.

[cbdd70045-bib-0217] Padmavathi, A. R. , G. K. K. Reddy , P. S. Murthy , and Y. V. Nancharaiah . 2024. “New Arsenals for Old Armour: Biogenic Nanoparticles in the Battle Against Drug‐Resistant Candida Albicans.” Microbial Pathogenesis 194: 106800. 10.1016/j.micpath.2024.106800. 39025380

[cbdd70045-bib-0218] Pappas, P. G. , C. A. Kauffman , D. R. Andes , et al. 2015. “Clinical Practice Guideline for the Management of Candidiasis: 2016 Update by the Infectious Diseases Society of America.” Clinical Infectious Diseases 62: e1–e50. 10.1093/cid/civ933. 26679628 PMC4725385

[cbdd70045-bib-0219] Park, S. Y. , and J. S. Kim . 2020. “A Short Guide to Histone Deacetylases Including Recent Progress on Class II Enzymes.” Experimental and Molecular Medicine 52: 204–212. 10.1038/s12276-020-0382-4. 32071378 PMC7062823

[cbdd70045-bib-0220] Pascon, R. C. , T. M. Ganous , J. M. Kingsbury , G. M. Cox , and J. H. McCusker . 2004. “Cryptococcus Neoformans Methionine Synthase: Expression Analysis and Requirement for Virulence.” Microbiology 150: 3013–3023. 10.1099/mic.0.27235-0. 15347759

[cbdd70045-bib-0221] Pérez‐Cantero, A. , L. López‐Fernández , J. Guarro , and J. Capilla . 2020. “Azole Resistance Mechanisms in Aspergillus: Update and Recent Advances.” International Journal of Antimicrobial Agents 55: 105807. 10.1016/j.ijantimicag.2019.09.011. 31542320

[cbdd70045-bib-0222] Perfect, J. R. 2017. “The Antifungal Pipeline: A Reality Check.” Nature Reviews. Drug Discovery 16: 603–616. 10.1038/nrd.2017.46. 28496146 PMC5760994

[cbdd70045-bib-0223] Perlin, D. S. 2015. “Mechanisms of Echinocandin Antifungal Drug Resistance.” Annals of the New York Academy of Sciences 1354: 1–11. 10.1111/nyas.12831. 26190298 PMC4626328

[cbdd70045-bib-0224] Pfaller, M. A. , S. A. Messer , N. Georgopapadakou , L. A. Martell , J. M. Besterman , and D. J. Diekema . 2009. “Activity of MGCD290, a Hos2 Histone Deacetylase Inhibitor, in Combination With Azole Antifungals Against Opportunistic Fungal Pathogens.” Journal of Clinical Microbiology 47: 3797–3804. 10.1128/JCM.00618-09. 19794038 PMC2786684

[cbdd70045-bib-0225] Pfaller, M. A. , P. R. Rhomberg , S. A. Messer , and M. Castanheira . 2015. “In Vitro Activity of a Hos2 Deacetylase Inhibitor, MGCD290, in Combination With Echinocandins Against Echinocandin‐Resistant Candida Species.” Diagnostic Microbiology and Infectious Disease 81: 259–263. 10.1016/j.diagmicrobio.2014.11.008. 25600842

[cbdd70045-bib-0226] Pfizer . 2018. “An Evaluation of the Safety and Pharmacokinetics of Tavaborole Topical Solution for the Treatment of Fungal Disease of the Toenail in Children and Adolescents.” https://Clinicaltrials.Gov/Study/NCT03405818?Cond=tavaborole&term=antifungals&rank=3/.

[cbdd70045-bib-0227] Phillips, N. A. , M. Rocktashel , and L. Merjanian . 2023. “Ibrexafungerp for the Treatment of Vulvovaginal Candidiasis: Design, Development and Place in Therapy.” Drug Design, Development and Therapy 17: 363–367. 10.2147/DDDT.S339349. 36785761 PMC9921437

[cbdd70045-bib-0228] Pintye, A. , R. Bacsó , and G. M. Kovács . 2024. “Trans‐Kingdom Fungal Pathogens Infecting Both Plants and Humans, and the Problem of Azole Fungicide Resistance.” Frontiers in Microbiology 15: 1354757. 10.3389/fmicb.2024.1354757. 38410389 PMC10896089

[cbdd70045-bib-0229] Pinzi, L. , R. Benedetti , L. Altucci , and G. Rastelli . 2020. “Design of Dual Inhibitors of Histone Deacetylase 6 and Heat Shock Protein 90.” ACS Omega 5: 11473–11480. 10.1021/acsomega.0c00559. 32478236 PMC7254527

[cbdd70045-bib-0230] Poester, V. R. , L. S. Munhoz , D. A. Stevens , et al. 2023. “Nikkomycin Z for the Treatment of Experimental Sporotrichosis Caused by Sporothrix Brasiliensis.” Mycoses 66: 898–905. 10.1111/myc.13629. 37434420

[cbdd70045-bib-0231] Posch, W. , M. Blatzer , D. Wilflingseder , and C. Lass‐Flörl . 2018. “Aspergillus Terreus: Novel Lessons Learned on Amphotericin B Resistance.” Medical Mycology 56: S73–S82. 10.1093/mmy/myx119. 29538736

[cbdd70045-bib-0232] Prajapati, S. K. , A. Jain , and M. Bajpai . 2024. “Development and Validation of the RP‐HPLC Method for Quantification of Tavaborole.” Analytical Methods 16: 5280–5287. 10.1039/d4ay00943f. 39016030

[cbdd70045-bib-0233] Prayag, P. S. , S. A. Patwardhan , R. S. Joshi , S. Dhupad , T. Rane , and A. P. Prayag . 2024. “Comparative Efficacies of the Three Echinocandins for *Candida auris* Candidemia: Real World Evidence From a Tertiary Centre in India.” Medical Mycology 62: myae065. 10.1093/mmy/myae065. 38918058 PMC11250272

[cbdd70045-bib-0234] Pristov, K. E. , and M. A. Ghannoum . 2019. “Resistance of Candida to Azoles and Echinocandins Worldwide.” Clinical Microbiology and Infection 25: 792–798. 10.1016/j.cmi.2019.03.028. 30965100

[cbdd70045-bib-0235] Pulmocide Ltd . 2021. “Pulmocide's Lead Drug Candidate Opelconazole (PC945) Granted Orphan Drug, Fast Track and Qualified Infectious Disease Product Designations by US FDA.” Https://www.globenewswire.com/news‐release/2021/09/15/2297356/0/en/pulmocide‐s‐lead‐drug‐candidate‐opelconazole‐pc945‐granted‐orphan‐drug‐fast‐track‐and‐qualified‐infectious‐disease‐product‐designations‐by‐US‐FDA.Html/.

[cbdd70045-bib-0236] Pulmocide Ltd . 2023. “Pulmocide Announces New Clinical Data Confirming Low Potential for Drug–Drug Interactions With Inhaled Opelconazole.” https://pulmocide.com/press‐release/pulmocide‐announces‐new‐clinical‐data‐confirming‐low‐potential‐for‐drug‐drug‐interactions‐with‐inhaled‐opelconazole/.

[cbdd70045-bib-0237] Rabaan, A. A. , T. Sulaiman , S. H. Al‐Ahmed , et al. 2023. “Potential Strategies to Control the Risk of Antifungal Resistance in Humans: A Comprehensive Review.” Antibiotics 12: 608. 10.3390/antibiotics12030608. 36978475 PMC10045400

[cbdd70045-bib-0238] Ramana, K. V. , S. Kandi , V. Bharatkumar P , et al. 2013. “Invasive Fungal Infections: A Comprehensive Review.” American Journal of Infectious Diseases and Microbiology 1: 64–69. 10.12691/ajidm-1-4-2.

[cbdd70045-bib-0239] Rani, M. , K. Parekh , T. Mehta , and A. Omri . 2024. “Formulation Development and Characterization of Luliconazole Loaded−Mesoporous Silica Nanoparticles (MCM − 48) as Topical Hydrogel for the Treatment of Cutaneous Candidiasis.” Journal of Drug Delivery Science and Technology 91: 105250. 10.1016/j.jddst.2023.105250.

[cbdd70045-bib-0240] Rauseo, A. M. , A. Coler‐Reilly , L. Larson , and A. Spec . 2020. “Hope on the Horizon: Novel Fungal Treatments in Development.” Open Forum Infectious Diseases 7: ofaa016. 10.1093/ofid/ofaa016. 32099843 PMC7031074

[cbdd70045-bib-0241] Rivero‐Menendez, O. , M. Cuenca‐Estrella , and A. Alastruey‐Izquierdo . 2019. “In Vitro Activity of Olorofim (F901318) Against Clinical Isolates of Cryptic Species of Aspergillus by EUCAST and CLSI Methodologies.” Journal of Antimicrobial Chemotherapy 74: 1586–1590. 10.1093/jac/dkz078. 30891600

[cbdd70045-bib-0242] Robbins, N. , T. Caplan , and L. E. Cowen . 2017. “Molecular Evolution of Antifungal Drug Resistance.” Annual Review of Microbiology 71: 753–775. 10.1146/annurev-micro-030117. 28886681

[cbdd70045-bib-0243] Robbins, N. , G. D. Wright , and L. E. Cowen . 2016. “Antifungal Drugs: The Current Armamentarium and Development of New Agents.” Microbiology Spectrum 4, no. 5: 10–1128. 10.1128/microbiolspec.funk-0002-2016. 27763259

[cbdd70045-bib-0244] Rodrigues, M. L. 2018. “The Multifunctional Fungal Ergosterol.” MBio 9: e01755‐18. 10.1128/mBio.01755-18. PMC6143734 30228244

[cbdd70045-bib-0245] Roemer, T. , and D. J. Krysan . 2014. “Antifungal Drug Development: Challenges, Unmet Clinical Needs, and New Approaches.” Cold Spring Harbor Perspectives in Medicine 4: a019703. 10.1101/cshperspect.a019703. 24789878 PMC3996373

[cbdd70045-bib-0246] Rudramurthy, S. M. , S. A. Shankarnarayan , S. Dogra , et al. 2018. “Mutation in the Squalene Epoxidase Gene of Trichophyton Interdigitale and Trichophyton Rubrum Associated With Allylamine Resistance.” https://journals.asm.org/journal/aac. 10.1128/AAC.02522-17 PMC5923174 29530857

[cbdd70045-bib-0247] Şahiner, F. , and S. N. Altintaş . 2021. “Antifungal Agents in the Treatment of Candidosis and Susceptibility Tests.” Journal of Molecular Virology and Immunology 2: 56–66. 10.46683/jmvi.2021.32.

[cbdd70045-bib-0248] Sandison, T. , V. Ong , J. Lee , and D. Thye . 2017. “Safety and Pharmacokinetics of CD101 IV, a Novel Echinocandin, in Healthy Adults.” Antimicrobial Agents and Chemotherapy 61: e01627‐16. 10.1128/AAC.01627-16. 27919901 PMC5278714

[cbdd70045-bib-0249] Sanglard, D. , A. Coste , and S. Ferrari . 2009. “Antifungal Drug Resistance Mechanisms in Fungal Pathogens From the Perspective of Transcriptional Gene Regulation.” FEMS Yeast Research 9: 1029–1050. 10.1111/j.1567-1364.2009.00578.x. 19799636

[cbdd70045-bib-0250] Sanglard, D. , F. Ischer , T. Parkinson , D. Falconer , and J. Bille . 2003. “Candida Albicans Mutations in the Ergosterol Biosynthetic Pathway and Resistance to Several Antifungal Agents.” Antimicrobial Agents and Chemotherapy 47: 2404–2412. 10.1128/AAC.47.8.2404-2412.2003. 12878497 PMC166068

[cbdd70045-bib-0251] Sant, D. G. , S. G. Tupe , C. V. Ramana , and M. V. Deshpande . 2016. “Fungal Cell Membrane—Promising Drug Target for Antifungal Therapy.” Journal of Applied Microbiology 121: 1498–1510. 10.1111/jam.13301. 27667746

[cbdd70045-bib-0252] Santangelo, R. , P. Paderu , G. Delmas , et al. 2000. “Efficacy of Oral Cochleate‐Amphotericin B in a Mouse Model of Systemic Candidiasis.” Antimicrobial Agents and Chemotherapy 44, no. 9: 2356–2360. 10952579 10.1128/aac.44.9.2356-2360.2000 PMC90069

[cbdd70045-bib-0253] Sass, G. , D. J. Larwood , M. Martinez , P. Shrestha , and D. A. Stevens . 2021. “Efficacy of Nikkomycin Z in Murine CNS Coccidioidomycosis: Modelling Sustained‐Release Dosing.” Journal of Antimicrobial Chemotherapy 76: 2629–2635. 10.1093/jac/dkab223. 34269392

[cbdd70045-bib-0254] Sawant, B. , and T. Khan . 2017. “Recent Advances in Delivery of Antifungal Agents for Therapeutic Management of Candidiasis.” Biomedicine and Pharmacotherapy 96: 1478–1490. 10.1016/j.biopha.2017.11.127. 29223551

[cbdd70045-bib-0255] Schaller, H. 2003. “The Role of Sterols in Plant Growth and Development.” Progress in Lipid Research 42: 163–175. 10.1016/S0163-7827(02)00047-4. 12689617

[cbdd70045-bib-0256] Schilling, A. , M. Seibold , V. Mansmann , and B. Gleissner . 2008. “Successfully Treated Candida Krusei Infection of the Lumbar Spine With Combined Caspofungin/Posaconazole Therapy.” Medical Mycology 46: 79–83. 10.1080/13693780701552996. 17852716

[cbdd70045-bib-0257] Schrettl, M. , E. Bignell , C. Kragl , et al. 2004. “Siderophore Biosynthesis but Not Reductive Iron Assimilation Is Essential for Aspergillus Fumigatus Virulence.” Journal of Experimental Medicine 200: 1213–1219. 10.1084/jem.20041242. 15504822 PMC2211866

[cbdd70045-bib-0258] Sen, A. , and D. Karati . 2024. “An Insight Into Thymidylate Synthase Inhibitor as Anticancer Agents: An Explicative Review.” Naunyn‐Schmiedeberg's Archives of Pharmacology 397: 5437–5448. 10.1007/s00210-024-03020-y. 38446215

[cbdd70045-bib-0259] Servatius, P. , and U. Kazmaier . 2018. “Total Synthesis of Trapoxin A, a Fungal HDAC Inhibitor From Helicoma Ambiens.” Journal of Organic Chemistry 83: 11341–11349. 10.1021/acs.joc.8b01569. 30079728

[cbdd70045-bib-0260] Sharma, N. , and D. Sharma . 2015. “An Upcoming Drug for Onychomycosis: Tavaborole.” Journal of Pharmacology and Pharmacotherapeutics 6: 236–239. 10.4103/0976-500X.171870. 26816482 PMC4714399

[cbdd70045-bib-0261] Shaw, K. J. , and A. S. Ibrahim . 2020. “Fosmanogepix: A Review of the First‐In‐Class Broad Spectrum Agent for the Treatment of Invasive Fungal Infections.” Journal of Fungi 6: 1–21. 10.3390/jof6040239. PMC7711534 33105672

[cbdd70045-bib-0262] Shende, P. , R. Khair , and R. S. Gaud . 2019. “Nanostructured Cochleates: A Multi‐Layered Platform for Cellular Transportation of Therapeutics.” Drug Development and Industrial Pharmacy 45: 869–881. 10.1080/03639045.2019.1583757. 30767577

[cbdd70045-bib-0263] Sigera, L. S. M. , and D. W. Denning . 2023. “Flucytosine and Its Clinical Usage.” Therapeutic Advances in Infectious Disease 10: 204993612311613. 10.1177/20499361231161387. PMC10084540 37051439

[cbdd70045-bib-0264] Simon, R. P. , D. Robaa , Z. Alhalabi , W. Sippl , and M. Jung . 2016. “KATching‐Up on Small Molecule Modulators of Lysine Acetyltransferases.” Journal of Medicinal Chemistry 59: 1249–1270. 10.1021/acs.jmedchem.5b01502. 26701186

[cbdd70045-bib-0265] Singh, A. , K. Singh , A. Sharma , K. Kaur , R. Chadha , and P. M. S. Bedi . 2023. “Recent Advances in Antifungal Drug Development Targeting Lanosterol 14α‐Demethylase (CYP51): A Comprehensive Review With Structural and Molecular Insights.” Chemical Biology and Drug Design 102: 606–639. 10.1111/cbdd.14266. 37220949

[cbdd70045-bib-0266] Singh, S. D. , N. Robbins , A. K. Zaas , W. A. Schell , J. R. Perfect , and L. E. Cowen . 2009. “Hsp90 Governs Echinocandin Resistance in the Pathogenic Yeast Candida Albicans via Calcineurin.” PLoS Pathogens 5: e1000532. 10.1371/journal.ppat.1000532. 19649312 PMC2712069

[cbdd70045-bib-0267] Skwarska, A. , E. D. D. Calder , D. Sneddon , et al. 2021. “Development and Pre‐Clinical Testing of a Novel Hypoxia‐Activated KDAC Inhibitor.” Cell, Chemistry and Biology 28: 1258–1270.e13. 10.1016/j.chembiol.2021.04.004. PMC8460716 33910023

[cbdd70045-bib-0268] Sokol‐Anderson, M. L. , J. Brajtburg , and G. Medoff . 1986. “Amphotericin B‐Induced Oxidative Damage and Killing of Candida Albicans.” Journal of Infectious Diseases 154: 76–83. 10.1093/infdis/154.1.76. 3519792

[cbdd70045-bib-0269] Sokolov, S. S. , N. I. Trushina , F. F. Severin , and D. A. Knorre . 2019. “Ergosterol Turnover in Yeast: An interplay Between Biosynthesis and Transport.” Biochemistry 84: 346–357. 10.1134/S0006297919040023. 31228926

[cbdd70045-bib-0270] Song, G. , G. Liang , and W. Liu . 2020. “Fungal co‐Infections Associated With Global COVID‐19 Pandemic: A Clinical and Diagnostic Perspective From China.” Mycopathologia 185: 599–606. 10.1007/s11046-020-00462-9. 32737747 PMC7394275

[cbdd70045-bib-0271] Spec, A. , J. Pullman , G. R. Thompson , et al. 2019. “MSG‐10: A Phase 2 Study of Oral Ibrexafungerp (SCY‐078) Following Initial Echinocandin Therapy in Non‐neutropenic Patients With Invasive Candidiasis.” Journal of Antimicrobial Chemotherapy 74: 3056–3062. 10.1093/jac/dkz277. 31304536

[cbdd70045-bib-0272] Su, H. , L. Han , and X. Huang . 2018. “Potential Targets for the Development of New Antifungal Drugs.” Journal of Antibiotics 71: 978–991. 10.1038/s41429-018-0100-9. 30242283

[cbdd70045-bib-0273] Suberviola, B. 2021. “Clinical Safety of Liposomal Amphotericin B.” Revista Iberoamericana de Micología 38: 56–60. 10.1016/j.riam.2021.02.001. 33994103

[cbdd70045-bib-0274] Sun, A. , N. Chai , X. Zhu , et al. 2023. “Optimization and Antifungal Activity of Quinoline Derivatives Linked to Chalcone Moiety Combined With FLC Against Candida Albicans.” European Journal of Medicinal Chemistry 260: 115782. 10.1016/j.ejmech.2023.115782. 37672929

[cbdd70045-bib-0275] Sun, B. , W. Liu , Q. Wang , et al. 2023. “Design, Synthesis, and Activity Evaluation of Novel Dual‐Target inhibitors With Antifungal and Immunoregulatory Properties.” Journal of Medicinal Chemistry 66: 13007–13027. 10.1021/acs.jmedchem.3c00942. 37705322

[cbdd70045-bib-0276] Takara Bio . 2024. “Aureobasidin A for Yeast Two‐Hybrid Studies.” https://www.takarabio.com/products/protein‐research/two‐hybrid‐and‐one‐hybrid‐systems/yeast‐media/aureobasidin‐a/.

[cbdd70045-bib-0277] Teixeira, M. M. , D. T. Carvalho , E. Sousa , and E. Pinto . 2022. “New Antifungal Agents With Azole Moieties.” Pharmaceuticals 15: 1427. 10.3390/ph15111427. 36422557 PMC9698508

[cbdd70045-bib-0278] Thompson, G. R. , A. Soriano , O. A. Cornely , et al. 2023. “Rezafungin Versus Caspofungin for Treatment of Candidaemia and Invasive Candidiasis (ReSTORE): A Multicentre, Double‐Blind, Double‐Dummy, Randomised Phase 3 Trial.” Lancet 401: 49–59. 10.1016/S0140-6736(22)02324-8. 36442484

[cbdd70045-bib-0279] Tom, K. 2017. “Assessment of the Duration of Infusion on the Tolerability and Repeat Dose Pharmacokinetics of F901318 in Healthy Volunteers.” https://f2g.com/wp‐content/uploads/2023/04/eccmid2017_poster_iv_mad.pdf/.

[cbdd70045-bib-0280] Touchstone, L. A. 2023. “New Antifungal Molecule Kills Fungi Without Toxicity in Human Cells, Mice.” https://www.sciencedaily.com/releases/2023/11/231108115031.htm.

[cbdd70045-bib-0281] Tu, B. , G. Yin , and H. Li . 2020. “Synergistic Effects of Vorinostat (SAHA) and Azoles Against Aspergillus Species and Their Biofilms.” BMC Microbiology 20: 28. 10.1186/s12866-020-1718-x. 32028887 PMC7006160

[cbdd70045-bib-0282] Upadhyay, S. , G. S. Jeena , S. Kumar , and R. K. Shukla . 2020. “Asparagus Racemosus bZIP Transcription Factor‐Regulated Squalene Epoxidase (ArSQE) Promotes Germination and Abiotic Stress Tolerance in Transgenic Tobacco.” Plant Science 290: 110291. 10.1016/j.plantsci.2019.110291. 31779892

[cbdd70045-bib-0283] Vale‐Silva, L. A. 2015. “Molecular Mechanisms of Resistance Of Candida spp. to Membrane Targeting Antifungals.” Antifungals: from genomics to resistance and the development of novel agents, 1–26. Pool, UK: Caister Academic Press.

[cbdd70045-bib-0284] Valley Fever Solutions . 2024. “Nikkomycin Z (NikZ) is First in a New Class of Antifungal.” https://www.valleyfeversolutions.com/.

[cbdd70045-bib-0285] Van Daele, R. , I. Spriet , J. Wauters , et al. 2019. “Antifungal Drugs: What Brings the Future.” Medical Mycology 57: S328–S343. 10.1093/mmy/myz012. 31292663

[cbdd70045-bib-0286] Vandeputte, P. , S. Ferrari , and A. T. Coste . 2012. “Antifungal Resistance and New Strategies to Control Fungal Infections.” International Journal of Microbiology 2012: 713687. 10.1155/2012/713687. 22187560 PMC3236459

[cbdd70045-bib-0287] Vanzolini, T. , and M. Magnani . 2024. “Old and New Strategies in Therapy and Diagnosis Against Fungal Infections.” Applied Microbiology and Biotechnology 108: 147. 10.1007/s00253-023-12884-8. 38240822 PMC10799149

[cbdd70045-bib-0288] Vermes, A. , H. J. Guchelaar , and J. Dankert . 2000. “Flucytosine: A Rewiev of Pharmacology, Clinical Indications, Pharmacocinetics, Toxicity and Drug Interactions.” Journal of Antimicrobial Chemoteraphy 46: 171–179. 10.1093/jac/46.2.171 10933638

[cbdd70045-bib-0289] Víglaš, J. , and P. Olejníková . 2021. “Signalling Mechanisms Involved in Stress Response to Antifungal Drugs.” Research in Microbiology 172: 103786. 10.1016/j.resmic.2020.10.001. 33038529

[cbdd70045-bib-0290] Vincent, B. M. , A. K. Lancaster , R. Scherz‐Shouval , L. Whitesell , and S. Lindquist . 2013. “Fitness Trade‐Offs Restrict the Evolution of Resistance to Amphotericin B.” PLoS Biology 11: e1001692. 10.1371/journal.pbio.1001692. 24204207 PMC3812114

[cbdd70045-bib-0291] Wang, R. , H. Sun , G. Wang , and H. Ren . 2020. “Imbalance of Lysine Acetylation Contributes to the Pathogenesis of Parkinson's Disease.” International Journal of Molecular Sciences 21: 7182. 10.3390/ijms21197182. 33003340 PMC7582258

[cbdd70045-bib-0292] Wang, X. , R. Yang , S. Liu , et al. 2021. “IMB‐XMA0038, a New Inhibitor Targeting Aspartate‐Semialdehyde Dehydrogenase of *Mycobacterium tuberculosis* .” Emerging Microbes and Infections 10: 2291–2299. 10.1080/22221751.2021.2006578. 34779708 PMC8648042

[cbdd70045-bib-0293] Wang, Z. , M. Liu , S. Shi , X. Zhou , C. Wu , and K. Wu . 2024. “Ti3C2Tx/Laser‐Induced Graphene‐Based Micro‐Droplet Electrochemical Sensing Platform for Rapid and Sensitive Detection of Benomyl.” Analytica Chimica Acta 1304: 342526. 10.1016/j.aca.2024.342526. 38637046

[cbdd70045-bib-0294] Ward, D. M. , O. S. Chen , L. Li , et al. 2018. “Altered Sterol Metabolism in Budding Yeast Affects Mitochondrial Iron–Sulfur (Fe‐S) Cluster Synthesis.” Journal of Biological Chemistry 293: 10782–10795. 10.1074/jbc.RA118.001781. 29773647 PMC6036212

[cbdd70045-bib-0295] Wassano, N. S. , A. B. Leite , F. Reichert‐Lima , A. Z. Schreiber , N. S. Moretti , and A. Damasio . 2020. “Lysine Acetylation as Drug Target in Fungi: An Underexplored Potential in Aspergillus spp.” Brazilian Journal of Microbiology 51: 673–683. 10.1007/s42770-020-00253-w. 32170592 PMC7203240

[cbdd70045-bib-0296] Watanabe, N. A. , M. Miyazaki , T. Horii , K. Sagane , K. Tsukahara , and K. Hata . 2012. “E1210, a New Broad‐Spectrum Antifungal, Suppresses Candida Albicans Hyphal Growth Through Inhibition of Glycosylphosphatidylinositol Biosynthesis.” Antimicrobial Agents and Chemotherapy 56: 960–971. 10.1128/AAC.00731-11. 22143530 PMC3264227

[cbdd70045-bib-0297] Wen, W. , H. Cao , Y. Huang , et al. 2022. “Structure‐Guided Discovery of the Novel Covalent Allosteric Site and Covalent Inhibitors of Fructose‐1,6‐Bisphosphate Aldolase to Overcome the Azole Resistance of Candidiasis.” Journal of Medicinal Chemistry 65: 2656–2674. 10.1021/acs.jmedchem.1c02102. 35099959

[cbdd70045-bib-0298] Whaley, S. G. , E. L. Berkow , J. M. Rybak , A. T. Nishimoto , K. S. Barker , and P. D. Rogers . 2017. “Azole Antifungal Resistance in *Candida albicans* and Emerging Non‐albicans Candida Species.” Frontiers in Microbiology 7: 2173. 10.3389/fmicb.2016.02173. 28127295 PMC5226953

[cbdd70045-bib-0299] Wiederhold, N. P. 2022. “Pharmacodynamics, Mechanisms of Action and Resistance, and Spectrum of Activity of New Antifungal Agents.” Journal of Fungi 8: 857. 10.3390/jof8080857. 36012845 PMC9410397

[cbdd70045-bib-0300] Wiederhold, N. P. , L. K. Najvar , A. W. Fothergill , et al. 2016. “The Novel Arylamidine T‐2307 Demonstrates In Vitro and In Vivo Activity Against Echinocandin‐Resistant Candida Glabrata.” Journal of Antimicrobial Chemotherapy 71: 692–695. 10.1093/jac/dkv398. 26620102 PMC4743701

[cbdd70045-bib-0301] Wiederhold, N. P. , L. K. Najvar , R. Jaramillo , et al. 2018. “The Orotomide Olorofim Is Efficacious in an Experimental Model of Central Nervous System Coccidioidomycosis.” Antimicrobial Agents and Chemotherapy 62: e00999‐18. 10.1128/AAC.00999-18. 29941638 PMC6125497

[cbdd70045-bib-0302] Wiederhold, N. P. , L. F. Shubitz , L. K. Najvar , et al. 2018. “The Novel Fungal Cyp51 Inhibitor VT‐1598 is Efficacious in Experimental Models of Central Nervous System Coccidioidomycosis Caused by *Coccidioides posadasii* and *Coccidioides immitis* .” Antimicrobial Agents and Chemotherapy 62: e02258‐17. 10.1128/AAC.02258-17. 29437615 PMC5913997

[cbdd70045-bib-0303] Wiederhold, N. P. , X. Xu , A. Wang , et al. 2018. “In Vivo Efficacy of VT‐1129 Against Experimental Cryptococcal Meningitis With the Use of a Loading Dose‐Maintenance Dose Administration Strategy.” Antimicrobial Agents and Chemotherapy 62: e01315‐18. 10.1128/AAC.01315-18. 30104280 PMC6201077

[cbdd70045-bib-0304] Wirth, F. , and K. Ishida . 2020. “Antifungal Drugs: An Updated Review of Central Nervous System Pharmacokinetics.” Mycoses 63: 1047–1059. 10.1111/myc.13157. 32772402

[cbdd70045-bib-0305] Wu, S. , R. Song , T. Liu , and C. Li . 2023. “Antifungal Therapy: Novel Drug Delivery Strategies Driven by New Targets.” Advanced Drug Delivery Reviews 199: 114967. 10.1016/j.addr.2023.114967. 37336246

[cbdd70045-bib-0306] Yamashita, K. , T. Miyazaki , Y. Fukuda , et al. 2019. “The Novel Arylamidine T‐2307 Selectively Disrupts Yeast Mitochondrial Function by Inhibiting Respiratory Chain Complexes.” Antimicrobial Agents and Chemotherapy 63: e00374‐19. 10.1128/AAC.00374-19. 31182539 PMC6658782

[cbdd70045-bib-0307] Yan, Y. , X. Xie , W. Jiang , et al. 2024. “Novel Pyrido [4,3‐ d] Pyrimidine Derivatives as Potential Sterol 14α‐Demethylase Inhibitors: Design, Synthesis, Inhibitory Activity, and Molecular Modeling.” Journal of Agricultural and Food Chemistry 72: 12260–12269. 10.1021/acs.jafc.3c09543. 38759097

[cbdd70045-bib-0308] Yan, Z. , Y. Huang , D. Zhao , et al. 2023. “Developing Novel Coumarin‐Containing Azoles Antifungal Agents by the Scaffold Merging Strategy for Treating Azole‐Resistant Candidiasis.” Journal of Medicinal Chemistry 66: 13247–13265. 10.1021/acs.jmedchem.3c01254. 37725043

[cbdd70045-bib-0309] Yang, Z. , R. C. Pascon , J. A. Alspaugh , G. M. Cox , and J. H. Mccusker . 2002. “Molecular and Genetic Analysis of the *Cryptococcus neoformans* MET3 Gene and a met3 Mutant.” Microbiology 148, no. Pt 8: 2617–2625. 12177356 10.1099/00221287-148-8-2617

[cbdd70045-bib-0310] Yeğenoğlu, Y. 2012. “Antifungal Direnci Gösteren Mantarlar.”

[cbdd70045-bib-0311] Yin, W. , T. Wu , L. Liu , et al. 2022. “Species‐Selective Targeting of Fungal Hsp90: Design, Synthesis, and Evaluation of Novel 4,5‐Diarylisoxazole Derivatives for the Combination Treatment of Azole‐Resistant Candidiasis.” Journal of Medicinal Chemistry 65: 5539–5564. 10.1021/acs.jmedchem.1c01991. 35298171

[cbdd70045-bib-0312] Youssef, M. E. , S. Cavalu , A. M. Hasan , G. Yahya , M. A. Abd‐Eldayem , and S. Saber . 2023. “Role of Ganetespib, an hsp90 Inhibitor, in Cancer Therapy: From Molecular Mechanisms to Clinical Practice.” International Journal of Molecular Sciences 24: 5014. 10.3390/ijms24055014. 36902446 PMC10002602

[cbdd70045-bib-0313] Yuzugullu Karakus, Y. 2020. “Typical Catalases: Function and Structure.” In Glutathione System and Oxidative Stress in Health and Disease. London: IntechOpen. 10.5772/intechopen.90048.

[cbdd70045-bib-0314] Zang, T. , S. Wang , S. Su , et al. 2021. “Off–on Squalene Epoxidase‐Specific Fluorescent Probe for Fast ımaging in Living Cells.” Analytical Chemistry 93: 14716–14721. 10.1021/acs.analchem.1c03168. 34702029

[cbdd70045-bib-0315] Zaugg, C. , M. Monod , J. Weber , et al. 2009. “Gene Expression Profiling in the Human Pathogenic Dermatophyte *Trichophyton rubrum* During Growth on Proteins.” Eukaryotic Cell 8: 241–250. 10.1128/EC.00208-08. 19098130 PMC2643602

[cbdd70045-bib-0316] Zhang, J. , H. Li , Y. Liu , et al. 2022. “Targeting Hsp90 as a Novel Therapy for Cancer: Mechanistic Insights and Translational Relevance.” Cells 11: 2778. 10.3390/cells11182778. 36139353 PMC9497295

[cbdd70045-bib-0317] Zhang, L. , Z. Cao , Y. Hong , et al. 2024. “Squalene Epoxidase: Its Regulations and Links With Cancers.” International Journal of Molecular Sciences 25: 3874. 10.3390/ijms25073874. 38612682 PMC11011400

[cbdd70045-bib-0318] Zhang, P. , Z. Fang , Y. Song , et al. 2022. “Aspartate Transaminase AST2 Involved in Sporulation and Necrotrophic Pathogenesis in the Hemibiotrophs *Magnaporthe oryzae* and *Colletotrichum graminicola* .” Frontiers in Microbiology 13: 864866. 10.3389/fmicb.2022.864866. 35479642 PMC9037547

[cbdd70045-bib-0319] Zhang, Z. , G. F. Bills , and Z. An . 2023. “Advances in the Treatment of Invasive Fungal Disease.” PLoS Pathogens 19: e1011322. 10.1371/journal.ppat.1011322. 37141208 PMC10159104

[cbdd70045-bib-0320] Zhao, Y. , B. Prideaux , Y. Nagasaki , et al. 2017. “Unraveling Drug Penetration of Echinocandin Antifungals at the Site of Infection in an Intra‐Abdominal Abscess Model.” Antimicrobial Agents and Chemotherapy 61: e01009‐17. 10.1128/AAC.01009-17. 28739797 PMC5610477

[cbdd70045-bib-0321] Zheng, L. , Y. Xu , Y. Dong , et al. 2023. “Chromosome 1 Trisomy Confers Resistance to Aureobasidin A in Candida Albicans.” Frontiers in Microbiology 14: 1128160. 10.3389/fmicb.2023.1128160. 37007527 PMC10063858

[cbdd70045-bib-0322] Zhou, M. , L. Liu , Z. Cong , et al. 2024. “A Dual‐Targeting Antifungal Is Effective Against Multidrug‐Resistant Human Fungal Pathogens.” Nature Microbiology 9: 1325–1339. 10.1038/s41564-024-01662-5. 38589468

[cbdd70045-bib-0323] Zhou, Y. , M. Liao , C. Zhu , et al. 2018. “ERG3 and ERG11 Genes Are Critical for the Pathogenesis of *Candida albicans* During the Oral Mucosal Infection Article.” International Journal of Oral Science 10: 9. 10.1038/s41368-018-0013-2. 29555898 PMC5944255

[cbdd70045-bib-0324] Zhou, Y. , X. Liu , J. Xue , et al. 2020. “Discovery of Peptide Boronate Derivatives as Histone Deacetylase and Proteasome Dual Inhibitors for Overcoming Bortezomib Resistance of Multiple Myeloma.” Journal of Medicinal Chemistry 63: 4701–4715. 10.1021/acs.jmedchem.9b02161. 32267687

[cbdd70045-bib-0325] Zou, Y. , L. Liu , J. Liu , and G. Liu . 2020. “Bioisosteres in Drug Discovery: Focus on Tetrazole, Future.” Medicinal Chemistry 12: 91–93. 10.4155/fmc-2019-0288. 31762337

[cbdd70045-bib-0326] Zou, Y. , H. Zhang , F. Bi , Q. Tang , and H. Xu . 2022. “Targeting the Key Cholesterol Biosynthesis Enzyme Squalene Monooxygenasefor Cancer Therapy.” Frontiers in Oncology 12: 938502. 10.3389/fonc.2022.938502. 36091156 PMC9449579

[cbdd70045-bib-0327] Zung, N. , N. Aravindan , A. Boshnakovska , et al. 2024. “The Molecular Mechanism of On‐Demand Sterol Biosynthesis at Organelle Contact Sites.”

[cbdd70045-bib-0328] Zwick, V. , P. M. Allard , L. Ory , et al. 2017. “UHPLC–MS‐Based HDAC Assay Applied to Bio‐Guided Microfractionation of Fungal Extracts.” Phytochemical Analysis 28: 93–100. 10.1002/pca.2652. 27921344

